# Higher prevalence of cytomegalovirus and Epstein–Barr virus in acute-on-chronic liver failure

**DOI:** 10.1016/j.jhepr.2025.101627

**Published:** 2025-10-09

**Authors:** Keerthihan Thiyagarajah, Jannik Sonnenberg, Esra Görgülü, Pia Lembeck, Nico Kraus, Mirco Glitscher, Frank Erhard Uschner, Maximilian Joseph Brol, Wenyi Gu, Robert Schierwagen, Sabine Klein, Martin S. McCoy, Marcus Maximilian Mücke, Toska Wiedemann, Philipp A. Reuken, Johanna Reißing, Franziska Schneider, Nina Böhling, Michael Praktiknjo, Phil-Robin Tepasse, Julia Fischer, Stefan Zeuzem, Christoph Welsch, Sandra Ciesek, Andreas Stallmach, Jonel Trebicka, Johannes Chang, Tony Bruns, Eberhard Hildt, Kai-Henrik Peiffer

**Affiliations:** 1 Paul-Ehrlich-Institut, Langen, Germany; 2 Goethe University Frankfurt, Medical Clinic 1, University Hospital, Frankfurt am Main, Germany; 3 Medizinische Klinik B, University of Münster, Münster, Germany; 4 Department of Gastroenterology, Ren Ji Hospital, School of Medicine, Shanghai Jiao Tong University, Shanghai, China; 5 Klinik für Innere Medizin IV, Jena University Hospital, Friedrich Schiller University Jena, Jena, Germany; 6 Medizinische Klinik III, RWTH Aachen University, Aachen, Germany; 7 Department of Internal Medicine I, University Hospital Bonn, Bonn, Germany; 8 Institut für Medizinische Virologie, Goethe University Frankfurt, Frankfurt, Germany; 9 Hasso-Plattner-Institute-Digital Health Cluster, University Potsdam, Potsdam, Germany

**Keywords:** Acute-on-chronic liver failure, Cytomegalovirus, Epstein–Barr virus, Human herpesvirus, Precipitant, Sustainer

## Abstract

**Background & Aims:**

Acute-on-chronic liver failure (ACLF) is a life-threatening syndrome characterized by rapid deterioration of organ function in pre-existing chronic liver disease. Patients who develop ACLF within 90 days after decompensation are referred to as pre-ACLF. Known precipitants include bacterial infections and viral hepatitis. However, in 40–60% of patients, the precipitant remains unknown. Cytomegalovirus (CMV) and Epstein–Barr virus (EBV) are highly prevalent viruses, but their impact on ACLF is unclear.

**Methods:**

211 patients (43 ACLF, 16 pre-ACLF, and 152 non-ACLF) of the ACLF-I study and an external validation cohort with 153 patients (39 ACLF, 33 pre-ACLF, and 81 non-ACLF) were included. Sera were analyzed for CMV/EBV DNA (multiplex qPCR), cytokines in 102 ACLF-I samples (multiplex assays), and immunoglobulins in 80 case-control matched ACLF-I and 20 validation cohort patients (immunoassays), and correlated with clinical data.

**Results:**

In the ACLF-I group, a higher prevalence of CMV DNAemia (8.9% *vs.* 25.6%, odds ratio [OR] 3.51, 95% CI 1.47–8.34, *p* <0.01) and EBV DNAemia (6.6% *vs.* 16.3%, OR 3.01, 95% CI 1.10–8.62, *p* <0.05) was observed in ACLF compared to non-ACLF cases, despite the absence of clinical signs of viral infections. 54.8% of DNAemic ACLF patients in the validation cohort had no identified precipitant compared to 26.5% in ACLF patients without DNAemia (*p* <0.05). CMV was associated with liver failure (*p* <0.001) and with 90-day mortality (*p* <0.001) in the regression model. DNAemia was associated with a distinct pattern of inflammatory activity. The results were validated externally. Serological analyses revealed that reactivation rather than primary infection occurred in most cases defined as DNAemic.

**Conclusions:**

Presence of CMV/EBV DNAemia in chronic liver disease may contribute to the development of ACLF by exacerbating liver inflammation and impairing hepatocellular function.

**Impact and implications:**

Despite ACLF being a life-threatening syndrome, the underlying precipitant cannot be identified in 30–40% of cases. As a result, intervention in ACLF development is limited to cases with known precipitants. To our knowledge, this is the first time it is shown that previously undiagnosed CMV and EBV DNAemia might act as a precipitating event inducing ACLF. We propose that routine screening and proper treatment for CMV and EBV in decompensated cirrhotic patients can intervene in ACLF development. This hypothesis needs to be assessed in prospective studies.

**Clinical trials registration:**

NCT04975490.

## Highlights

• CMV and EBV DNAemia are more frequent in patients with ACLF than in non-ACLF patients.
• DNAemia is associated with disease severity and a distinct inflammatory pattern.
• CMV correlates with liver failure, whereas EBV is associated with mechanical ventilation.
• Pre-ACLF is showing increased frequencies of CMV/EBV, although the results are not significant.
• CMV/EBV may be a trigger or sustainer of ACLF, but further research is needed to reach a definitive conclusion.

## Graphical abstract

**Figure:**
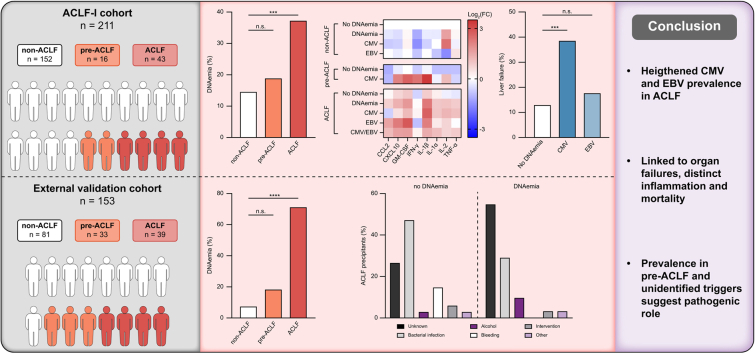

## Introduction

Liver cirrhosis and its complications were associated with 2.4% of global deaths in 2017, being prevalent in 112 million patients.^1^ Acute-on-chronic liver failure (ACLF) is increasingly recognized as an important and distinct syndrome. Yet, multiple definitions are in use, generating a confusing situation for clinicians.^2^^,^^3^ According to the EASL-Chronic Liver Failure Consortium (CLIF-C), it is characterized by the functional failure of one or more of six major organ systems (liver, kidney, brain, coagulation, circulation, and respiration) in patients with chronic liver disease.^4^^,^^5^ It is associated with a 28-day mortality rate of at least 20% and up to 80% depending on the number of organ failures.^5^ Known possible precipitants include systemic infections, invasive procedures, gastrointestinal hemorrhage, alcohol consumption, viral hepatitis, drug-induced liver injury, and autoimmune-related events.^6^ Still, a precipitating event cannot be identified in 30–40% and the rate of unidentified precipitants exceeding 40% in the CANONIC study.^5^ The distinctive spectrum of immune alterations associated with end-stage liver disease is called cirrhosis-associated immune dysfunction (CAID).^7^ Affected patients are at an increased risk of developing clinically significant infections that are typically regarded as less pathogenic.

The EASL-CLIF-C defines pre-ACLF as the rapid development of ACLF within 90 days after decompensation of chronic liver disease.^8^ The central hallmark of pre-ACLF is high systemic inflammation, as observed in the PREDICT study.^9^ Over the past few years, systemic inflammation has gained increasing attention as a predictor of ACLF, being more relevant for outcomes than circulatory dysfunction.^10^ Systemic inflammation may impair the function of organ systems and cause immune-mediated tissue damage and metabolic changes.^11^ It has recently even been shown to be a stronger predictor for bleeding than coagulopathy in acutely decompensated patients.^12^

Human herpesvirus (HHV) infections/reactivations are known precipitants of viral hepatitis and acute liver failure.^13^^,^^14^ HHVs, like Cytomegalovirus (CMV) and Epstein–Barr virus (EBV), are highly prevalent, with 40–100% of populations being carriers of latent infections.^15^^,^^16^ While in immunocompetent patients primary EBV infection has been shown to be a very rare cause of acute liver failure, CMV has not been convincingly linked to acute liver failure, to the best of our knowledge.^17^^,^^18^ Yet, immunocompromised patients may develop fulminant hepatitis culminating in acute liver failure and the need for transplantation.^19^^,^^20^ Furthermore, CMV and EBV have been described to be present in acutely decompensated liver cirrhosis and acute-on-chronic liver failure in case studies, lacking proof of a causal relation.^21^ By modulating the innate immune system and suppressing host cell apoptosis, HHVs promote their own persistence, while active replication leads to increased cytokine production and immune cell activation.^22^^,^^23^ As treatable factors, they may provide an opportunity to alleviate the clinical course of ACLF.

Therefore, this study aims to investigate the role of CMV and EBV in the context of ACLF. Patients with chronic liver disease from two cohorts from different German university hospitals were included and stratified regarding their disease trajectories as non-ACLF (compensated cirrhosis, stable decompensated cirrhosis/unstable decompensated cirrhosis), pre-ACLF, and ACLF. Findings from blood serum samples were subsequently correlated with clinical data gained throughout the treatment.

## Materials and methods

### Study design

In this retrospective analysis of the ongoing prospective longitudinal study Characterization and Pathogenesis of ACLF (ACLF-I, NCT04975490, Medical Faculty of Goethe University Frankfurt, No. 20-653) adult patients admitted to the Department of Internal Medicine I, University Hospital Frankfurt, Frankfurt, Germany, between 2021 and 2023 were included. Patients with liver cirrhosis who were admitted planned or unplanned were eligible for inclusion in the ACLF-I. Blood samples were collected at the time of inclusion. In total, 211 patients were included in this sub analysis of the ACLF-I, among these 43 presented with ACLF. Furthermore, 16 of the included patients developed ACLF within 90 days and were classified as pre-ACLF.

The following parameters were assessed for the ACLF-I patients: age, sex, aetiology of cirrhosis, ACLF/pre-ACLF, ACLF grade, 28-day and 90-day mortality, model for end-stage liver disease (MELD), MELD including sodium (MELDNa), Child-Pugh score, CLIF-C organ failure (OF) score, CLIF-C acute decompensation (AD) score, CLIF-C ACLF score, liver failure, renal failure, cerebral failure, coagulation failure, circulation failure, respiratory failure, sodium, potassium, creatinine, albumin, leucocytes, C-reactive protein (CRP), bilirubin, international normalized ratio (INR), aspartate aminotransferase (AST), alanine aminotransferase (ALT), alkaline phosphatase, gamma-GT, renal replacement treatment, ascites, hepatic encephalopathy, West Haven grade of hepatic encephalopathy, gastrointestinal hemorrhage, bacterial infections, viral infection, immunosuppression, medication, supplemental oxygen, and mechanical ventilation.

Data from two cohorts of patients from Germany with and without ACLF as well as pre-ACLF were included as an external validation cohort. The cohorts comprised 78 patients hospitalised for acute decompensation with ascites prior to puncture admitted to the Department of Internal Medicine III, University Hospital Aachen, Aachen, Germany, or the Department of Internal Medicine IV, Jena University Hospital, Jena, Germany, between 2010 and 2019 (33 non-ACLF, 7 pre-ACLF, 38 ACLF; Internal Review Boards: Jena University Hospital No. 683-02/3 and 2880-08/10; RWTH Aachen No. 327/19). A further 75 patients admitted to the Department of Medicine I, University Hospital Bonn, Bonn, Germany, between 2019 and 2023 with decompensated liver cirrhosis were included (49 non-ACLF and 26 pre-ACLF; Ethics review board No. 288/11, NCT04393519). Serum samples gathered at the time of inclusion were analysed retrospectively. Additionally, the ACLF precipitant was assessed. The pre-ACLF cohort from Bonn included the time to ACLF.

Written informed consent was obtained from each patient included in the study and the study protocols conform to the ethical guidelines of the 1975 Declaration of Helsinki, as reflected in the *a priori* approvals by the institutions’ human research committees.

The diagnosis of cirrhosis was based on previous liver biopsy findings or a composite of clinical signs and findings provided by laboratory test results, endoscopy, and imaging. Diagnostic criteria for ACLF were based on the CANONIC study criteria.^5^ Organ failure and organ dysfunction were defined according to the CLIF-C OF score, respectively failure of the liver, kidneys, brain, coagulation, circulatory, and respiratory system.^5^

### Sample analysis

Samples within each center were handled and stored in the same way. After a blood sample was drawn, the samples were aliquoted and frozen at -80 °C within 2 h. Due to the process of aliquotation, freeze–thaw cycles were kept to a minimum prior to analysis and were identical within each cohort (see the supplementary data for further information).

#### CMV and EBV detection

##### Viral DNA extraction

Viral nucleic acids of blood sera were extracted using the High Pure Viral Nucleic Acid Kit (Roche Diagnostics, Mannheim, Germany) according to manufactureŕs instructions. Blood sera were previously spiked with Phocine herpesvirus (PhHV) DNA (Roche Diagnostics) to monitor extraction efficiency. 200 μl of blood sera was used as input and viral DNA was concentrated fourfold to a final concentration of 50 μl per sample during extraction.

##### Multiplex qPCR

Multiplex real-time quantitative PCR (qPCR) of extracted DNA samples was performed by using TaqMan hydrolysis probes (Roche Diagnostics) and the LightCycler Multiplex DNA master (Roche Diagnostics). 8 μl of master mix (Table S1) were mixed with 12 μl of extracted DNA sample to obtain a final reaction volume of 20 μl. The multiplex-qPCR was carried out in a Lightcycler 480 instrument II (Roche Diagnostics) following the recommended qPCR program for the respective TaqMan hydrolysis probes provided by the manufacturer. All samples were measured in duplicates.

##### Determination of serum viral loads

Multiplex-qPCR results were analysed using the Lightcycler 480 software. All qPCR data were colour compensated prior to analysis to correct results for crosstalk. Respective cycle threshold (Ct) values were determined by applying the Second Derivative maximum algorithm.

To determine the lower limit of detection (LLOD), a serial dilution ranging from 2 to 10^6^ copies per reaction was performed. Seven copies per reaction was the lowest copy number detected in all replicates with more than 95% confidence. The calculated coefficient of variation of the Ct values (CV Ct) was 0.92% and 1.26% for CMV and EBV, respectively. Thus, seven copies per reaction was set as the LLOD. During each run, positive controls were run alongside to assess interassay variation. The interassay CV Ct was 1.69% and 0.94% for the CMV and EBV-positive controls throughout all runs. Respective Ct values were converted into total copy numbers using a standard curve ranging from 10 to 10^6^ target molecules per reaction, representing the linear working range, as well as the lower and upper limit of quantification (LLOQ, ULOQ). The coefficient of determination for both standard curves was >0.99 (Fig. S1). Determined total copy numbers were translated into respective viral loads in blood serum using Equation 1:

[1] Viralload(copies/ml)=(totalcopies/reaction)/(0.012mlx4)

Accordingly, seven copies per reaction, presenting the LLOD, was equal to ∼150 copies per ml blood serum. Hence, the cut-off value for samples to be considered as DNAemia for all clinical samples was set to 150 copies per ml for both viruses. The analysis was conducted in the same manner for samples from every cohort.

#### Cytokine analysis

Samples were processed within 2 h of collection by centrifugation at 2,000 × *g* for 10 min at 4 °C. The blood serum supernatant was aliquoted and stored at -80 °C until analysis. Haemolysed or lipemic samples were excluded from the study.

A custom multiplex magnetic bead panel (Luminex Discovery Assay Human Premixed Multi-Analyte Kit, Catalog No. LXSAHM-16, Luminex Corporation, Austin, TX, USA) was used to quantify inflammatory and immune-related cytokines. The assay was performed using the Luminex MAGPIX system according to the manufacturer's instructions. All samples were measured in duplicates.

Serum samples were thawed on ice and diluted 1:2 with assay buffer. Negative controls and internally standardised samples were included in every assay as quality controls and to adjust for background noise. Magnetic beads coated with specific capture antibodies were added to a 96-well plate and incubated with 50 μl of each sample for 2 h at room temperature under constant agitation. Following incubation, plates were washed three times using a magnetic plate washer to remove unbound components. Afterwards, biotinylated detection antibodies were added and incubated for 1 h at room temperature, followed by streptavidin–phycoerythrin conjugate incubation for 30 min. Following a final washing step, beads were resuspended in assay buffer and median fluorescence intensities were measured using the Luminex MAGPIX system.

Data were acquired using xPONENT software (Luminex Corporation) and subsequently analysed. Assay performance was assessed by intra-assay CV, with a threshold of <15% for acceptance. Samples with mean fluorescence intensity below zero after subtraction of analyte blanks were assigned a mean fluorescence intensity of zero. The detection limit was defined by a minimum of bound magnetic beads.

#### Serological analysis

Due to a lack of sample availability and volume, only a subpopulation was included in this analysis. Within the ACLF-I cohort, all cases of ACLF or DNAemia were included. However, the CMV DNAemic subgroup was limited to clinically relevant cases with CMV DNAemia above 600 copies/ml. Control samples were matched according to disease trajectory and severity scores, including all ACLF patients. Aditionally, pre-ACLF patients from the Bonn cohort were analysed using the same case-control approach. Anti-EBV and anti-CMV antibodies were assessed retrospectively using the Abbott EBV-VCA IgM, EBV EBNA-1 IgG, CMV IgM, and CMV IgG test kits on the Abbott Architect i1000SRTM platform (Abbott GmbH, Wiesbaden, Germany), according to the manufacturer's specifications.

### Statistical analysis

Statistical analysis was performed using IBM SPSS Statistics for Windows, Version 29.0.0.0 (IBM Corporation, Armonk, NY, USA). Uni- and multivariable models were applied to identify correlations with CMV and EBV DNAemia and exclude possible confounding factors. In univariate statistical comparisons, tests against nominal variables were performed regarding the respective variable levels using Pearson Chi-square test for nominal variables, biserial correlation using Spearman-Rho coefficients for ordinal variables and *t* or Mann–Whitney *U* tests for metric variables. Whether normal distribution of metric variables could be assumed was tested by the Shapiro–Wilk test. Multiple linear regression using the backwards method was performed including variables with *p* <0.3 and variables of clinical relevance (see Table S2 for exact models). In all statistical analyses, significance was set at *p* <0.05. While initial analysis for virus prevalence were conducted including the full cohort, subgroup analysis for CMV and EBV were performed excluding the respective other group. Cases with missing data were excluded from the respective analyses (Table S10). As the presented study is a sub-analysis of the ongoing ACLF-I clinical observation study, no sample size calculation to determine an adequate power to detect a prespecified effect size was performed.

## Results

### Patient characteristics

211 patients from the ACLF-I cohort with liver cirrhosis with or without ACLF were included. The cohort comprised 43 ACLF, 16 pre-ACLF and 152 non-ACLF patients. There were significant differences between the groups of patients with and without DNAemia of CMV or EBV regarding sex (26.5% *vs.* 46.3%, *p* = 0.013) and arterial hypertension (30.6% *vs.* 9.8%, *p* = 0.007) (data not shown). Immunosuppressive therapy showed no significant correlation with the presence of DNAemia (for sensitivity analyses, see Table S8). Detailed patient characteristics are depicted in Table 1. ACLF was present in 43 patients (20.4%, mean CLIF-C OF score, 10.1).

**Table 1:** Patient characteristics of the ACLF-I cohort.

Characteristic	No DNAemia	CMV and/or EBV DNAemia	*p* value
**N**	**170**	**41**	–
Age, years, mean ± SD	58.6 ± 12.0	55.5 ± 14.4	0.077
Female sex, n (%)	45 (26.5)	19 (46.3)	0.013

**Acute-on-chronic liver failure, n (%)**			
ACLF	27 (15.9)	16 (39.0)	<0.001
Pre-ACLF	13 (7.6)	3 (7.3)	0.943
Liver failure	22 (12.9)	12 (29.3)	0.011
Renal failure	16 (9.4)	5 (12.2)	0.593
Cerebral failure	6 (3.5)	5 (12.2)	0.025
Coagulation failure	11 (6.5)	4 (9.8)	0.462
Circulatory failure	18 (10.6)	6 (14.6)	0.472
Respiratory failure	5 (2.9)	3 (7.3)	0.188

**Etiology of cirrhosis, n (%)**			
Alcohol	88 (51.8)	25 (61.0)	0.288
MASH	21 (12.4)	4 (9.8)	0.644
Cholestatic liver disease	19 (11.2)	2 (4.9)	0.227
HCV	13 (7.6)	0 (0)	0.068
HBV (±HDV)	7 (4.1)	2 (4.9)	0.829
Autoimmune hepatitis	5 (2.9)	2 (4.9)	0.534
Other etiologies	17 (10.0)	6 (14.6)	0.393

**Laboratory values, median (IQR)**			
Creatinine, mg/L	1.13 (0.74)	1.14 (0.81)	0.997
Bilirubin, mg/dl	2.50 (4.80)	2.80 (15.20)	0.115
AST, U/L	61.0 (61.0)	80.50 (36.0)	0.066
ALT, U/L	34.0 (34.0)	33.50 (25.0)	0.792
INR	1.46 (0.72)	1.46 (0.53)	0.768
Albumin, g/dl	3.20 (1.0)	3.0 (0.70)	0.158
CRP, mg/L	1.74 (3.09)	3.40 (3.60)	0.068
White blood cell count, x 10^9^/L	6.06 (5.04)	8.70 (8.0)	0.003

**Disease severity scores, median (IQR)**			
MELD	17 (11)	18 (15)	0.093
MELDNa	19 (12)	20 (17)	0.107
Child-Pugh score	9 (3)	9 (3)	0.147
CLIF-C ACLF	38 (12)	42 (14)	0.055
CLIF-C AD	60 (32)	71 (48)	0.069
CLIF-C OF	7 (2)	7 (4)	0.057

**Clinical data, n (%)**			
Diabetes mellitus	57 (33.5)	9 (22.0)	0.151
COPD	7 (4.1)	1 (2.4)	0.613
Heart failure	6 (3.5)	1 (2.4)	0.726
Arterial hypertension	52 (30.6)	4 (9.8)	0.007
Coronary artery disease	15 (8.8)	1 (2.4)	0.166
Chronic kidney disease	5 (2.9)	0 (0)	0.266
HCC	21 (12.4)	3 (7.3)	0.362
Immunosuppression∗	14 (8.2)	5 (12.2)	0.299
In patients with ACLF	4 (2.4)	2 (4.9)	0.887
In patients with pre-ACLF	1 (0.6)	1 (2.4)	0.226
Ascites	100 (58.8)	29 (70.7)	0.160
Hepatic encephalopathy	40 (23.5)	14 (34.1)	0.162
Gastrointestinal hemorrhage	39 (22.9)	5 (12.2)	0.128

**Outcome, n (%)**			
28-day mortality	15 (8.8)	6 (14.6)	0.265
90-day mortality	33 (19.4)	8 (19.5)	0.988
Liver transplantation	12 (7.1)	1 (2.4)	0.269

The no DNAemia and CMV and/or EBV DNAemia subgroups of patients at the time of inclusion, organ failure definitions according to EASL CLIF-C (Pearson‘s Chi-square test, biserial rank-correlation, Mann-Whitney *U* test or *t* test depending on variable level). ∗Immunosuppressive regimes included azathioprine (n = 6), mycophenolate mofetil (n = 2) and corticosteroids (n = 12) with one patient receiving mycophenolate mofetil and corticosteroids (indications: autoimmune hepatitis [n = 10], systemic sclerosis [n = 1], rheumatoid arthritis [n = 1], Coombs-negative hemolysis [n = 1], and undocumented [n = 6]). ACLF, acute-on-chronic liver failure; AD, acute decompensation; ALT, alanine aminotransferase; AST, aspartate aminotransferase; CLIF-C, Chronic Liver Failure Consortium; CMV, cytomegalovirus; COPD, chronic obstructive pulmonary disease; CRP, C-reactive protein; EBV, Epstein–Barr virus; HCC, hepatocellular carcinoma; INR, international normalized ratio; MASH, metabolic-associated steatohepatitis; MELD, model of end-stage liver disease; MELDNa, model of end-stage liver disease including sodium; OF, organ failure.

### Higher prevalence of DNAemia in ACLF

**Fig. 1:**
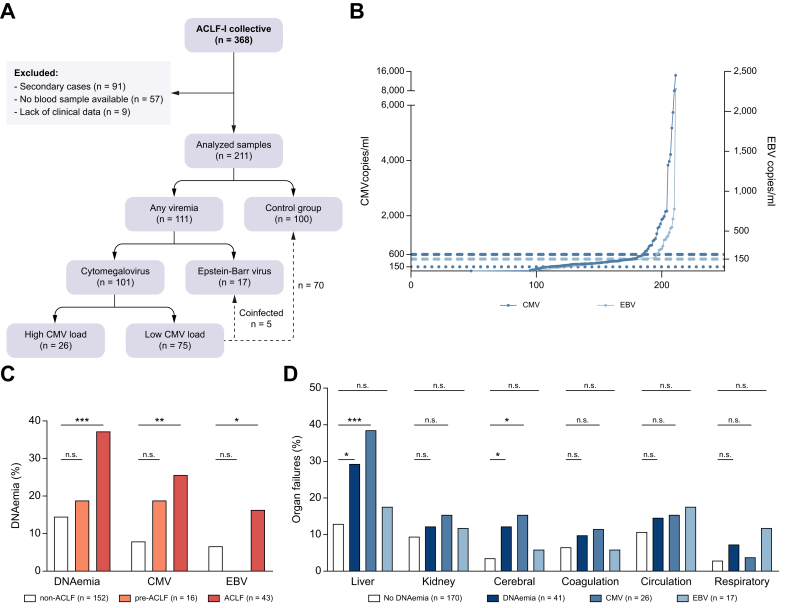
ACLF-I patient collective, DNAemia, and organ failures. (A) Flow chart of ACLF-I patient selection. High CMV load >600 copies/ml, low CMV load 150–600 copies/ml. (B) Copies/ml of CMV and EBV, cases ranked ordinally. Dotted line represents the initial CMV 150 copies/ml cut-off, dashed lines represent cut-offs for samples considered as DNAemia (CMV 600 copies/ml; EBV 150 copies/ml). (C) Distribution of DNAemia in the ACLF-I collective (Pearson‘s Chi-square test: n.s., *p* >0.05, ∗*p* <0.05, ∗∗*p* <0.01, ∗∗∗*p* <0.001). (D) Organ failures in the ACLF-I collective according to the CLIF-C ACLF score (Pearson‘s Chi-square test: n.s., *p* >0.05, ∗*p* <0.05, ∗∗*p* <0.01, ∗∗∗*p* <0.001).

With a threshold of 150 copies/ml, CMV was detected in ACLF and non-ACLF in a high percentage of patients (ACLF 48.8% *vs.* Non-ACLF 46.6%, *p* = 0.617). However, because the clinical relevance of CMV infection/reactivation correlates with viral load,^24^ and to account for the high assay sensitivity, we further categorized the patients into groups with a higher and a lower DNA load (Fig. 1A,B). For further analysis, the group with >600 copies/ml was considered to have CMV DNAemia, while a lower DNA load was considered to be not clinically relevant (Fig. 1A).^25^^,^^26^ Regarding EBV DNAemia, the initial threshold of 150 copies/ml resulted in a lower rate of detection than in CMV and a significant correlation with ACLF, and, therefore, was left unchanged.

Thereby, we identified 41 patients with CMV and/or EBV DNAemia within the ACLF-I collective (19.4%). 26 cases of CMV DNAemia (12.3%) and 17 cases of EBV DNAemia (8.1%) were identified with two patients presenting with both CMV and EBV. 16 cases of DNAemia could be attributed to the group of ACLF patients (37.2% *vs.* non-ACLF 14.5%; odds ratio [OR] 3.39; 95% CI, 1.60–7.18; *p* <0.001), respectively eleven cases of CMV (25.6% *vs.* non-ACLF 7.9%; OR 3.51; 95% CI, 1.47–8.34; *p* <0.01), and seven cases of EBV (16.3% *vs.* non-ACLF 6.6%; OR 3.01; 95% CI, 1.10–8.62; *p* <0.05). Two patients presented with CMV and EBV. In the pre-ACLF group, three patients tested positive for CMV (18.8% *vs.* non-ACLF 7.9%, *p* = 0.42) with no case of EBV (0% *vs.* non-ACLF 6.6%, *p =* 0.22) (Fig. 1C; all statistical analyses are detailed in Tables S2 and S3).

### Serological prevalence of antibodies

**Fig. 2:**
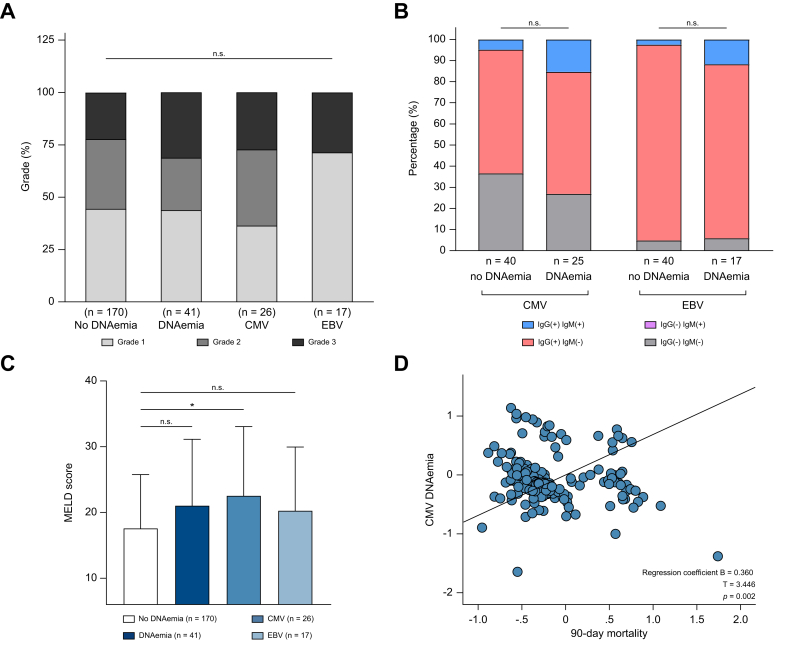
ACLF grade, serological data, MELD score, and mortality in the ACLF-I collective. (A) ACLF grades in the ACLF-I collective (individual tests Pearson‘s chi-squared test, overarching test biserial correlation using Spearman-Rho coefficients: ^n.s.^*p* >0.05, ∗*p* <0.05, ∗∗∗*p* <0.001) (B) Prevalence of CMV and EBV antibodies in a case-control group of patients with DNAemia matched with non DNAemic patients. (C) MELD score in points (average, SD) in the ACLF-I cohort (Biserial rank-correlation: ^n.s.^*p* >0.05, ∗*p* <0.05). (D) Partial regression diagram of the independent variable 90-day mortality on the CMV DNAemia group in the ACLF-I collective, including the fitting line with suppressed intersection (Multiple linear regression, backward elimination method, *p* = 0.002).

In order to discriminate between primary infection and reactivation, serological analysis was performed to detect CMV and EBV-specific IgG and IgM. All patients with DNAemia and/or ACLF and matching controls were included. Presence of IgG for CMV (DNAemia 65% *vs.* no DNAemia 62.5%, *p* = 0.82) and EBV (EBNA-1 IgG DNAemia 95% *vs.* no DNAemia 95%, *p* = 1.00) did not differ significantly (Fig. 2B). CMV IgM were more frequent in the group with DNAemia yet not reaching significance (12.5% *vs.* 5%, *p* = 0.235), while EBV-VCA IgM were borderline significantly more frequent in those patients defined as DNAemic in Pearson Chi-square test (15.0% *vs.* 2.5%, *p* = 0.048); this could not be reproduced in the logistic regression model (OR 6.88; 95% CI, 0.79–60.06; *p* = 0.08). These data indicate that reactivation rather than primary infection occurred in the vast majority of cases defined as DNAemic. Interestingly, CMV IgG prevalence was significantly higher in the ACLF group compared to non-ACLF group (80.0% *vs.* 55.0%; OR 3.27; 95% CI, 1.03–10.45; *p* <0.05).

### Influence of sex on EBV

In the presented cohort, a significant difference in EBV DNAemia between male and female patients was found. EBV DNA was detected in sera from 16.7% of females compared to 6.0% from male patients (*p* <0.05). This correlation was confirmed in the EBV regression analysis with sex as independent influence factor. Within the CMV group no association with sex was found..

### CMV is associated with liver failure and EBV with mechanical ventilation

CMV DNAemia was significantly associated with liver failure (bilirubin >12 mg/dl, *p* <0.01) and cerebral failure (West Haven Criteria grade 3 or 4, *p* <0.05) (Fig. 1D). The observed association with liver failure was additionally supported by a correlation with increased bilirubin, AST, alkaline phosphatase, and gamma-GT levels in the CMV group. EBV showed no significant correlation with a singular organ failure on univariate analysis. Yet, need for vasopressors for circulation failure and mechanical ventilation increased the risk for EBV DNAemia more than seven times. Consequently, the EBV regression analyses showed a significant independent influence for mechanical ventilation (no DNAemia 7.3% *vs.* DNAemia 33.3%).

Use of vasopressors (*p* <0.01) and mechanical ventilation (*p* <0.05) increased the risk for EBV DNAemia in the ACLF-I cohort. Aditionally, regression showed significantly reduced DNAemia in patients with gastrointestinal hemorrhage (hemorrhage 11.4% *vs.* no hemorrhage 21.6%). The ACLF grade was not significantly impacted by the presence of DNAemia (Fig. 2A). Patients testing positive for CMV displayed higher CLIF-C OF (mean 7.3 *vs.* 8.4, respectively; *p* <0.05) and CLIF-C AD (mean 63.4 *vs.* 76.6, respectively; *p* <0.05) scores as expression of the the acute disease severity. Additionally, the MELD score was significantly higher in patients with CMV (mean 17.6 *vs.* 22.6, respectively; *p* <0.05) (Fig. 2C).

### Mortality

Mortality was included in the regression models due to the clinical relevance, although it did not significantly correlate with CMV and/or EBV DNAemia in univariate analysis. It was found that 28- and 90-day mortality was significantly associated with CMV DNAemia in separate models (Fig. 2D). For EBV, a significant correlation for 90-day mortality was found in the regression analyses, while 28-day mortality was included in the respective model but did not reach significance.

### Inflammation increases in DNAemia

**Fig. 3:**
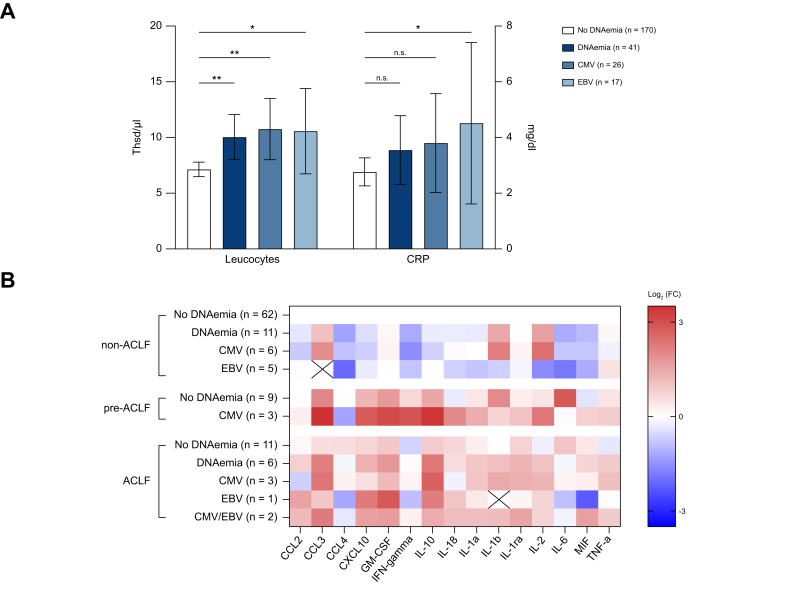
Inflammation in the ACLF-I collective. (A) Mean serum leucocyte numbers and CRP levels (bars represent SD) in the ACLF-I collective (Mann-Whitney-U-test: ^n.s.^*p* >0.05, ∗*p* <0.05, ∗∗*p* <0.01). (B) Log_2_-transformed fold changes of cytokine levels. Results of statistical analyses can be found in Table S6. CCL, CC-chemokine ligand; CXCL, C-X-C motif chemokine ligand; GM-CSF, granulocyte-macrophage colony-stimulating factor; IFN, interferon; MIF, macrophage migration inhibitory factor; TNF-α, tumor necrosis factor α.

Within both the CMV and EBV DNAemia groups, higher leukocyte levels as markers of inflammation correlated with increased risk for DNAemia (CMV U = 1,440; z = -2.83; *p* <0.01; EBV U = 980; z = -2.16; *p* <0.05) (Fig. 3A). They gained additional significance as independent influencing factors in regression analysis for EBV. In parallel, there were higher levels of CRP in patients with EBV compared with those without (U = 757; z = -2.01; *p* <0.05). Furthermore, in the CMV group, CRP gained borderline significance in the regression models as an independent predictor (*p* <0.05).

Further exploring possible mechanisms leading to the above-described clinical findings, multiplex cytokine assays of patient blood samples were performed. A subpopulation of 102 patients of the ACLF-I collective was analysed for cytokine profiles, due to sample availability (Fig. 3B). Stratification for ACLF and DNAemia of this subgroup showed overall reduced cytokine levels in non-ACLF patients when presenting relevant CMV and/or EBV DNAemia. This switches towards an increase in cytokine levels in pre-ACLF patients and ACLF patients when additionally presenting DNAemia. Furthermore, the type-I interferon (IFN) inducible chemokine C-X-C motif chemokine ligand (CXCL)-10 was significantly upregulated in patients with CMV and/or EBV DNAemia (U = 48; z = -2.98; *p* <0.01). Yet, within ACLF patients with DNAemia, levels of IFN-γ and CC-chemokine ligand (CCL4) remained unchanged. Furthermore, patients with CMV DNAemia showed higher levels of IL-10 in pre-ACLF (*p* = 0.15) and ACLF (U = 17; z = -2.39; *p* <0.05) (Table S5).

### External validation

A cohort of 153 patients admitted to the University hospitals in Aachen, Jena, and Bonn with acute decompensation of cirrhosis with and without ACLF was included to validate the results. Further focus for this cohort was laid on the inclusion of pre-ACLF patients, asonly a relatively small group with this trajectory was included in the ACLF-I. Details on patient characteristics can be found in Table 2 (for statistical analysis of the two individual cohorts, see Tables S6 and S7).

**Table 2:** Patient characteristics of the validation cohort.

Characteristic	No DNAemia	CMV and/or EBV DNAemia	*p* value
**N**	**114**	**39**	–
Age, years, mean ± SD	60.6 ± 9.9	60.3 ± 10.8	0.435
Female sex, n (%)	41 (36.0)	8 (20.5)	0.074

**Acute-on-chronic liver failure, n (%)**			
ACLF	12 (10.5)	27 (69.2)	<0.001
Pre-ACLF	27 (23.7)	6 (15.4)	0.089
Liver failure	1 (0.9)	3 (7.7)	0.021
Renal failure	12 (10.5)	27 (69.2)	<0.001
Cerebral failure	6 (5.3)	0 (0)	0.144

**Etiology of cirrhosis, n (%)**			
Alcohol	92 (80.7)	27 (69.2)	0.137
MASH	7 (6.1)	2 (5.1)	0.817
Cholestatic liver disease	3 (2.6)	1 (2.6)	0.982
HCV	3 (2.6)	1 (2.6)	0.982
HBV	1 (0.9)	2 (5.1)	0.098
Autoimmune hepatitis	1 (0.9)	0 (0)	0.557
Other etiologies	7 (6.1)	6 (15.4)	0.074

**Laboratory values, median (IQR)**			
Creatinine, mg/L	1.07 (0.71)	2.55 (2.82)	<0.001
Bilirubin, mg/dl	1.28 (1.30)	1.52 (2.40)	0.418
AST, U/L	57 (53)	79.5 (80)	0.031
ALT, U/L	33 (34)	44 (44)	0.301
INR	1.3 (0.2)	1.4 (0.5)	0.042
Albumin, g/dl	2.9 (0.8)	2.7 (0.9)	0.612
CRP, mg/L	1.62 (1.56)	2.56 (2.68)	0.065
White blood cell count, x 10^9^/L	6.84 (4.52)	6.95 (5.60)	0.943

**Disease severity scores, median (IQR)**			
MELD	12 (6)	22 (13)	<0.001
CLIF-C ACLF	48 (6)	44 (11)	0.320
CLIF-C AD	52 (8)	51 (14)	0.960
CLIF-C OF	6 (0)	7 (2)	<0.001

**Clinical data, n (%)**			
Diabetes mellitus	33 (28.9)	19 (48.7)	0.024
COPD	11 (9.6)	1 (2.6)	0.155
Heart failure	14 (12.3)	5 (12.8)	0.930
Arterial hypertension	33 (28.9)	19 (48.7)	0.024
Coronary artery disease	11 (9.6)	4 (10.3)	0.912
Chronic kidney disease	16 (14.0)	8 (20.5)	0.337
HCC	6 (5.3)	3 (7.7)	0.578
Immunosuppression∗	1 (0.9)	0 (0)	0.557
In patients with pre-ACLF	1 (0.9)	0 (0)	0.632

**Outcome, n (%)**			
28-day mortality	19 (16.7)	6 (15.4)	0.852
90-day mortality	31 (27.2)	10 (25.6)	0.850

The no DNAemia and CMV and/or EBV DNAemia subgroups of patients at the time of inclusion, organ failure definitions according to EASL CLIF-C (Pearson‘s Chi-square test, biserial rank-correlation, Mann-Whitney *U* test or *t* test depending on variable level). ∗Immunosuppressive regime with mycophenolic acid and everolimus due to heart transplantation. ACLF, acute-on-chronic liver failure; AD, acute decompensation; ALT, alanine aminotransferase; AST, aspartate aminotransferase; CLIF-C, Chronic Liver Failure Consortium; CMV, cytomegalovirus; COPD, chronic obstructive pulmonary disease; CRP, C-reactive protein; EBV, Epstein–Barr virus; HCC, hepatocellular carcinoma; INR, international normalized ratio; MASH, metabolic-associated steatohepatitis; MELD, model of end-stage liver disease; MELDNa, model of end-stage liver disease including sodium; OF, organ failure.

### Prevalence of DNAemia in pre-ACLF and ACLF

**Fig. 4:**
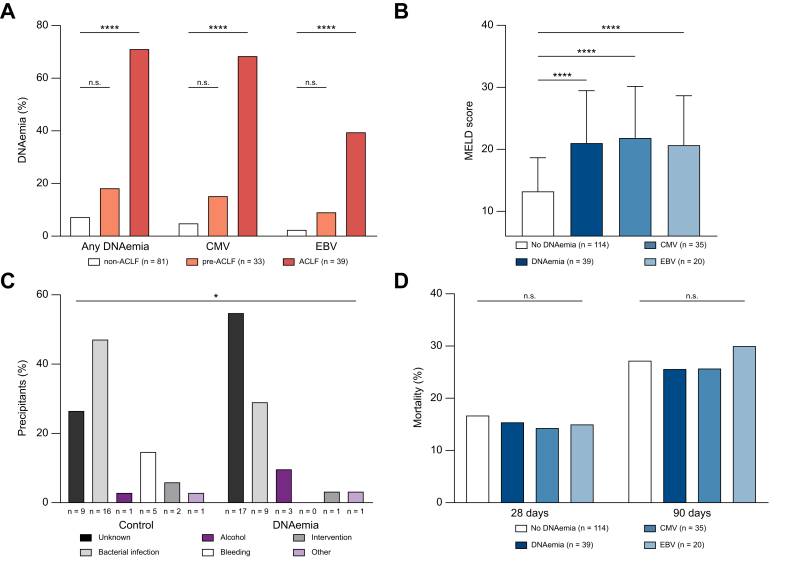
DNAemia, precipitants of ACLF and severity of disease in the validation cohort. (A) Distribution of CMV and EBV DNAemia over the validation cohort (Pearson‘s chi-squared test: ^n.s.^*p* >0.05, ∗∗∗∗*p* <0.0001). (B) MELD score in points (average, SD) in the validation cohort (Biserial rank-correlation: ∗∗∗∗*p* <0.0001). (C) ACLF precipitants in the validation cohort (Pearson‘s chi-squared test: ∗*p* <0.05). (D) Mortality in the validation cohort (Pearson‘s chi-squared test: ^n.s.^*p* >0.05).

We found 39 cases of DNAemia within the validation cohort (25.5%), with 35 cases of CMV DNAemia (22.9%) and 20 cases of EBV DNAemia (13.1%) identified (Table S4). Respectively, 16 patients were presenting with CMV and EBV. 26 cases of CMV (66.7% *vs.* non-ACLF 4.9%; OR 40.6; 95% CI, 12.0–137.1; *p* <0.00001) and 15 cases of EBV (38.5% *vs.* non-ACLF 2.5%; OR 46.9; 95% CI, 9.5–231.3; *p* <0.00001) could be attributed to the ACLF group. In the pre-ACLF group, three cases of EBV DNAemia (9.1%; OR 4.0; 95% CI, 0.6–25.1; *p* = 0.11) and five cases of CMV DNAemia (15.2%; OR 3.5; 95% CI, 0.9–13.9; *p* = 0.06) were identified (Fig. 4A) (see Tables S2 and S4 for all statistical analyses). Serological results revealed a similar picture to the ACLF-I cohort, showing no significant differences in IgG (CMV IgG DNAemia 66.7% *vs.* no DNAemia 64.3%; EBV EBNA-1 IgG DNAemia 66.7% *vs.* no DNAemia 100%) and CMV IgM (DNAemia 16.7% *vs.* no DNAemia 0%) (data not shown).

### Disease severity and DNAemia

Congruently to the ACLF-I cohort, DNAemia correlated strongly with MELD score (CMV r = 0.679; *p* <0.0001; EBV r = 0.699; *p* <0.0001; Fig. 4B). No impact on the ACLF grade was identified. Furthermore, a higher CLIF-C ACLF score (r = 0.922; *p* <0.01) correlated with EBV DNAemia. A direct comparison between the cohorts revealed lower ACLF grades in the validation cohort compared to the ACLF-I (*p* <0.0001).

Within the validation cohort, correlations could be identified for DNAemia with ACLF and especially renal failure, with every ACLF patient in this cohort presenting this organ failure. Consequently, higher creatinine similarly showed a strong correlation (*p* <0.00001) and was included in the regression models as a significant influence factor. Congruent results were found in the subgroups of CMV and EBV. In parallel to ACLF-I, CMV DNAemia showed a significant correlation with liver failure (OR 10.6; 95% CI, 1.1–105.3; *p* <0.05). In addition, increased INR correlated with CMV (U = 1,480.5; z = -2.33; *p* <0.05).

### Inflammation and mortality in DNAemia

In this cohort, no significant association of CRP and leukocytes with CMV and/or EBV DNAemia could be found. Immunosuppressive therapy showed no significant correlation with the presence of DNAemia, as in the ACLF-I cohort (see Table S9 for sensitivity analyses).

Sex did not have a significant impact on CMV and/or EBV DNAemia or CMV DNAemia alone in the validation cohort. Yet, male patients were affected by EBV DNAemia significantly more frequently (17.3% *vs.* females 4.1%, *p* <0.05). Sex did gain significance in the regression models for CMV.

In parallel to the ACLF-I cohort, no significant difference in mortality of the groups could be identified on univariable analysis (Fig. 4D). Regression analysis yielded models including 28-day and 90-day mortality as significant influence factors for CMV in separate models.

### ACLF precipitants and DNAemia

For the validation cohort documentation of ACLF precipitants was available (Fig. 4C). Analysis showed significant differences between the groups with and without DNAemia (*p* <0.05) as bacterial infections dominated in the no DNAemia group as most frequent precipitant (47.1% *vs.* DNAemia 29.0%, *p* = 0.14), while most patients within the DNAemia group had an unknown precipitant (54.8% *vs.* no DNAemia 26.5%; OR 3.37; 95% CI, 1.19–9.54; *p* <0.05).

## Discussion

ACLF is a severe clinical syndrome, being associated with mortality rates of up to 80%.^5^ Although infection and reactivation of CMV and EBV are frequent in immune dysfunction, a correlation with ACLF has so far not been investigated.

The first important finding of the present study is the presence of CMV and EBV DNAemia in significantly higher frequency in ACLF patients, which could be validated in the validation cohort. Reactivation in patients who are critically ill is frequent and can occur in 6 and up to 55% of patients in intensive care units.^27^^,^^28^ Therefore, our identified rates of DNAemia of 19.4% and 25.5% are therefore in line with prior publications regarding critically ill patients. The serological analysis of immunoglobulins suggests that, with IgG present in 65% of CMV and 100% of EBV cases, reactivations of previous infections occurred as expected when considering the high seroprevalence of these viruses in adult populations. Whilefew patients with CMV DNAemia did not express any immunoglobulins, this may be caused by CAID and does not necessarily infer primary infection. The difference in CMV IgG between ACLF and non-ACLF, albeit interesting, may be unspecific as IgM showed no such difference.

Yet, clinically relevant HHV infection is considered an issue attributed mainly to immunosuppression, neoplasia, and critically ill patients and reactivation is debated wheter to impact outcomes in intensive care unit cohorts.^29^^,^^30^ Therefore, diagnostics and treatment of HHV reactivation is currently no standard procedure in intensive care units.^31^ In the presented cohorts, DNAemia correlated with possible consequences of infections, such as liver failure for CMV, mechanical ventilation and the need for vasopressors for EBV.^28^ The associations with organ failures and mortality may reflect more advanced disease and immune dysfunction, as the single time point analysed does not allow to infer causality.

Besides, as inflammation is gaining increasing attention as an important factor in ACLF,^32^ we found significant correlations between CMV and EBV prevalence and inflammatory markers, such as leucocytes and CRP, in the ACLF-I cohort. For the immunosuppressive therapies present in the cohorts (*e.g.* because of autoimmune hepatitis), no correlation with DNAemia was found and sensitivity analyses revealed no contradicting results (Tables S8 and S9). Further cytokine analysis showed overall reduced cytokine levels in non-ACLF but with DNAemia. This footprint switched toward an increased cytokine expression in pre-ACLF and ACLF patients, in line with previously described characteristics of inflammation in ACLF.^12^ However, IFN-γ levels remained relatively low to unchanged in the ACLF DNAemia groups. Detection of low IFN-γ levels is in accordance with a recent study describing decreasing levels in patients with acutely decompensated cirrhosis as associated with progression of organ failures and posing a risk factor for the development of ACLF.^33^ Furthermore, IFN-γ is reduced in patients affected by immunosuppressive therapies and CAID.^34^^,^^35^ IFN-stimulated genes in peripheral blood mononuclear cells of patients with alcoholic cirrhosis are also known to show decreased constitutive expression and lower induction.^36^ Therefore, patients with lower IFN-γ levels may be at risk of developing CMV/EBV DNAemia.^37^ Since we did not observe a correlation between DNAemia and immunosuppressive therapies, the relatively reduced levels in ACLF patients maypredispose this group for CMV/EBV DNAemia. Our findings further included increased IL-10 levels in CMV and EBV positive patients with pre-ACLF and ACLF, which is part of the viral immunoevasive mechanisms.^38^ The increase in IL-10 may be of clinical importance due to weakening effects on the immune system. The elevated levels of the IFN-induced chemokine CXCL10 in patients with CMV and/or EBV DNAemia indicate a virally driven surge in type-I IFN production. In the context of cirrhosis, type-I IFNs act on myeloid cells, thereby priming subsequent inflammatory responses and enhancing susceptibility to inflammatory caspase activation and inflammasome assembly, driving inflammation and organ failure in cirrhosis.^39^ Therefore, the cytokine data may support a potential role of CMV/EBV as a precipitant or sustainer of ACLF, depending on the time of DNAemia development. However, longitudinal data are needed to further clarify the viral contribution to these nonspecific markers and distinguish the findings from the systemic inflammation intrinsic to ACLF.

Recently, associations with mortality in patients critically ill with COVID-19 have been published, supporting our findings of mortality being associated with CMV/EBV DNAemia.^29^ However, although it has been frequently reported that HHV reactivation can alter patients’ prognoses, a causal link remains to be proven.

Therefore, we also investigated patients with pre-ACLF and precipitating events to determine whether CMV and/or EBV DNAemia is a cause or consequence of ACLF. Given that only a few patients in the ACLF-I cohort presented with pre-ACLF, a validation cohort from Bonn, including 26 pre-ACLF 49 non-ACLF, was incorporated in this study. In the past, few studies have evaluated the impact of CMV and/or EBV DNAemia on ACLF and liver function, especially in terms of the possible triggering effect.^40^^,^^41^ Given that use of non-standardized diagnostic criteria for ACLF and lack of validation, the results were not transferable. In this study, we used criteria established by the EASL-CLIF-C for the diagnosis of ACLF.

In all cohorts and in nearly every subgroup, the frequency of DNAemia increased steadily from non-ACLF over pre-ACLF to ACLF, although not reaching significance in patients with pre-ACLF in the ACLF-I and validation cohorts (Fig. 1, Fig. 4A). Further analysis of the documented ACLF precipitants identified a gap of known triggers in the validation DNAemia groups. Given that CMV and EBV infections are known triggers for systemic inflammation and can cause cytokine production and persistent immune cell activation, they might act as extrahepatic precipitants for the development of ACLF.^42^ Intrahepatic insults are also possible in the form of CMV hepatitis, as observed in this study, given that CMV was associated with liver failure.^43^ In addition, as a known precipitant of ACLF, gastrointestinal hemorrhage was associated with a lower risk for DNAemia. However, future studies are required to investigate this trend further.

In the ACLF-I cohort, serological and/or PCR screening for CMV and EBV infection/reactivation during treatment was performed in only 6% of patients and no case of retrospectively identified CMV and/or EBV DNAemia was identified throughout the treatment period. No clinical signs of CMV or EBV infections were documented. Trials conducted of treatment of CMV reactivation in patients who were critically ill have thus far reported negative results.[44], [45], [46] However, these results are not transferable to ACLF collectives and the situation might be different in patients with ACLF, given that CMV and EBV infection/replication might act as the underlying precipitant of ACLF. A hallmark and sustainer of ACLF is continuous inflammation, presenting an additional characteristic not similarly relevant in patients in general intensive care units.^10^^,^^47^ Furthermore, the prospective identification of patients with pre-ACLF being investigated in studies such as MICROB-PREDICT could offer treatment opportunities not available for other syndromes. However, the definitive identification of the clinical significance of CMV and EBV reactivation for outcomes, screening, and identification of patients at high risk of developing unfavorable clinical courses and, therefore, in need of prompt treatment, should be evaluated in comparable further studies.

Taken together, our data describe a correlation between DNAemia and ACLF, suggesting DNAemia as prognostic marker for ACLF. However, because of the retrospective nature of our study, no definitive causal relationship between DNAemia and ACLF can be established. Accordingly, prospective analyses of patient material and correlations with the clinical situation is needed in further studies.

Another limitation of our study is that, because of the availability of samples in this retrospective analysis of prospective trials, blood serum samples were used for analysis. Aside from higher sensitivity for detection of CMV, previous studies have shown that CMV DNA can be up to 10-fold higher in whole blood compared with serum samples, likely because of the detection of intracellular viruses.^48^^,^^49^ In addition, different commercial reagents and laboratory-developed tests have shown up to a 3 log_10_ difference in CMV viral load detection.^50^ Therefore, the above-mentioned LLOD and thresholds regarding considerations of results as relevant DNAemia can not be directly translated into clinically used international units. Furthermore, the threshold for CMV copies was derived from previous publications on transplant recipients and has not been prospectively validated in non-solid organ transplant recipients.^25^^,^^26^ Thus, this might underestimate the prevalence and viral load, which could affect classifications and associations.

A strength of this study is the external validation, which was conducted with patient cohorts recruited in two different German research projects. Aside from the external validation and increased heterogeneity, the burden of disease was distributed differently from the ACLF-I cohort, presenting significantly lower ACLF grades compared to the ACLF-I cohort. With lower ACLF grades, these patients might exhibit less immune dysfunction than found in critically ill cohorts and the impact of CAID might also be lower. The differences in terms of organ failures between the cohorts can be explained by the aetiologies of ACLF, with the ACLF-I cohort presenting higher frequencies of liver failure and the Aachen/Jena cohort exclusively presenting ACLFs including renal failure. Therefore, the validation cohort provides valuable insights, given that renal failure ranks among the most frequent organ failures found in ACLF and was underrepresented in the ACLF-I cohort (27.9%).^5^

## Conclusions

Our data show that CMV and EBV DNAemia is associated with ACLF, the severity of disease, the underlying stage of liver cirrhosis, mortality, and a distinct pattern of inflammation. Reactivation/infection with these viruses could contribute to development/aggravation of the syndrome by exacerbating liver inflammation and impairing hepatocellular function. Our data also suggest that CMV and EBV DNAemia act as potentially treatable precipitants of ACLF. However, further studies are needed to evaluate the role of these viruses and antiviral treatments in the context of ACLF.

## Abbreviations

ACLF, acute-on-chronic liver failure; AD, acute decompensation; ALT, alanine aminotransferase; AST, aspartate aminotransferase; CAID, cirrhosis-associated immune dysfunction; CCL, CC-chemokine ligand; CLIF-C, Chronic Liver Failure Consortium; CMV, cytomegalovirus; COPD, chronic obstructive pulmonary disease; CRP, C-reactive protein; Ct, cycle threshold; CV, coefficient of variation; CXCL, C-X-C motif chemokine ligand; EBV, Epstein–Barr virus; GM-CSF, granulocyte-macrophage colony-stimulating factor; HCC, hepatocellular carcinoma; HHV, human herpesvirus; IFN, interferon; INR, international normalized ratio; LLOD, lower limit of detection; MASH, metabolic-associated steatohepatitis; MELD, model of end-stage liver disease; MELDNa, model of end-stage liver disease including sodium; MIF, macrophage migration inhibitory factor; OF, organ failure; OR, odds ratio; PhHV, Phocine herpesvirus; qPCR, real-time quantitative PCR; TNF-α, tumor necrosis factor α.

## Authors’ contributions

Study concept and design: KT, JS, EH, KHP. Data acquisition: KT, JS, EG, FEU, MJB, WG, RS, SK, MSM, MMM, TW, PAR, JR, FS, NB, NK, MP, PT, JF, SZ, CW, JT, AS, JC, TB, KHP. Analysis and interpretation of data: KT, JS, PL, MG, NK, EH, KHP. Drafting of manuscript, statistical analysis: JS. Critical revision of manuscript for important intellectual content: JS, KT, AS, JT, JC, TB, EH, KHP. Obtained funding: JS, SZ, CW, SC, JT, TB, EH, KHP. Administrative, technical, or material support: JT, JC, TB, EH, KHP. Study supervision: CW, JT, EH, KHP. All authors approved the final version of the article, including the authorship list.

## Data availability

The datasets generated and/or analyzed during the study are not publicly available because of ongoing expansion and analysis, but are available from the corresponding author on reasonable request.

## Financial support

This work was funded by the State Hesse, Germany (‘Landes-Offensive zur Entwicklung Wissenschaftlich-ökonomischer Exzellenz’, ACLF-I, project P7), the 10.13039/501100001659Deutsche Forschungsgemeinschaft (10.13039/501100001659DFG, German Research Foundation; 493624047), Clinician Scientist Careers Münster (322274963), SFB1382 Project ID 403224013/B07, Clinician Scientist Program Münster No. 2024_002, and the Advanced Clinician Scientist Program (ACCENT, funding code 01EO2107), sponsored by the German 10.13039/501100002347Federal Ministry of Education and Research (10.13039/501100002347BMBF).

## Conflict of interest

The authors declare no conflicts of interest that pertain to this work.

Please refer to the accompanying ICMJE disclosure forms for further details.
