# Supplementary material for: Higher prevalence of cytomegalovirus and Epstein–Barr virus in acute-on-chronic liver failure
Source: JHEP Rep. 2025 Oct 9;8(4):101627. doi: 10.1016/j.jhepr.2025.101627 (PMC13019566; doi:10.1016/j.jhepr.2025.101627)
Supplement: Multimedia component 1 [file mmc1.pdf]

Higher prevalence of Cytomegalovirus and Epstein–Barr virus in acute-on-chronic liver failure

Keerthihan Thiyagarajah, Jannik Sonnenberg (shared first)**,** Esra Görgülü, Pia Lembeck, Nico Kraus, Mirco Glitscher, Frank Erhard Uschner Maximilian Joseph Brol, Wenyi Gu, Robert Schierwagen, Sabine Klein, Martin S. McCoy, Markus Maximilian Mücke, Toska Wiedemann, Philipp A. Reuken, Johanna Reißing, Franziska Schneider, Nina Böhling, Michael Praktiknjo, I-Robin Tepasse, Julia Fischer, Stefan Zeuzem, Christoph Welsch, Sandra Ciesek, Andreas Stallmach, Jonel Trebicka, Johannes Chang, Tony Bruns, Eberhard Hildt, Kai-Henrik Peiffer

Table of contents

Supplementary analyses 2

Supplementary individual validation cohorts analyses 20

Supplementary sensitivity analyses 29

Supplementary methods 38

Supplementary references 45

Supplementary analyses

Table S1. Multiplex-qPCR master mix. Abbreviations: CN = Catalogue Number, ddH2O = double-distilled water

Ingredients	1x reaction
LightCycler Multiplex DNA Master (CN 07339577001)	4 µl
CMV probe (CN 08997837001)	0.5 µl
EBV probe (CN 10097710001)	0.5 µl
PhHV probe (CN 07093802001)	0.5 µl
ddH2O	2.5 µl

Table S2. Multiple regression analyses models and results of the ACLF-I and validation cohort. Linear regression models using the backwards method. Abbreviations: TIPS = Transjugular intrahepatic portosystemic stent, Regr. Coeff. = Regression coefficient, SE = Standard error, T = t-value

	**ACLF-I**				
**CMV DNAemia**					
**Variables included**	**90-day mortality**, age, sex, aetiology of cirrhosis, Diabetes, Arterial hypertension, Coronary artery disease, Bacterial infections, Vasopressors, TIPS, beta blocker treatment, Supplemental oxygen including mechanical ventilation, West Haven grade of Hepatic encephalopathy, albumin, bilirubin, leucocytes, C-reactive protein, Gamma-glutamyltransferase, alkaline phosphatase				
	**Results:**	Regr. Coeff.	SE	T	*p*
	90-day mortality	0.39	0.10	3.81	0.00
	Etiology of cirrhosis	-0.02	0.01	-2.17	0.04
	Arterial hypertension	-0.38	0.13	-3.00	0.01
	Age	0.01	0.01	2.05	0.05
	C-reactive protein	0.04	0.02	2.08	0.05
	Gamma-glutamyltransferase	0.00	0.00	2.83	0.01
	Corrected R^2 = 0.575	F=8.652	*p*=0.000023		
**Variables included**	**28-day mortality**, age, sex, aetiology of cirrhosis, Diabetes, Arterial hypertension, Coronary artery disease, Bacterial infections, Vasopressors, TIPS, beta blocker treatment, Supplemental oxygen including mechanical ventilation, West Haven grade of Hepatic encephalopathy, albumin, bilirubin, leucocytes, C-reactive protein, Gamma-glutamyltransferase, alkaline phosphatase				
	**Results:**	Regr. Coeff.	SE	T	*p*
	28-day mortality	0.25	0.12	2.05	0.05
	Arterial hypertension	-0.30	0.14	-2.22	0.03
	C-reactive protein	0.04	0.02	1.99	0.06
	Gamma-glutamyltransferase	0.00	0.00	1.90	0.07
	Corrected R^2 = 0.426	F=7.321	*p*=0.00031		

EBV DNAemia

**Variables included**	**90-day mortality**, sex, aetiology of cirrhosis, Diabetes, Arterial hypertension, Ascites, Gastrointestinal bleeding, Bacterial infections, Vasopressors, Dialysis, TIPS, Immunosuppression, Antibiotic prophylaxis, Mechanical ventilation, albumin, leucocytes, C-reactive protein, Alanine transaminase				
	**Results:**	Regr. Coeff.	SE	T	*p*
	Sex	-0.09	0.04	-2.09	0.04
	90-day mortality	-0.14	0.05	-2.63	0.01
	Ascites	0.08	0.04	2.06	0.04
	TIPS	-0.11	0.05	-2.05	0.04
	Mechanical ventilation	0.28	0.12	2.38	0.02
	Leukocytes	0.02	0.00	3.93	0.00
	Corrected R^2 = 0.172	F=6.441	p=0.0000046		
**Variables included**	**28-day mortality**, sex, aetiology of cirrhosis, Diabetes, Arterial hypertension, Ascites, Gastrointestinal bleeding, Bacterial infections, Vasopressors, Dialysis, TIPS, Immunosuppression, Antibiotic prophylaxis, Mechanical ventilation, albumin, leucocytes, C-reactive protein, Alanine transaminase				
	**Results:**	Regr. Coeff.	SE	T	*p*
	28-day mortality	-0.14	0.07	-1.93	0.06
	Arterial hypertension	-0.08	0.04	-1.83	0.07
	Ascites	0.07	0.04	1.66	0.10
	Antibiotic prophylaxis	-0.10	0.05	-1.99	0.05
	Mechanical ventilation	0.28	0.12	2.35	0.02
	Leukocytes	0.02	0.00	3.78	0.00
	Corrected R^2 = 0.165	F=6.153	p=0.0000086		

CMV and/or EBV DNAemia

**Variables included**	**90-day mortality**, age, sex, aetiology of cirrhosis, Cerebral failure, Diabetes, Arterial hypertension, Coronary artery disease, Chronic renal failure, Ascites, Gastrointestinal bleeding, Bacterial infections, Vasopressors, TIPS, Immunosuppression, beta blocker treatment, Supplemental oxygen including mechanical ventilation, Albumin, bilirubin, Leukocytes, C-reactive protein, Aspartate aminotransferase, alkaline phosphatase, Gamma-glutamyltransferase				
	**Results:**	Regr. Coeff.	SE	T	*p*
	Arterial hypertension	-0.21	0.08	-2.62	0.01
	Gastrointestinal bleeding	-0.20	0.08	-2.54	0.01
	Vasopressors	0.60	0.21	2.83	0.01
	Bilirubin	0.01	0.00	1.90	0.06
	Leukocytes	0.01	0.01	1.71	0.09
	Aspartate aminotransferase	0.00	0.00	-1.84	0.07
	Corrected R^2 = 0.230	F=6.737	p=0.0000043		
**Variables included**	**28-day mortality**, age, sex, aetiology of cirrhosis, Cerebral failure, Diabetes, Arterial hypertension, Coronary artery disease, Chronic renal failure, Ascites, Gastrointestinal bleeding, Bacterial infections, Vasopressors, TIPS, Immunosuppression, beta blocker treatment, Supplemental oxygen including mechanical ventilation, Albumin, bilirubin, Leukocytes, C-reactive protein, Aspartate aminotransferase, alkaline phosphatase, Gamma-glutamyltransferase				
	**Results:**	Regr. Coeff.	SE	T	*p*

	Arterial hypertension	-0.21	0.08	-2.62	0.01
	Gastrointestinal bleeding	-0.20	0.08	-2.54	0.01
	Vasopressors	0.60	0.21	2.83	0.01
	Bilirubin	0.01	0.00	1.90	0.06
	Leukocytes	0.01	0.01	1.71	0.09
	Aspartate aminotransferase	0.00	0.00	-1.84	0.07
	Corrected R^2 = 0.230	F=6.737	*p*=0.0000043		

Validation cohort

CMV DNAemia

**Variables included**	**90-day mortality**, sex, aetiology of cirrhosis, ACLF precipitant, cerebral failure, liver failure, age, albumin, sodium, creatinine, International normalized ratio, C-reactive protein, Aspartate aminotransferase, platelets				
	**Results:**	Regr. Coeff.	SE	T	*p*
	90-day mortality	-0.27	0.12	-2.31	0.03
	Sex	-0.28	0.11	-2.44	0.02
	Cerebral failure	-0.85	0.26	-3.28	0.00
	ACLF precipitant	0.09	0.04	2.19	0.03
	Albumin	-0.01	0.01	-1.70	0.10
	Sodium	-0.02	0.01	-1.71	0.09
	Creatinine	0.17	0.04	4.65	0.00
	Corrected R^2 = 0.459	F=7.677	*p*=0.0000033		
**Variables included**	**28-day mortality**, sex, aetiology of cirrhosis, ACLF precipitant, cerebral failure, liver failure, age, albumin, sodium, creatinine, International normalized ratio, C-reactive protein, Aspartate aminotransferase, platelets				
	**Results:**	Regr. Coeff.	SE	T	*p*
	28-day mortality	-0.27	0.13	-2.13	0.04
	Sex	-0.24	0.11	-2.10	0.04
	Cerebral failure	-0.70	0.23	-3.00	0.00
	Sodium	-0.02	0.01	-2.57	0.01
	Creatinine	0.13	0.04	3.72	0.00
	Corrected R^2 = 0.425	F=9.116	*p*=0.0000033		

EBV DNAemia

**Variables included**	**90-day mortality**, sex, aetiology of cirrhosis, ACLF precipitant, cerebral failure, age, creatinine, C-reactive protein, Aspartate aminotransferase, Alanine transaminase, platelets				
	**Results:**	Regr. Coeff.	SE	T	*p*
	90-day mortality	-0.19	0.11	-1.74	0.09
	Cerebral failure	-0.62	0.21	-2.96	0.01
	Age	-0.01	0.01	-2.37	0.02
	Creatinine	0.16	0.04	4.42	0.00
	Aspartate aminotransferase	0.00	0.00	2.57	0.01

	Platelets	0.00	0.00	-1.76	0.09
	Corrected R^2 = 0.507	F=8.712	*p*=0.0000049		
**Variables included**	**28-day mortality**, sex, aetiology of cirrhosis, ACLF precipitant, cerebral failure, age, creatinine, C-reactive protein, Aspartate aminotransferase, Alanine transaminase, platelets				
	**Results:**	Regr. Coeff.	SE	T	*p*
	Cerebral failure	-0.63	0.22	-2.91	0.01
	Age	-0.02	0.01	-2.74	0.01
	Creatinine	0.19	0.04	5.13	0.00
	Aspartate aminotransferase	0.00	0.00	1.90	0.06
	Corrected R^2 = 0.460	F=10.577	*p*=0.0000056		

CMV and/or EBV DNAemia

**Variables included**	**90-day mortality**, age, sex, aetiology of cirrhosis, ACLF precipitant, cerebral failure, liver failure, Renal failure, International normalized ratio, platelets				
	**Results:**	Regr. Coeff.	SE	T	*p*
	90-day mortality	-0.24	0.11	-2.10	0.04
	Sex	-0.32	0.11	-2.81	0.01
	Cerebral failure	-0.97	0.25	-3.86	0.00
	ACLF precipitant	0.10	0.04	2.48	0.02
	Albumin	-0.02	0.01	-2.28	0.03
	Creatinine	0.18	0.04	4.82	0.00
	Corrected R^2 = 0.445	F=8.471	*p*=0.0000023		
**Variables included**	**28-day mortality**, age, sex, aetiology of cirrhosis, ACLF precipitant, Cerebral failure, Liver failure, Renal failure, International normalized ratio, platelets				
	**Results:**	Regr. Coeff.	SE	T	*p*
	28-day mortality	-0.28	0.13	-2.20	0.03
	Sex	-0.25	0.11	-2.22	0.03
	Cerebral failure	-0.72	0.23	-3.07	0.00
	Sodium mmol/l	-0.02	0.01	-2.47	0.02
	Creatinine mg/dl	0.13	0.04	3.66	0.00
	Corrected R^2 = 0.424	F=7.814	*p*=0.00015		

Table S3. ACLF-I analyses. Analyses separated by statistical method. Abbreviations: COPD = Chronic obstructive pulmonary disease, TIPS = Transjugular intrahepatic portosystemic stent, MELD = Model for End-Stage Liver Disease score, CLIF-C = Chronic Liver Failure Consortium, AD = Acute decompensation, OF = Organ failure; [1]calculation of Odds ratio not possible due to small patient groups; [2]calculation of Odds ratio due to table >2x2 not possible

Tests vs. CMV DNAemia

Pearson-Chi-Square test

**Variable**	***p***	**Odds ratio**	**Lower 95% CI**	**Higher 95% CI**
ACLF	0.00	3.51	1.47	8.34
Pre-ACLF	0.42	1.73	0.46	6.52
Sex	0.10	0.49	0.21	1.15

28-day mortality	0.10	2.46	0.81	7.47
90-day mortality	0.38	1.53	0.59	3.94
Liver failure	0.00	4.20	1.70	10.43
Renal failure	0.35	1.75	0.54	5.71
Cerebral failure	0.01	4.97	1.30	19.00
Coagulation failure	0.35	1.89	0.49	7.27
Circulation failure	0.48	1.53	0.47	4.92
Respiratory failure	0.80	1.32	0.15	11.77
Diabetes	0.29	0.59	0.23	1.56
COPD	0.95	0.93	0.11	7.89
Heart failure	0.33 [1]			
Hypertension	0.01	0.19	0.04	0.83
Coronary artery disease	0.11 [1]			
Chronic kidney disease	0.38 [1]			
Ascites	0.53	1.32	0.56	3.14
Hepatic encephalopathy	0.10	2.03	0.85	4.83
Gastrointestinal bleeding	0.39	0.61	0.20	1.88
Bacterial infections	0.30	1.55	0.68	3.55
Viral infections	0.63	1.71	0.18	15.93
Vasopressors	0.14	3.46	0.60	19.91
Dialysis	0.49 [1]			
TIPS	0.20	0.39	0.09	1.73
Immunosuppression	0.89	1.12	0.24	5.31
Beta blockers	0.01	0.28	0.10	0.80
Antibiotic prophylaxis	0.59	1.32	0.48	3.63
Oxygen supplementation	0.08	2.64	0.86	8.12
Mechanical ventilation	0.11	2.61	0.76	8.96
Any oxygen supplement	0.48	2.23	0.22	22.25
Aetiology of cirrhosis	0.21 [2]			

Biserial rank-correlation

**Variable**	***p***	**Corr. Coeff.**	**DF**
ACLF grade	0.65	0.08	38
West Haven	0.07	0.24	196
MELD	0.04	0.45	188
MELD sodium	0.09	0.51	188
CLIF-C ACLF	0.09	0.49	195
CLIF-C AD	0.02	0.64	195
CLIF-C OF	0.01	0.35	196
Child-Pugh score	0.09	0.20	196
Child-Pugh category	0.07	0.13	196

Shapiro-Wilk test

**Variables**	***p***	**W**
Age	0.18	0.98
Sodium	0.03	0.98
Creatinine	0.00	0.73
Bilirubin	0.00	0.73
Albumin	0.21	0.99
International normalized ratio	0.00	0.78
Leukocytes	0.00	0.88
C-reactive protein	0.00	0.77
Aspartate aminotransferase	0.00	0.63
Alanine aminotransferase	0.00	0.65
Alkaline phosphatase	0.00	0.66
Gamma-glutamyltransferase	0.00	0.27
Platelets	0.00	0.84

T-test

**Variables**	***p***	**DF**	**T**	**Cohen's d**
Age	0.07	209	-1.432	-0.25
Albumin	0.21	207	-1.005	-0.18

Mann-Whitney-U test

**Variables**	***p***	**U**	**Z**	**r**

Sodium	0.56	2055.00	-0.58	0.04
Creatinine	0.79	2139.00	-0.27	0.02
Bilirubin	0.03	1635.00	-2.14	0.15
International normalized ratio	0.95	2193.00	-0.06	0.00
Leukocytes	0.00	1440.00	-2.83	0.20
C-reactive protein	0.16	1578.00	-1.41	0.10
Aspartate aminotransferase	0.01	926.00	-2.44	0.20
Alanine aminotransferase	0.21	1741.00	-1.26	0.09
Alkaline phosphatase	0.01	869.00	-2.77	0.22
Gamma-glutamyltransferase	0.01	1484.00	-2.52	0.18
Platelets	0.43	1627.00	-0.66	0.05

Tests vs. EBV DNAemia

**Pearson-Chi-Square test**				
**Variable**	***p***	**Odds ratio**	**Lower 95% CI**	**Higher 95% CI**
ACLF	0.03	3.01	1.10	8.62
Pre-ACLF	0.22	[1]		
Sex	0.02	0.32	0.12	0.88
28-day mortality	0.68	0.65	0.08	5.21
90-day mortality	0.44	0.55	0.12	2.54
Liver failure	0.59	1.44	0.38	5.42
Renal failure	0.75	1.28	0.27	6.12
Cerebral failure	0.63	1.71	0.19	15.09
Coagulation failure	0.92	0.90	0.11	7.46
Circulation failure	0.38	1.80	0.47	6.86
Respiratory failure	0.07	4.40	0.79	24.64
Diabetes	0.18	0.42	0.12	1.54
COPD	0.39	[1]		
Heart failure	0.63	1.71	0.19	15.09
Hypertension	0.10	0.30	0.07	1.37
Coronary artery disease	0.68	0.65	0.08	5.21
Chronic kidney disease	0.47	[1]		
Ascites	0.06	3.27	0.90	11.79
Hepatic encephalopathy	0.28	1.77	0.62	5.10
Gastrointestinal bleeding	0.29	0.45	0.10	2.04
Bacterial infections	0.09	2.44	0.86	6.89
Viral infections	0.53	[1]		
Vasopressors	0.00	8.89	1.81	43.75
Dialysis	0.26	3.48	0.34	35.42
TIPS	0.21	0.29	0.04	2.28
Immunosuppression	0.19	2.46	0.62	9.66
Beta blockers	0.31	0.58	0.20	1.67
Antibiotic prophylaxis	0.14	0.24	0.03	1.84
Oxygen supplementation	0.04	3.52	1.00	12.39
Mechanical ventilation	0.40	1.96	0.39	9.74
Any oxygen supplement	0.01	7.42	1.15	47.94
Aetiology of cirrhosis	0.93	[2]		
**Biserial rank-correlation**				
**Variable**	***p***	**Corr. Coeff.**	**DF**	
ACLF grade	0.45	0.31	34	
West Haven	0.56	0.14	186	
MELD	0.33	0.35	182	
MELD sodium	0.22	0.38	182	
CLIF-C ACLF	0.10	0.43	185	
CLIF-C AD	0.38	0.66	185	
CLIF-C OF	0.43	0.23	186	
Child-Pugh score	0.49	0.34	186	
Child-Pugh category	0.14	0.09	186	
**Shapiro-Wilk test**				
**Variables**	***p***	**W**		

Age	0.47	0.99		
Sodium	0.04	0.97		
Creatinine	0.00	0.79		
Bilirubin	0.00	0.72		
Albumin	0.27	0.99		
International normalized ratio	0.00	0.77		
Leukocytes	0.00	0.87		
C-reactive protein	0.00	0.77		
Aspartate aminotransferase	0.00	0.63		
Alanine aminotransferase	0.00	0.63		
Alkaline phosphatase	0.00	0.66		
Gamma-glutamyltransferase	0.00	0.28		
Platelets	0.00	0.91		
**T-test**				
**Variables**	***p***	**DF**	**T**	**Cohen's d**
Age	0.38	185	-0.30	-0.08
Albumin	0.09	26.02	-1.40	-0.24
**Mann-Whitney-U test**				
**Variables**	***p***	**U**	**Z**	**r**
Sodium	0.36	1252.00	-0.91	0.07
Creatinine	0.72	1369.00	-0.36	0.03
Bilirubin	0.59	1330.50	-0.54	0.04
International normalized ratio	0.67	1354.50	-0.43	0.03
Leukocytes	0.03	980.00	-2.16	0.16
C-reactive protein	0.04	756.50	-2.01	0.15
Aspartate aminotransferase	0.82	719.50	-0.23	0.02
Alanine aminotransferase	0.03	818.00	-2.17	0.16
Alkaline phosphatase	0.87	960.00	-0.17	0.01
Gamma-glutamyltransferase	0.84	1198.50	-0.20	0.02
Platelets	0.53	916.50	-0.71	0.06

Tests vs. CMV and/or EBV DNAemia

**Pearson-Chi-Square test**<br>**Variable**	***p***	**Odds ratio**	**Lower 95% CI**	**Higher 95% CI**
ACLF	0.00	3.39	1.60	7.18
Pre-ACLF	0.94	0.95	0.26	3.51
Sex	0.01	0.42	0.21	0.84
28-day mortality	0.26	1.77	0.64	4.89
90-day mortality	0.99	1.01	0.43	2.38
Liver failure	0.01	2.78	1.24	6.25
Renal failure	0.59	1.34	0.46	3.89
Cerebral failure	0.03	3.80	1.10	13.12
Coagulation failure	0.46	1.56	0.47	5.18
Circulation failure	0.47	1.44	0.53	3.89
Respiratory failure	0.19	2.61	0.60	11.38
Diabetes	0.15	0.56	0.25	1.25
COPD	0.61	0.58	0.07	4.87
Heart failure	0.73	0.68	0.08	5.84
Hypertension	0.01	0.25	0.08	0.72
Coronary artery disease	0.17	0.26	0.03	2.01
Chronic kidney disease	0.27	[1]		
Ascites	0.16	1.69	0.81	3.54
Hepatic encephalopathy	0.16	1.69	0.81	3.52
Gastrointestinal bleeding	0.13	0.47	0.17	1.27
Bacterial infections	0.13	1.70	0.85	3.38
Viral infections	0.95	1.08	0.12	9.93
Vasopressors	0.03	4.49	1.07	18.77
Dialysis	0.78	1.39	0.14	13.73
TIPS	0.10	0.37	0.11	1.27
Immunosuppression	0.30	1.77	0.59	5.30

Beta blockers	0.03	0.42	0.20	0.92
Antibiotic prophylaxis	0.73	0.85	0.34	2.10
Oxygen supplementation	0.03	2.73	1.05	7.06
Mechanical ventilation	0.21	2.02	0.66	6.19
Any oxygen supplement	0.05	4.39	0.85	22.62
Aetiology of cirrhosis	0.39	[2]		
**Biserial rank-correlation**				
**Variable**	***p***	**Corr. Coeff.**	**DF**	
ACLF grade	0.75	0.11	43	
West Haven	0.12	0.19	211	
MELD	0.09	0.40	203	
MELD sodium	0.11	0.45	203	
CLIF-C ACLF	0.06	0.43	210	
CLIF-C AD	0.07	0.59	210	
CLIF-C OF	0.06	0.28	211	
Child-Pugh score	0.15	0.26	211	
Child-Pugh category	0.05	0.14	211	
**Shapiro-Wilk test**				
**Variables**	***p***	**W**		
Age	0.38	0.99		
Sodium	0.04	0.98		
Creatinine	0.00	0.74		
Bilirubin	0.00	0.73		
Albumin	0.15	0.99		
International normalized ratio	0.00	0.79		
Leukocytes	0.00	0.89		
C-reactive protein	0.00	0.79		
Aspartate aminotransferase	0.00	0.62		
Alanine aminotransferase	0.00	0.64		
Alkaline phosphatase	0.00	0.66		
Gamma-glutamyltransferase	0.00	0.27		
Platelets	0.00	0.85		
**T-test**				
**Variables**	***p***	**DF**	**T**	**Cohen's d**
Age	0.08	209	-1.43	-0.59
Albumin	0.16	207	-1.01	-0.52
**Mann-Whitney-U test**				
**Variables**	***p***	**U**	**Z**	**r**
Sodium	0.56	3283.00	-0.58	0.04
Creatinine	1.00	3484.00	0.00	0.00
Bilirubin	0.11	2932.00	-1.58	0.11
International normalized ratio	0.77	3382.00	-0.29	0.02
Leukocytes	0.00	2417.00	-3.00	0.21
C-reactive protein	0.07	2318.00	-1.83	0.13
Aspartate aminotransferase	0.07	1579.00	-1.84	0.15
Alanine aminotransferase	0.79	3049.00	-0.26	0.02
Alkaline phosphatase	0.06	1780.00	-1.86	0.14
Gamma-glutamyltransferase	0.05	2555.00	-2.00	0.14
Platelets	0.32	2585.00	-0.28	0.02

Table S4. Validation cohort analyses. Analyses separated by statistical method. Abbreviations: MELD = Model for End-Stage Liver Disease score, CLIF-C = Chronic Liver Failure Consortium, AD = Acute decompensation, OF = Organ failure, [1]calculation of Odds ratio not possible due to small patient groups; [2]calculation of significance not possible as one variable is a constant; [3]calculation of Odds ratio due to table >2x2 not possible

Tests vs. CMV DNAemia

Pearson-Chi-Square test

**Variable**	***p***	**Odds ratio**	**Lower 95% CI**	**Higher 95% CI**
ACLF	0.00	40.63	12.04	137.10
pre-ACLF	0.07	3.47	0.87	13.89
Sex	0.15	0.53	0.22	1.27
28-day mortality	0.74	0.83	0.29	2.42
90-day mortality	0.86	0.93	0.39	2.20
Liver failure	0.01	10.59	1.07	105.34
Renal failure	0.00	24.56	9.35	64.49
Cerebral failure	0.17	[1]		
Coagulation failure	[2]			
Circulation failure	[2]			
Respiratory failure	[2]			
Immunosuppression	0.58	[3]		
Precipitant	0.34			
ACLF precipitant	0.04	[3]		

Biserial rank-correlation

**Variable**	***p***	**Corr. Coeff.**	**DF**
ACLF grade	0.62	0.25	38
West Haven	0.34	0.15	149
MELD	0.00	0.68	149
CLIF-C ACLF	0.30	0.74	38
CLIF-C AD	0.91	0.56	111
CLIF-C OF	0.00	0.64	111
Child-Pugh category	0.39	0.11	149

Shapiro-Wilk test

**Variables**	***p***	**W**
Age	0.44	0.99
Sodium	0.00	0.94
Creatinine	0.00	0.74
Bilirubin	0.00	0.39
Albumin	0.27	0.99
International normalized ratio	0.00	0.86
Leukocytes	0.00	0.89
C-reactive protein	0.00	0.74
Aspartate aminotransferase	0.00	0.44
Alanine aminotransferase	0.00	0.59
Platelets	0.00	0.90

T-test

**Variables**	***p***	**DF**	**T**	**Cohen's d**
Age	0.43	147	-0.182	-0.035
Albumin	0.29	144	-0.565	-0.124

Mann-Whitney-U test

**Variables**	***p***	**U**	**Z**	**r**
Sodium	0.18	1698	-1.33	0.11
Creatinine	0.00	655.5	-6.00	0.49
Bilirubin	0.32	1774	-0.99	0.08
International normalized ratio	0.02	1480.5	-2.33	0.19
Leukocytes	0.77	1928.5	-0.30	0.02
C-reactive protein	0.09	1612	-1.71	0.14
Aspartate aminotransferase	0.06	1243.5	-1.89	0.16
Alanine aminotransferase	0.50	1758.5	-0.67	0.06
Platelets	0.20	1707.5	-1.29	0.11

Tests vs. EBV DNAemia

Pearson-Chi-Square test

**Variable**	***p***	**Odds ratio**	**Lower 95% CI**	**Higher 95% CI**

ACLF	0.00	46.88	9.50	231.32
pre-ACLF	0.10	4.17	0.66	26.30
Sex	0.02	0.20	0.04	0.90
28-day mortality	0.85	0.88	0.24	3.31
90-day mortality	0.80	1.15	0.41	3.25
Liver failure	0.67	[1]		
Renal failure	0.00	25.50	7.87	82.63
Cerebral failure	0.29	[1]		
Coagulation failure	[2]			
Circulation failure	[2]			
Respiratory failure	[2]			
Aetiology of cirrhosis	0.67	[3]		
ACLF precipitant	0.28	[3]		
Immunosuppression	0.20	[1]		

Biserial rank-correlation

Variable	*p*	Corr. Coeff.	DF
ACLF grade	0.11	0.32	27
West Haven	0.74	0.18	134
MELD	0.00	0.70	134
CLIF-C ACLF	0.01	0.92	27
CLIF-C AD	0.62	0.47	107
CLIF-C OF	0.00	0.63	96
Child-Pugh category	0.72	0.03	134

Shapiro-Wilk test

Variables	*p*	W
Age	0.72	0.99
Sodium	0.00	0.93
Creatinine	0.00	0.74
Bilirubin	0.00	0.50
Albumin	0.45	0.99
International normalized ratio	0.00	0.87
Leukocytes	0.00	0.89
C-reactive protein	0.00	0.72
Aspartate aminotransferase	0.00	0.44
Alanine aminotransferase	0.00	0.57
Platelets	0.00	0.89

T-test

Variables	*p*	DF	T	Cohen's d
Age	0.16	132	-0.994	-0.241
Albumin	0.49	129	0.026	0.007

Mann-Whitney-U test

Variables	*p*	U	Z	r
Sodium	0.40	1005	-0.84	0.07
Creatinine	0.00	321.5	-5.11	0.44
Bilirubin	0.79	1098	-0.26	0.02
International normalized ratio	0.52	1038.5	-0.64	0.06
Leukocytes	0.90	1119.5	-0.13	0.01
C-reactive protein	0.13	895	-1.53	0.13
Aspartate aminotransferase	0.02	558	-2.26	0.21
Alanine aminotransferase	0.30	956.5	-1.04	0.09
Platelets	0.19	929	-1.32	0.11

Tests vs. CMV and/or EBV DNAemia

Pearson-Chi-Square test

Variable	*p*	Odds ratio	Lower 95% CI	Higher 95% CI
ACLF	0.00	28.13	9.61	82.34
pre-ACLF	0.09	2.78	0.83	9.35
Sex	0.07	0.46	0.19	1.09
28-day mortality	0.85	0.91	0.34	2.47
90-day mortality	0.85	0.923	0.403	2.115

Liver failure	0.02	9.42	0.95	93.37
Renal failure	0.00	19.13	7.73	47.31
Cerebral failure	0.14 [1]			
Coagulation failure	[2]			
Circulation failure	[2]			
Respiratory failure	[2]			
Aetiology of cirrhosis	0.56 [3]			
ACLF precipitant	0.33 [3]			
Immunosuppression	0.05 [1]			

Biserial rank-correlation

**Variable**	***p***	**Corr. Coeff.**	**DF**
ACLF grade	0.59	0.25	39
West Haven	0.22	0.17	153
MELD	0.00	0.65	153
CLIF-C ACLF	0.32	0.74	39
CLIF-C AD	0.96	0.52	114
CLIF-C OF	0.00	0.62	114
Child-Pugh category	0.33	0.12	153

Shapiro-Wilk test

**Variables**	***p***	**W**
Age	0.50	0.99
Sodium	0.00	0.94
Creatinine	0.00	0.74
Bilirubin	0.00	0.39
Albumin	0.30	0.99
International normalized ratio	0.00	0.87
Leukocytes	0.00	0.89
C-reactive protein	0.00	0.74
Aspartate aminotransferase	0.00	0.46
Alanine aminotransferase	0.00	0.60
Platelets	0.00	0.90

T-test

**Variables**	***p***	**DF**	**T**	**Cohen's d**
Age	0.43	147	-0.182	-0.03
Albumin	0.29	144	-0.565	-0.124

Mann-Whitney-U test

**Variables**	***p***	**U**	**Z**	**r**
Sodium	0.18	1907	-1.33	0.11
Creatinine	0.00	835	-5.81	0.47
Bilirubin	0.42	2029.5	-0.81	0.07
International normalized ratio	0.04	1742.5	-2.03	0.16
Leukocytes	0.94	2206	-0.07	0.01
C-reactive protein	0.07	1783	-1.84	0.15
Aspartate aminotransferase	0.03	1278	-2.16	0.19
Alanine aminotransferase	0.30	1888.5	-1.04	0.08
Platelets	0.25	1948.5	-1.15	0.09

Table S5. ACLF-I cytokine analyses. Abbreviations: CCL = CC-chemokine ligand; CXCL = C-X-C motif chemokine ligand; GM-CSF = Granulocyte-macrophage colony-stimulating factor; IFN-gamma = Interferon gamma; IL = Interleukin; MIF = Macrophage migration inhibitory factor; TNF-a = Tumor necrosis factor a.

Non-ACLF no DNAemia vs. non-ACLF CMV and/or EBV DNAemia

Shapiro-Wilk test

**Variables**	***p***	**W**
CCL2	0.00	0.83
CCL3	0.00	0.42
CCL4	0.00	0.20

CXCL10	0.00	0.33
GM-CSF	0.00	0.29
IFN-gamma	0.00	0.40
IL-10	0.00	0.35
IL-18	0.00	0.49
IL-1a	0.00	0.68
IL-1b	0.00	0.42
IL-1ra	0.00	0.84
IL-2	0.00	0.25
IL-6	0.00	0.43
MIF	0.00	0.65
TNF-a	0.00	0.89

**Mann-Whitney-U test**<br>**Variables**	***p***	**U**	**Z**	**r**
CCL2	0.84	323	-0.20	0.02
CCL3	0.71	323	-0.38	0.04
CCL4	0.60	308	-0.52	0.06
CXCL10	0.47	295	-0.72	0.08
GM-CSF	0.23	264	-1.21	0.14
IFN-gamma	0.75	321	-0.32	0.04
IL-10	0.95	337	-0.06	0.01
IL-18	0.85	329	-0.19	0.02
IL-1a	0.64	311	-0.46	0.05
IL-1b	0.31	280	-1.01	0.12
IL-1ra	0.85	329	-0.19	0.02
IL-2	0.71	320	-0.38	0.04
IL-6	0.67	314	-0.42	0.05
MIF	0.59	307	-0.53	0.06
TNF-a	0.52	299	-0.65	0.08

Non-ACLF no DNAemia vs. non-ACLF CMV DNAemia

**Shapiro-Wilk test**<br>**Variables**	***p***	**W**
CCL2	0.00	0.82
CCL3	0.00	0.44
CCL4	0.00	0.21
CXCL10	0.00	0.32
GM-CSF	0.00	0.29
IFN-gamma	0.00	0.40
IL-10	0.00	0.34
IL-18	0.00	0.50
IL-1a	0.00	0.69
IL-1b	0.00	0.43
IL-1ra	0.00	0.86
IL-2	0.00	0.26
IL-6	0.00	0.44
MIF	0.00	0.66
TNF-a	0.00	0.91

**Mann-Whitney-U test**<br>**Variables**	***p***	**U**	**Z**	**r**
CCL2	0.43	147	-0.79	0.10
CCL3	0.55	165	-0.60	0.07
CCL4	0.43	150	-0.78	0.09
CXCL10	0.31	140	-1.01	0.12
GM-CSF	0.73	171	-0.34	0.04
IFN-gamma	0.36	145	-0.91	0.11
IL-10	0.84	177	-0.21	0.02
IL-18	0.47	153	-0.72	0.09
IL-1a	0.85	177	-0.19	0.02
IL-1b	0.24	135	-1.19	0.14

IL-1ra	0.40	147	-0.84	0.10
IL-2	0.60	165	-0.53	0.06
IL-6	0.56	159	-0.58	0.07
MIF	0.99	186	-0.01	0.00
TNF-a	0.73	170	-0.35	0.04

Non-ACLF no DNAemia vs. non-ACLF EBV DNAemia

**Shapiro-Wilk test**				
**Variables**	***p***	**W**		
CCL2	0.00	0.84		
CCL3	0.00	0.47		
CCL4	0.00	0.20		
CXCL10	0.00	0.32		
GM-CSF	0.00	0.27		
IFN-gamma	0.00	0.41		
IL-10	0.00	0.35		
IL-18	0.00	0.48		
IL-1a	0.00	0.66		
IL-1b	0.00	0.62		
IL-1ra	0.00	0.83		
IL-2	0.00	0.22		
IL-6	0.00	0.43		
MIF	0.00	0.66		
TNF-a	0.00	0.89		
**Mann-Whitney-U test**				
**Variables**	***p***	**U**	**Z**	**r**
CCL2	0.57	129	-0.57	0.07
CCL3	0.20	115	-1.28	0.16
CCL4	0.95	153	-0.06	0.01
CXCL10	1.00	155	0.00	0.00
GM-CSF	0.13	93	-1.50	0.18
IFN-gamma	0.61	134	-0.51	0.06
IL-10	0.90	150	-0.13	0.02
IL-18	0.62	134	-0.50	0.06
IL-1a	0.35	116	-0.93	0.11
IL-1b	0.80	145	-0.26	0.03
IL-1ra	0.22	104	-1.22	0.15
IL-2	1.00	155	0.00	0.00
IL-6	0.19	101	-1.30	0.16
MIF	0.42	121	-0.81	0.10
TNF-a	0.17	97	-1.38	0.17

Non-ACLF no DNAemia vs. pre-ACLF no DNAemia

**Shapiro-Wilk test**		
**Variables**	***p***	**W**
CCL2	0.00	0.81
CCL3	0.00	0.39
CCL4	0.00	0.23
CXCL10	0.00	0.36
GM-CSF	0.00	0.32
IFN-gamma	0.00	0.45
IL-10	0.00	0.36
IL-18	0.00	0.50
IL-1a	0.00	0.71
IL-1b	0.00	0.49
IL-1ra	0.00	0.83
IL-2	0.00	0.25
IL-6	0.00	0.18
MIF	0.00	0.67
TNF-a	0.00	0.93

Mann-Whitney-U test				
**Variables**	***p***	**U**	**Z**	**r**
CCL2	0.95	271	-0.06	0.01
CCL3	0.10	202	-1.65	0.20
CCL4	0.05	167	-1.95	0.23
CXCL10	0.47	238	-0.72	0.09
GM-CSF	0.02	145	-2.35	0.28
IFN-gamma	0.02	143	-2.37	0.28
IL-10	0.02	141	-2.39	0.28
IL-18	0.82	266	-0.23	0.03
IL-1a	0.12	189	-1.57	0.19
IL-1b	0.04	167	-2.05	0.24
IL-1ra	0.41	231	-0.83	0.10
IL-2	0.02	160	-2.29	0.27
IL-6	0.01	136	-2.47	0.29
MIF	0.77	262	-0.29	0.03
TNF-a	0.03	153	-2.18	0.26

Non-ACLF no DNAemia vs. pre-ACLF CMV DNAemia

Shapiro-Wilk test				
**Variables**	***p***	**W**		
CCL2	0.00	0.83		
CCL3	0.00	0.45		
CCL4	0.00	0.21		
CXCL10	0.00	0.33		
GM-CSF	0.00	0.30		
IFN-gamma	0.00	0.37		
IL-10	0.00	0.28		
IL-18	0.00	0.45		
IL-1a	0.00	0.64		
IL-1b	0.00	0.63		
IL-1ra	0.00	0.82		
IL-2	0.00	0.28		
IL-6	0.00	0.46		
MIF	0.00	0.70		
TNF-a	0.00	0.88		
**Mann-Whitney-U test**				
**Variables**	***p***	**U**	**Z**	**r**
CCL2	0.95	90	-0.06	0.01
CCL3	0.00	11	-3.19	0.40
CCL4	0.48	71	-0.71	0.09
CXCL10	0.23	55	-1.19	0.15
GM-CSF	0.07	35	-1.84	0.23
IFN-gamma	0.34	63	-0.95	0.12
IL-10	0.15	48	-1.42	0.18
IL-18	0.49	71	-0.69	0.09
IL-1a	0.08	38	-1.72	0.21
IL-1b	0.87	88	-0.17	0.02
IL-1ra	0.05	32	-1.92	0.24
IL-2	0.15	53	-1.44	0.18
IL-6	0.24	56	-1.17	0.15
MIF	0.26	57	-1.13	0.14
TNF-a	0.12	43	-1.56	0.19

Non-ACLF no DNAemia vs. ACLF no DNAemia

Shapiro-Wilk test		
**Variables**	***p***	**W**
CCL2	0.00	0.86
CCL3	0.00	0.55

CCL4	0.00	0.24
CXCL10	0.00	0.40
GM-CSF	0.00	0.34
IFN-gamma	0.00	0.40
IL-10	0.00	0.42
IL-18	0.00	0.52
IL-1a	0.00	0.71
IL-1b	0.00	0.65
IL-1ra	0.00	0.79
IL-2	0.00	0.23
IL-6	0.00	0.57
MIF	0.00	0.71
TNF-a	0.00	0.91

**Mann-Whitney-U test**				
**Variables**	***p***	**U**	**Z**	**r**
CCL2	0.99	335	-0.01	0.00
CCL3	0.04	232	-2.05	0.24
CCL4	0.08	227	-1.76	0.21
CXCL10	0.06	220	-1.87	0.22
GM-CSF	0.01	164	-2.76	0.32
IFN-gamma	0.18	255	-1.34	0.16
IL-10	0.01	177	-2.54	0.30
IL-18	0.01	174	-2.58	0.30
IL-1a	0.07	225	-1.79	0.21
IL-1b	0.31	279	-1.02	0.12
IL-1ra	0.04	206	-2.08	0.24
IL-2	0.02	203	-2.36	0.28
IL-6	0.00	151	-2.94	0.34
MIF	0.28	271	-1.09	0.13
TNF-a	0.88	331	-0.15	0.02

Non-ACLF no DNAemia vs. ACLF CMV and/or EBV DNAemia

**Shapiro-Wilk test**		
**Variables**	***p***	**W**
CCL2	0.00	0.83
CCL3	0.00	0.52
CCL4	0.00	0.21
CXCL10	0.00	0.40
GM-CSF	0.00	0.34
IFN-gamma	0.00	0.41
IL-10	0.00	0.41
IL-18	0.00	0.53
IL-1a	0.00	0.70
IL-1b	0.00	0.63
IL-1ra	0.00	0.75
IL-2	0.00	0.24
IL-6	0.00	0.46
MIF	0.00	0.70
TNF-a	0.00	0.92

**Mann-Whitney-U test**				
**Variables**	***p***	**U**	**Z**	**r**
CCL2	0.22	102	-1.22	0.15
CCL3	0.03	103	-2.23	0.27
CCL4	0.12	115	-1.55	0.19
CXCL10	0.00	48	-2.98	0.36
GM-CSF	0.00	47	-3.04	0.37
IFN-gamma	0.17	123	-1.38	0.17
IL-10	0.01	58	-2.78	0.34
IL-18	0.14	119	-1.46	0.18
IL-1a	0.01	71	-2.49	0.30

IL-1b	0.37	148	-0.89	0.11
IL-1ra	0.09	107	-1.72	0.21
IL-2	0.01	79	-2.59	0.31
IL-6	0.12	89	-1.58	0.19
MIF	0.40	147	-0.84	0.10
TNF-a	0.03	84	-2.22	0.27

Non-ACLF no DNAemia vs. ACLF CMV DNAemia

**Shapiro-Wilk test**				
**Variables**	***p***	**W**		
CCL2	0.00	0.83		
CCL3	0.00	0.50		
CCL4	0.00	0.21		
CXCL10	0.00	0.32		
GM-CSF	0.00	0.27		
IFN-gamma	0.00	0.41		
IL-10	0.00	0.35		
IL-18	0.00	0.49		
IL-1a	0.00	0.68		
IL-1b	0.00	0.61		
IL-1ra	0.00	0.86		
IL-2	0.00	0.23		
IL-6	0.00	0.46		
MIF	0.00	0.68		
TNF-a	0.00	0.92		
**Mann-Whitney-U test**				
**Variables**	***p***	**U**	**Z**	**r**
CCL2	0.61	48	-0.51	0.06
CCL3	0.10	51	-1.67	0.21
CCL4	0.68	80	-0.41	0.05
CXCL10	0.03	23	-2.19	0.27
GM-CSF	0.02	17	-2.41	0.30
IFN-gamma	0.41	67	-0.82	0.10
IL-10	0.02	17	-2.39	0.30
IL-18	0.77	84	-0.30	0.04
IL-1a	0.08	37	-1.75	0.22
IL-1b	0.47	72	-0.72	0.09
IL-1ra	0.48	71	-0.70	0.09
IL-2	0.16	54	-1.40	0.17
IL-6	0.12	22	-1.54	0.19
MIF	0.85	87	-0.19	0.02
TNF-a	0.17	50	-1.36	0.17

Non-ACLF no DNAemia vs. ACLF EBV DNAemia

**Shapiro-Wilk test**		
**Variables**	***p***	**W**
CCL2	0.00	0.84
CCL3	0.00	0.51
CCL4	0.00	0.21
CXCL10	0.00	0.36
GM-CSF	0.00	0.31
IFN-gamma	0.00	0.41
IL-10	0.00	0.38
IL-18	0.00	0.51
IL-1a	0.00	0.68
IL-1b	0.00	0.61
IL-1ra	0.00	0.86
IL-2	0.00	0.23
IL-6	0.00	0.45

MIF	0.00	0.67		
TNF-a	0.00	0.92		
**Mann-Whitney-U test**				
**Variables**	***p***	**U**	**Z**	**r**
CCL2	0.12	3	-1.54	0.20
CCL3	0.18	12	-1.34	0.17
CCL4	0.23	10	-1.19	0.15
CXCL10	0.11	2	-1.59	0.20
GM-CSF	0.11	2	-1.62	0.20
IFN-gamma	0.74	25	-0.33	0.04
IL-10	0.10	1	-1.65	0.21
IL-18	0.17	6	-1.37	0.17
IL-1a	0.31	13	-1.02	0.13
IL-1b	0.34	15	-0.95	0.12
IL-1ra	0.41	16	-0.82	0.10
IL-2	0.10	5	-1.66	0.21
IL-6	0.78	26	-0.28	0.03
MIF	0.38	15	-0.88	0.11
TNF-a	0.60	22	-0.52	0.07

Non-ACLF no DNAemia vs. ACLF CMV and/or EBV DNAemia

**Shapiro-Wilk test**				
**Variables**	***p***	**W**		
CCL2	0.00	0.83		
CCL3	0.00	0.51		
CCL4	0.00	0.21		
CXCL10	0.00	0.36		
GM-CSF	0.00	0.30		
IFN-gamma	0.00	0.42		
IL-10	0.00	0.38		
IL-18	0.00	0.53		
IL-1a	0.00	0.69		
IL-1b	0.00	0.64		
IL-1ra	0.00	0.75		
IL-2	0.00	0.24		
IL-6	0.00	0.45		
MIF	0.00	0.69		
TNF-a	0.00	0.92		
**Mann-Whitney-U test**				
**Variables**	***p***	**U**	**Z**	**r**
CCL2	0.16	25	-1.41	0.18
CCL3	0.27	40	-1.09	0.14
CCL4	0.15	25	-1.43	0.18
CXCL10	0.13	23	-1.50	0.19
GM-CSF	0.18	28	-1.33	0.17
IFN-gamma	0.23	31	-1.21	0.15
IL-10	0.40	40	-0.85	0.11
IL-18	0.04	10	-2.01	0.25
IL-1a	0.12	22	-1.57	0.20
IL-1b	0.17	29	-1.36	0.17
IL-1ra	0.11	20	-1.62	0.20
IL-2	0.07	21	-1.81	0.23
IL-6	0.42	41	-0.81	0.10
MIF	0.06	13	-1.89	0.24
TNF-a	0.06	13	-1.91	0.24

Table S6. Serological analysis. Abbreviations: CMV = Cytomegalovirus, EBV = Epstein-Barr-virus, VCA = Capsid-Antigen, EBNA = Epstein-Barr nuclear antigen, IgG = Immunglobulin G, IgM = Immunglobulin M; [1]calculation of Odds ratio not possible

due to small patient groups; [2]calculation of significance not possible as one variable is a constant

ACLF-I cohort tests vs. DNAemia

Pearson-Chi-Square test Variable	*p*	Odds ratio	Lower 95% CI	Higher 95% CI
CMV IgG	0.82	1.11	0.45	2.77
CMV IgM	0.24	2.71	0.49	14.90
EBNA-1 IgG	1.00	1.00	0.13	7.47
EBV-VCA IgM	0.05	6.88	0.79	60.06

ACLF-I cohort tests vs. CMV DNAemia

Pearson-Chi-Square test Variable	*p*	Odds ratio	Lower 95% CI	Higher 95% CI
CMV IgG	0.65	1.28	0.44	3.67
CMV IgM	0.14	3.62	0.61	21.44

ACLF-I cohort tests vs. EBV DNAemia

Pearson-Chi-Square test Variable	*p*	Odds ratio	Lower 95% CI	Higher 95% CI
EBNA-1 IgG	0.89	0.84	0.07	9.96
EBV-VCA IgM	0.15	5.20	0.44	61.68

ACLF-I cohort tests in the respective subgroups vs. ACLF

Pearson-Chi-Square test Variable	*p*	Odds ratio	Lower 95% CI	Higher 95% CI
CMV IgG	0.04	3.27	1.03	10.45
CMV IgM	0.54	1.68	0.31	9.07
EBNA-1 IgG	0.16	[1]		
EBV-VCA IgM	0.85	[1]		

Validation cohort tests vs. DNAemia

Pearson-Chi-Square test Variable	*p*	Odds ratio	Lower 95% CI	Higher 95% CI
CMV IgG	0.92	1.111	0.148	8.367
CMV IgM	0.12	[1]		
EBNA-1 IgG	0.02	[1]		
EBV-VCA IgM	[2]			

Validation cohort tests vs. CMV DNAemia

Pearson-Chi-Square test Variable	*p*	Odds ratio	Lower 95% CI	Higher 95% CI
CMV IgG	0.74	0.71	0.10	5.12
CMV IgM	0.25	[1]		

Validation cohort tests vs. EBV DNAemia

Pearson-Chi-Square test Variable	*p*	Odds ratio	Lower 95% CI	Higher 95% CI
EBNA-1 IgG	0.10	[1]		
EBV-VCA IgM	[2]			

Supplementary individual validation cohorts analyses

Table S7. Validation cohort from Aachen/Jena. Analyses separated by statistical method, linear regression models using the backwards method. Abbreviations: MELD = Model for End-Stage Liver Disease score, CLIF-C = Chronic Liver Failure Consortium, AD = Acute decompensation, OF = Organ failure, [1]calculation of Odds ratio not possible due to small patient groups; [2]calculation of significance not possible as one variable is a constant; [3]calculation of Odds ratio due to table >2x2 not possible

CMV DNAemia

**Variables included**	**90-day mortality**, age, sex, aetiology of cirrhosis, ACLF precipitant, West Haven grade of Hepatic encephalopathy, bilirubin, albumin, sodium, creatinine, International normalized ratio, platelets				
	**Results:**	Regr. Coeff.	SE	T	*p*
	90-day mortality	-0.28	0.15	-1.82	0.08
	West Haven grade of Hepatic encephalopathy	-0.18	0.08	-2.14	0.04
	International normalized ratio	0.35	0.20	1.75	0.09
	Corrected R^2 = 0.225	F=4.484	p=0.0095		
**Variables included**	**28-day mortality**, age, sex, aetiology of cirrhosis, ACLF precipitant, West Haven grade of Hepatic encephalopathy, bilirubin, albumin, sodium, creatinine, International normalized ratio, platelets				
	**Results:**	Regr. Coeff.	SE	T	*p*
	28-day mortality	-0.39	0.18	-2.17	0.04
	Bilirubin	0.02	0.01	2.38	0.02
	West Haven grade of Hepatic encephalopathy	-0.24	0.08	-3.01	0.01
	Sodium	-0.02	0.01	-1.72	0.10
	Creatinine	0.09	0.05	1.88	0.07
	Corrected R^2 = 0.331	F=4.559	p=0.003		

EBV DNAemia

**Variables included**	**90-day mortality**, age, sex, aetiology of cirrhosis, ACLF precipitant, West Haven grade of Hepatic encephalopathy, albumin, creatinine, platelets				
	**Results:**	Regr. Coeff.	SE	T	*p*
	West Haven grade of Hepatic encephalopathy	-0.17	0.08	-2.13	0.04
	Age	-0.02	0.01	-2.26	0.03
	Creatinine	0.13	0.06	2.13	0.04
	Corrected R^2 = 0.371	F=5.907	p=0.004		
**Variables included**	**28-day mortality**, age, sex, aetiology of cirrhosis, ACLF precipitant, West Haven grade of Hepatic encephalopathy, albumin, creatinine, platelets				
	**Results:**	Regr. Coeff.	SE	T	*p*
	West Haven grade of Hepatic encephalopathy	-0.17	0.08	-2.13	0.04
	Age	-0.02	0.01	-2.26	0.03
	Creatinine	0.13	0.06	2.13	0.04
	Corrected R^2 = 0.371	F=5.907	p=0.004		

CMV and/or EBV DNAemia

**Variables included**	**90-day mortality**, age, sex, aetiology of cirrhosis, ACLF precipitant, Cerebral failure, Liver failure, Renal failure, International normalized ratio, platelets				
	**Results:**	Regr. Coeff.	SE	T	*p*
	90-day mortality	-0.28	0.15	-1.88	0.07
	West Haven grade of Hepatic encephalopathy	-0.18	0.08	-2.20	0.03
	International normalized ratio	0.34	0.20	1.76	0.09
	Corrected R^2 = 0.362	F=6.245	p=0.00074		
**Variables included**	**28-day mortality**, age, sex, aetiology of cirrhosis, ACLF precipitant, Cerebral failure, Liver failure, Renal failure, International normalized ratio, platelets				
	**Results:**	Regr. Coeff.	SE	T	*p*
	**28-day mortality**	-0.55	0.16	-3.43	0.00
	Cerebral failure	-0.98	0.22	-4.42	0.00
	International normalized ratio	0.36	0.16	2.28	0.03
	Platelets	0.00	0.00	-2.08	0.05
	Corrected R^2 = 0.424	F=7.814	p=0.00015		

Tests vs. CMV DNAemia

**Pearson-Chi-Square test**<br>**Variable**	***p***	**Odds ratio**	**Lower 95% CI**	**Higher 95% CI**
ACLF	0.00	89.82	19.82	738.64
pre-ACLF	0.02	[1]		
Sex	0.43	0.65	0.22	1.93
28-day mortality	0.27	0.41	0.08	2.09
90-day mortality	0.26	0.54	0.18	1.59
Liver failure	0.09	6.00	0.59	60.79
Renal failure	0.00	89.82	19.82	738.64
Cerebral failure	0.06	[1]		
Coagulation failure	[2]			
Circulation failure	[2]			
Respiratory failure	[2]			
Aetiology of cirrhosis	0.23	[3]		
ACLF precipitant	0.19	[3]		
Immunosuppression	0.46	[1]		
**Biserial rank-correlation**<br>**Variable**	***p***	**Corr. Coeff.**	**DF**	
ACLF grade	0.62	0.25	38	
West Haven	0.02	0.34	76	
MELD	0.00	0.81	76	
CLIF-C ACLF	0.11	0.74	38	
CLIF-C AD	0.82	1.00	38	
CLIF-C OF	0.31	0.41	38	
Child-Pugh category	0.64	-0.06	76	
**Shapiro-Wilk test**<br>**Variables**	***p***	**W**		
Age	0.55	0.98		
Sodium	0.00	0.92		
Creatinine	0.00	0.85		
Bilirubin	0.00	0.49		
Albumin	0.58	0.98		
International normalized ratio	0.00	0.91		
Leukocytes	0.00	0.92		

C-reactive protein	0.00	0.83		
Aspartate aminotransferase	0.00	0.48		
Alanine aminotransferase	0.00	0.63		
Platelets	0.00	0.87		
**T-test**				
**Variables**	***p***	**DF**	**T**	**Cohen’s d**
Age	0.41	74.00	-0.23	-0.06
Albumin	0.11	35.69	1.24	0.35
**Mann-Whitney-U test**				
**Variables**	***p***	**U**	**Z**	**r**
Sodium	0.25	556.50	-1.14	0.13
Creatinine	0.00	108.00	-6.01	0.69
Bilirubin	0.82	641.00	-0.22	0.03
International normalized ratio	0.08	499.50	-1.77	0.20
Leukocytes	0.49	597.50	-0.69	0.08
C-reactive protein	0.98	659.00	-0.03	0.00
Aspartate aminotransferase	0.84	418.50	-0.20	0.03
Alanine aminotransferase	0.55	593.50	-0.60	0.07
Platelets	0.09	505.50	-1.69	0.19

Tests vs. EBV DNAemia

**Pearson-Chi-Square test**				
**Variable**	***p***	**Odds ratio**	**Lower 95% CI**	**Higher 95% CI**
ACLF	0.00	51.82	6.14	437.17
pre-ACLF	0.02 [1]			
Sex	0.15	0.32	0.07	1.61
28-day mortality	0.71	0.73	0.14	3.87
90-day mortality	0.80	0.86	0.26	2.87
Liver failure	0.57 [1]			
Renal failure	0.00	51.82	6.14	437.17
Cerebral failure	0.14 [1]			
Coagulation failure	[2]			
Circulation failure	[2]			
Respiratory failure	[2]			
Aetiology of cirrhosis	0.23 [3]			
ACLF precipitant	0.44 [3]			
Immunosuppression	0.57 [1]			
**Biserial rank-correlation**				
**Variable**	***p***	**Corr. Coeff.**	**DF**	
ACLF grade	0.11	0.32	27	
West Haven	0.02	0.40	65	
MELD	0.00	0.84	65	
CLIF-C ACLF	0.01	0.92	27	
CLIF-C AD	0.50	0.56	38	
CLIF-C OF	0.18	0.42	27	
Child-Pugh category	0.23	-0.15	65	
**Shapiro-Wilk test**				
**Variables**	***p***	**W**		
Age	0.96	0.99		
Sodium	0.00	0.89		
Creatinine	0.00	0.83		
Bilirubin	0.00	0.57		
Albumin	0.27	0.97		
International normalized ratio	0.00	0.90		
Leukocytes	0.00	0.91		
C-reactive protein	0.00	0.81		
Aspartate aminotransferase	0.00	0.51		
Alanine aminotransferase	0.00	0.62		
Platelets	0.00	0.87		
**T-test**				

Variables	*p*	DF	T	Cohen's d
Age	0.10	63.00	-1.28	-0.37
Albumin	0.08	17.62	1.46	0.54
**Mann-Whitney-U test**				
**Variables**	***p***	**U**	**Z**	**r**
Sodium	0.69	365.50	-0.41	0.05
Creatinine	0.00	68.50	-4.93	0.61
Bilirubin	0.99	391.00	-0.02	0.00
International normalized ratio	0.32	327.50	-0.99	0.12
Leukocytes	0.31	325.50	-1.01	0.13
C-reactive protein	0.85	380.00	-0.18	0.02
Aspartate aminotransferase	0.68	250.00	-0.41	0.06
Alanine aminotransferase	0.66	355.50	-0.44	0.06
Platelets	0.02	235.00	-2.39	0.30

Tests vs. CMV and/or EBV DNAemia

**Pearson-Chi-Square test**				
**Variable**	***p***	**Odds ratio**	**Lower 95% CI**	**Higher 95% CI**
ACLF	0.00	46.64	9.56	227.63
pre-ACLF	0.00	[1]		
Sex	0.34	0.59	0.20	1.75
28-day mortality	0.46	0.59	0.14	2.43
90-day mortality	0.33	0.60	0.21	1.69
Liver failure	0.11	5.54	0.55	55.96
Renal failure	0.00	46.64	9.56	227.63
Cerebral failure	0.05	[1]		
Coagulation failure	[2]			
Circulation failure	[2]			
Respiratory failure	[2]			
Aetiology of cirrhosis	0.23	[3]		
ACLF precipitant	0.23	[3]		
Immunosuppression	0.44	[1]		
**Biserial rank-correlation**				
**Variable**	***p***	**Corr. Coeff.**	**DF**	
ACLF grade	0.59	0.25	39	
West Haven	0.03	0.35	78	
MELD	0.00	0.80	78	
CLIF-C ACLF	0.08	0.74	39	
CLIF-C AD	0.75	0.81	39	
CLIF-C OF	0.25	0.42	39	
Child-Pugh category	0.82	-0.03	78	
**Shapiro-Wilk test**				
**Variables**	***p***	**W**		
Age	0.67	0.99		
Sodium	0.00	0.92		
Creatinine	0.00	0.84		
Bilirubin	0.00	0.48		
Albumin	0.54	0.98		
International normalized ratio	0.00	0.91		
Leukocytes	0.00	0.92		
C-reactive protein	0.00	0.83		
Aspartate aminotransferase	0.00	0.50		
Alanine aminotransferase	0.00	0.64		
Platelets	0.00	0.87		
**T-test**				
**Variables**	***p***	**DF**	**T**	**Cohen's d**
Age	0.37	76.00	-0.33	-0.08
Albumin	0.11	40.28	1.26	0.34
**Mann-Whitney-U test**				
**Variables**	***p***	**U**	**Z**	**r**

Sodium	0.46	639.00	-0.74	0.08
Creatinine	0.00	147.50	-5.82	0.66
Bilirubin	0.66	668.00	-0.44	0.05
International normalized ratio	0.06	532.50	-1.85	0.21
Leukocytes	0.39	627.00	-0.86	0.10
C-reactive protein	0.96	706.00	-0.05	0.01
Aspartate aminotransferase	0.68	439.50	-0.41	0.05
Alanine aminotransferase	0.73	663.50	-0.34	0.04
Platelets	0.04	514.50	-2.03	0.23

Table S7. Validation cohort from Bonn. Analyses separated by statistical method, linear regression models using the backwards method. Abbreviations: MELD = Model for End-Stage Liver Disease score, CLIF-C = Chronic Liver Failure Consortium, AD = Acute decompensation, OF = Organ failure, [1]calculation of Odds ratio not possible due to small patient groups; [2]calculation of significance not possible as one variable is a constant; [3]calculation of Odds ratio due to table >2x2 not possible

CMV DNAemia

**Variables included**	**90-day mortality**, age, sex, aetiology of cirrhosis, ACLF precipitant, Sodium, Creatinine, Bilirubin, Albumin, C-reactive protein, Alkaline phosphatase, Haemoglobin, Time from ACLF to Death				
	**Results:**	Regr. Coeff.	SE	T	*p*
	Sex	0.67	0.33	2.00	0.09
	90-day mortality	-0.69	0.31	-2.26	0.06
	Aetiology of cirrhosis	0.24	0.07	3.58	0.01
	Age	0.03	0.01	3.25	0.02
	Sodium	-0.06	0.02	-3.22	0.02
	Bilirubin	-0.48	0.15	-3.17	0.02
	Albumin	-0.05	0.02	-2.19	0.07
	C-reactive protein	-0.02	0.01	-3.19	0.02
	Corrected R² = 0.587	F=3.485	p=0.073		
**Variables included**	**28-day mortality**, age, sex, aetiology of cirrhosis, ACLF precipitant, Sodium, Creatinine, Bilirubin, Albumin, C-reactive protein, Alkaline phosphatase, Haemoglobin, Time from ACLF to Death				
	**Results:**	Regr. Coeff.	SE	T	*p*
	28-day mortality	0.44	0.16	2.84	0.02
	ACLF precipitant	0.23	0.05	4.84	0.00
	Age	0.02	0.01	2.71	0.03
	Sodium	-0.04	0.01	-3.44	0.01
	Alkaline phosphatase	0.00	0.00	1.94	0.09
	Time from ACLF to Death	0.00	0.00	3.09	0.01
	Corrected R² = 0.651	F=5.348	p=0.017		

EBV DNAemia

**Variables included**	**90-day mortality**, sex, aetiology of cirrhosis, ACLF precipitant, Sodium, Creatinine, Bilirubin, Albumin, Leukocytes, C-reactive protein, Platelets, International normalized ratio, Gamma-glutamyltransferase, Time to death				
	**Results:**	Regr. Coeff.	SE	T	*p*

	ACLF precipitant	0.06	0.03	2.07	0.06
	International normalized ratio	-0.40	0.19	-2.14	0.05
	Corrected R^2 = 0.284	F=4.375	p=0.032		
**Variables included**	**28-day mortality**, sex, aetiology of cirrhosis, ACLF precipitant, Sodium, Creatinine, Bilirubin, Albumin, Leukocytes, C-reactive protein, Platelets, International normalized ratio, Gamma-glutamyltransferase, Time to death				
	**Results:**	Regr. Coeff.	SE	T	*p*
	Sex	0.36	0.07	5.25	0.00
	28-day mortality	0.35	0.06	6.34	0.00
	ACLF precipitant	0.23	0.02	10.33	0.00
	Sodium	-0.04	0.01	-5.01	0.00
	Creatinine	0.28	0.05	5.66	0.00
	Bilirubin	0.43	0.04	11.06	0.00
	Albumin	0.03	0.00	8.31	0.00
	Leukocytes	-0.10	0.01	-7.06	0.00
	C-reactive protein	0.00	0.00	2.95	0.03
	Platelets	0.00	0.00	3.77	0.01
	International normalized ratio	-1.54	0.13	-11.86	0.00
	Time to Death	0.00	0.00	5.47	0.00
	Corrected R^2 = 0.932	F=20.419	p=0.002		

CMV and/or EBV DNAemia

**Variables included**	**90-day mortality**, sex, aetiology of cirrhosis, ACLF precipitant, Sodium, Albumin, Platelets, Alkaline phosphatase, Bilirubin, International normalized ratio				
	**Results:**	Regr. Coeff.	SE	T	*p*
	Aetiology of cirrhosis	0.07	0.03	1.96	0.06
	Sodium	-0.03	0.01	-3.34	0.00
	International normalized ratio	-0.59	0.28	-2.12	0.05
	Corrected R^2 = 0.343	F=5.168	p=0.008		
**Variables included**	**28-day mortality**, sex, aetiology of cirrhosis, ACLF precipitant, Sodium, Albumin, Platelets, Alkaline phosphatase, Bilirubin, International normalized ratio				
	**Results:**	Regr. Coeff.	SE	T	*p*
	Aetiology of cirrhosis	0.07	0.03	1.96	0.06
	Sodium	-0.03	0.01	-3.34	0.00
	International normalized ratio	-0.59	0.28	-2.12	0.05
	Corrected R^2 = 0.343	F=5.168	p=0.008		

Tests vs. CMV DNAemia

**Pearson-Chi-Square test**<br>**Variable**	***p***	**Odds ratio**	**Lower 95% CI**	**Higher 95% CI**
pre-ACLF	0.37	1.96	0.45	8.57
Sex	0.41	0.50	0.09	2.67
28-day mortality	0.16	2.95	0.61	14.18
90-day mortality	0.31	2.19	0.47	10.29
Liver failure	[1]			

Renal failure	0.72	[2]		
Cerebral failure	[1]			
Coagulation failure	[1]			
Circulation failure	[1]			
Respiratory failure	[1]			
Aetiology of cirrhosis	0.05	[3]		
ACLF precipitant	0.19	[3]		
Immunosuppression	[1]			

Biserial rank-correlation

**Variable**	***p***	**Corr. Coeff.**	**DF**
West Haven	[1]		
MELD	0.38	0.54	73.00
CLIF-C AD	0.64	0.64	73.00
CLIF-C OF	[1]		
Child-Pugh category	0.18	0.14	73.00

Shapiro-Wilk test

**Variables**	***p***	**W**
Age	0.61	0.94
Sodium	0.48	0.93
Creatinine	0.99	0.99
Bilirubin	0.04	0.83
Albumin	0.49	0.93
International normalized ratio	0.02	0.79
Leukocytes	0.06	0.84
C-reactive protein	0.25	0.90
Aspartate aminotransferase	0.46	0.93
Alanine aminotransferase	0.59	0.94
Platelets	0.09	0.86
Time to ACLF	0.10	0.86
Time to death	0.00	0.67
Time ACLF to death	0.00	0.60

T-test

**Variables**	***p***	**T**	**DF**	**Cohen's d**
Age	0.22	-0.77	71.00	-0.29
Sodium	0.09	-1.34	71.00	-0.50
Creatinine	0.30	-0.54	71.00	-0.20
Albumin	0.19	-0.89	71.00	-0.33
Leukocytes	0.38	-0.31	71.00	-0.12
C-reactive protein	0.28	-0.59	71.00	-0.22
Aspartate aminotransferase	0.50	0.00	70.00	0.00
Alanine transaminase	0.45	0.13	69.00	0.05
Platelets	0.47	0.09	71.00	0.03
Time to ACLF	0.34	-0.41	24.00	-0.22

Mann-Whitney-U test

**Variables**	***p***	**U**	**Z**	**r**
Bilirubin	0.16	180.00	-1.41	0.17
International normalized ratio	0.81	246.50	-0.24	0.03
Total protein	0.38	83.50	-0.88	0.13
PTT	0.73	240.50	-0.35	0.04
Time to Death	0.60	46.50	-0.53	0.10
Time ACLF to Death	0.84	15.50	-0.20	0.05

Tests vs. EBV DNAemia

Pearson-Chi-Square test

**Variable**	***p***	**Odds ratio**	**Lower 95% CI**	**Higher 95% CI**
pre-ACLF	0.51	1.96	0.26	14.83
Sex	0.11	[2]		
28-day mortality	0.68	1.64	0.16	17.23
90-day mortality	0.87	1.21	0.12	12.60
Liver failure	[1]			

Renal failure	0.80 [2]			
Cerebral failure	[1]			
Coagulation failure	[1]			
Circulation failure	[1]			
Respiratory failure	[1]			
Aetiology of cirrhosis	0.12 [3]			
ACLF precipitant	0.36 [3]			
Immunosuppression	[1]			
**Biserial rank-correlation**				
**Variable**	***p***	**Corr. Coeff.**	**DF**	
West Haven	[1]			
MELD	0.409	0.03	69	
CLIF-C AD	0.530	0.76	69	
CLIF-C OF	[1]			
Child-Pugh category	0.135	0.28	69	
**Shapiro-Wilk test**				
**Variables**	***p***	**W**		
Age	0.47	0.93		
Sodium	0.67	0.95		
Creatinine	0.95	0.98		
Bilirubin	0.08	0.85		
Albumin	0.59	0.94		
International normalized ratio	0.05	0.82		
Leukocytes	0.11	0.86		
C-reactive protein	0.11	0.86		
Aspartate aminotransferase	0.34	0.91		
Alanine aminotransferase	0.77	0.96		
Platelets	0.16	0.87		
Time to ACLF	0.03	0.81		
Time to death	0.00	0.68		
Time ACLF to death	0.00	0.63		
**T-test**				
**Variables**	***p***	**T**	**DF**	**Cohen's d**
Age	0.41	-0.24	67.00	-0.12
Sodium	0.18	-0.94	67.00	-0.49
Creatinine	0.26	0.64	21.87	0.10
Bilirubin	0.00	-6.65	22.45	-1.03
Albumin	0.26	-0.65	67.00	-0.34
Leukocytes	0.21	0.80	67.00	0.41
C-reactive protein	0.24	-0.70	67.00	-0.36
Aspartate aminotransferase	0.35	0.38	64.00	0.27
Alanine transaminase	0.28	0.60	66.00	0.31
Platelets	0.02	2.07	67.00	1.07
**Mann-Whitney-U test**				
**Variables**	***p***	**U**	**Z**	**r**
International normalized ratio	0.01	23.50	-2.80	0.34
PTT	0.20	80.00	-1.29	0.15
Haemoglobin	0.69	75.00	-0.40	0.05
Time to ACLF	0.50	15.50	-0.68	0.14
Time to Death	0.10	0.00	-1.66	0.35
Time ACLF to Death	0.44	4.50	-0.78	0.18

Tests vs. CMV and/or EBV DNAemia

**Pearson-Chi-Square test**				
**Variable**	***p***	**Odds ratio**	**Lower 95% CI**	**Higher 95% CI**
pre-ACLF	0.70	1.30	0.33	5.11
Sex	0.22	0.38	0.07	1.91
28-day mortality	0.32	2.10	0.47	9.43
90-day mortality	0.55	1.561	0.357	6.832
Liver failure	[1]			

Renal failure	0.69 [2]			
Cerebral failure	[1]			
Coagulation failure	[1]			
Circulation failure	[1]			
Respiratory failure	[1]			
Aetiology of cirrhosis	0.17 [3]			
ACLF precipitant	0.19 [3]			
Immunosuppression	[1]			
**Biserial rank-correlation**				
**Variable**	***p***	**Corr. Coeff.**	**DF**	
West Haven	[1]			
MELD	0.40	0.29	75.00	
CLIF-C AD	0.62	0.74	75.00	
CLIF-C OF	[1]			
Child-Pugh category	0.15	0.20	75.00	
**Shapiro-Wilk test**				
**Variables**	***p***	**W**		
Age	0.61	**0.94**		
Sodium	0.48	0.93		
Creatinine	0.99	0.99		
Bilirubin	0.04	0.83		
Albumin	0.49	0.93		
International normalized ratio	0.02	0.79		
Leukocytes	0.06	0.84		
C-reactive protein	0.25	0.90		
Aspartate aminotransferase	0.46	0.93		
Alanine aminotransferase	0.59	0.94		
Platelets	0.09	0.86		
Time to ACLF	0.10	0.86		
Time to death	0.00	0.67		
Time ACLF to death	0.00	0.60		
**T-test**				
**Variables**	***p***	**T**	**DF**	**Cohen's d**
Age	0.33	-0.45	73.00	-0.15
Sodium	0.04	-1.79	73.00	-0.61
Creatinine	0.37	-0.33	73.00	-0.11
Albumin	0.14	-1.08	73.00	-0.37
Leukocytes	0.34	0.42	73.00	0.14
C-reactive protein	0.33	-0.44	73.00	-0.15
Aspartate aminotransferase	0.50	0.00	70.00	0.00
Alanine transaminase	0.36	0.37	71.00	0.13
Platelets	0.10	1.30	73.00	0.44
Time to ACLF	0.34	-0.41	24.00	-0.22
**Mann-Whitney-U test**				
**Variables**	***p***	**U**	**Z**	**r**
Bilirubin	0.03	184.50	-2.19	0.25
International normalized ratio	0.29	259.00	-1.05	0.12
Total protein	0.41	104.50	-0.82	0.12
PTT	0.71	301.50	-0.37	0.04
Time to Death	0.60	46.50	-0.53	0.10
Time ACLF to Death	0.84	15.50	-0.20	0.05

Supplementary sensitivity analyses

Table S8. ACLF-I sensitivity analyses for immunosuppression. Analyses separated by statistical method. Abbreviations: COPD = Chronic obstructive pulmonary disease, TIPS = Transjugular intrahepatic portosystemic stent, MELD = Model for End-Stage Liver Disease score, CLIF-C = Chronic Liver Failure Consortium, AD = Acute decompensation, OF = Organ failure, [1]calculation of Odds ratio not possible due to small patient groups; [2]calculation of Odds ratio due to table >2x2 not possible

**Tests vs. CMV DNAemia**					
**Pearson-Chi-Square test**					
**Variable**	***p***		**Odds ratio**	**Lower 95% CI**	**Higher 95% CI**
ACLF	0.00		4.13	1.64	10.41
Pre-ACLF	0.91		1.09	0.23	5.21
Sex	0.06		0.44	0.18	1.06
28-day mortality	0.50		1.57	0.41	5.98
90-day mortality	0.79		1.15	0.40	3.34
Liver failure	0.00		4.08	1.58	10.55
Renal failure	0.74		1.25	0.34	4.66
Cerebral failure	0.07		3.57	0.83	15.37
Coagulation failure	0.35		1.88	0.49	7.31
Circulation failure	0.83		1.16	0.31	4.30
Respiratory failure	0.66		1.65	0.18	15.44
Diabetes	0.18		0.50	0.18	1.41
COPD	0.33	[1]			
Heart failure	0.37	[1]			
Hypertension	0.03		0.21	0.05	0.93
Coronary artery disease	0.14	[1]			
Chronic kidney disease	0.43	[1]			
Ascites	0.55		1.32	0.53	3.27
Hepatic encephalopathy	0.15		1.93	0.78	4.77
Gastrointestinal bleeding	0.44		0.64	0.21	2.00
Bacterial infections	0.56		1.29	0.55	3.06
Viral infections	0.64		1.71	0.18	15.96
Vasopressors	0.14		3.46	0.60	19.99
Dialysis	0.49	[1]			
TIPS	0.24		0.42	0.09	1.87
Beta blockers	0.01		0.25	0.08	0.79
Antibiotic prophylaxis	0.43		1.51	0.54	4.23
Oxygen supplementation	0.20		2.19	0.65	7.40
Mechanical ventilation	0.29		2.07	0.53	8.17
Any oxygen supplement	0.49		2.22	0.22	22.23
Aetiology of cirrhosis	0.16	[2]			
**Biserial rank-correlation**					
**Variable**	***p***		**Corr. Coeff.**	**DF**	
ACLF grade	0.65		0.08	38	
West Haven	0.00		0.24	180	
MELD	0.46		0.25	46	
MELD sodium	0.07		0.46	173	
CLIF-C ACLF	0.11		0.52	173	
CLIF-C AD	0.08		0.51	179	
CLIF-C OF	0.02		0.64	179	
Child-Pugh score	0.01		0.32	180	
Child-Pugh category	0.10		0.18	180	
**Shapiro-Wilk test**					
**Variables**	***p***		**W**		
Age	0.05		0.97		
Sodium	0.05		0.97		

Creatinine	0.00	0.77		
Bilirubin	0.00	0.72		
Albumin	0.55	0.99		
International normalized ratio	0.00	0.78		
Leukocytes	0.00	0.87		
C-reactive protein	0.00	0.74		
Aspartate aminotransferase	0.00	0.60		
Alanine aminotransferase	0.00	0.66		
Alkaline phosphatase	0.00	0.65		
Gamma-glutamyltransferase	0.00	0.27		
Platelets	0.00	0.85		
**T-test**				
**Variables**	***p***	**DF**	**T**	**Cohen's d**
Sodium	0.38	178	-0.30	-0.07
Albumin	0.36	176	-0.36	-0.08
**Mann-Whitney-U test**				
**Variables**	***p***	**U**	**Z**	**r**
Age	0.14	1525.5	-1.46	0.11
Creatinine	0.74	1792.5	-0.33	0.02
Bilirubin	0.02	1311.5	-2.36	0.18
International normalized ratio	0.96	1860.0	-0.05	0.00
Leukocytes	0.01	1260.0	-2.54	0.19
C-reactive protein	0.23	1358.5	-1.21	0.09
Aspartate aminotransferase	0.01	739.5	-2.58	0.22
Alanine aminotransferase	0.15	1420.5	-1.45	0.11
Alkaline phosphatase	0.01	737.0	-2.51	0.21
Gamma-glutamyltransferase	0.02	1285.5	-2.32	0.17
Platelets	0.63	1444.5	-0.48	0.04
**Tests vs. EBV DNAemia**				
**Pearson-Chi-Square test**				
**Variable**	***p***	**Odds ratio**	**Lower 95% CI**	**Higher 95% CI**
ACLF	0.01	4.34	1.38	13.66
Pre-ACLF	0.28	[1]		
Sex	0.12	0.42	0.14	1.27
28-day mortality	0.88	0.85	0.10	6.99
90-day mortality	0.69	0.73	0.16	3.44
Liver failure	0.37	1.86	0.48	7.23
Renal failure	0.64	1.46	0.30	7.11
Cerebral failure	0.46	[1]		
Coagulation failure	0.99	1.01	0.12	8.48
Circulation failure	0.71	1.35	0.28	6.57
Respiratory failure	0.33	2.92	0.30	28.11
Diabetes	0.12	0.32	0.07	1.46
COPD	0.46	[1]		
Heart failure	0.44	2.32	0.25	21.40
Hypertension	0.21	0.39	0.08	1.80
Coronary artery disease	0.88	0.85	0.10	6.99
Chronic kidney disease	0.54	[1]		
Ascites	0.18	2.42	0.65	9.02
Hepatic encephalopathy	0.68	1.29	0.38	4.34
Gastrointestinal bleeding	0.42	0.54	0.12	2.51
Bacterial infections	0.33	1.73	0.57	5.21
Viral infections	0.56	[1]		
Vasopressors	0.02	6.33	1.05	38.17
Dialysis	0.22	3.92	0.38	40.44
TIPS	0.30	0.35	0.04	2.80
Beta blockers	0.19	0.45	0.13	1.53
Antibiotic prophylaxis	0.22	0.29	0.04	2.34
Oxygen supplementation	0.43	1.89	0.38	9.45
Mechanical ventilation	0.90	1.15	0.14	9.76

Any oxygen supplement	0.22	3.92	0.38	40.44
Aetiology of cirrhosis	0.92	2		
**Biserial rank-correlation**				
**Variable**	***p***	**Corr. Coeff.**	**DF**	
ACLF grade	0.45	0.31	34	
West Haven	0.05	0.25	170	
MELD	0.29	0.17	41	
MELD sodium	0.45	0.36	167	
CLIF-C ACLF	0.22	0.37	167	
CLIF-C AD	0.15	0.39	169	
CLIF-C OF	0.32	0.69	169	
Child-Pugh score	0.84	0.29	170	
Child-Pugh category	0.84	0.23	170	
**Shapiro-Wilk test**				
**Variables**	***p***	**W**		
Age	0.42	0.98		
Sodium	0.04	0.97		
Creatinine	0.00	0.78		
Bilirubin	0.00	0.72		
Albumin	0.68	0.99		
International normalized ratio	0.00	0.76		
Leukocytes	0.00	0.86		
C-reactive protein	0.00	0.75		
Aspartate aminotransferase	0.00	0.60		
Alanine aminotransferase	0.00	0.64		
Alkaline phosphatase	0.00	0.65		
Gamma-glutamyltransferase	0.00	0.29		
Platelets	0.00	0.91		
**T-test**				
**Variables**	***p***	**DF**	**T**	**Cohen's d**
Age	0.38	168	-0.32	-0.09
Albumin	0.23	20.22	-0.72	-0.14
**Mann-Whitney-U test**				
**Variables**	***p***	**U**	**Z**	**r**
Sodium	0.09	795.0	-1.69	0.13
Creatinine	0.88	1066.0	-0.15	0.01
Bilirubin	0.38	936.5	-0.88	0.07
International normalized ratio	0.79	1044.0	-0.27	0.02
Leukocytes	0.02	684.0	-2.29	0.18
C-reactive protein	0.03	538.5	-2.24	0.18
Aspartate aminotransferase	0.54	511.5	-0.62	0.06
Alanine aminotransferase	0.09	647.0	-1.67	0.13
Alkaline phosphatase	0.13	512.0	-1.53	0.13
Gamma-glutamyltransferase	0.48	872.5	-0.70	0.05
Platelets	0.84	710.5	-0.20	0.02
**Tests vs. CMV and/or EBV DNAemia**				
**Pearson-Chi-Square test**				
**Variable**	***p***	**Odds ratio**	**Lower 95% CI**	**Higher 95% CI**
ACLF	0.00	3.68	1.65	8.22
Pre-ACLF	0.66	0.71	0.15	3.30
Sex	0.03	0.44	0.20	0.93
28-day mortality	0.60	1.38	0.42	4.49
90-day mortality	0.79	0.88	0.33	2.30
Liver failure	0.01	2.99	1.28	7.00
Renal failure	0.88	1.09	0.34	3.49
Cerebral failure	0.25	2.27	0.54	9.56
Coagulation failure	0.41	1.65	0.49	5.51
Circulation failure	0.98	1.02	0.32	3.22
Respiratory failure	0.35	2.24	0.39	12.71
Diabetes	0.08	0.46	0.19	1.11

COPD	0.23 [1]			
Heart failure	0.89	0.86	0.10	7.62
Hypertension	0.02	0.29	0.10	0.87
Coronary artery disease	0.25	0.31	0.04	2.48
Chronic kidney disease	0.33 [1]			
Ascites	0.31	1.50	0.69	3.26
Hepatic encephalopathy	0.39	1.42	0.64	3.15
Gastrointestinal bleeding	0.20	0.52	0.19	1.43
Bacterial infections	0.49	1.29	0.63	2.67
Viral infections	0.91	1.14	0.12	10.50
Vasopressors	0.10	3.46	0.74	16.17
Dialysis	0.75	1.46	0.15	14.43
TIPS	0.16	0.42	0.12	1.45
Beta blockers	0.02	0.35	0.15	0.84
Antibiotic prophylaxis	0.96	1.03	0.41	2.59
Oxygen supplementation	0.30	1.79	0.59	5.42
Mechanical ventilation	0.67	1.34	0.35	5.14
Any oxygen supplement	0.22	3.00	0.48	18.65
Aetiology of cirrhosis	0.32 [2]			
**Biserial rank-correlation**				
**Variable**	***p***	**Corr. Coeff.**	**DF**	
ACLF grade	0.75	0.11	43	
West Haven	0.00	0.25	192	
MELD	0.64	0.20	48	
MELD sodium	0.10	0.42	185	
CLIF-C ACLF	0.09	0.46	185	
CLIF-C AD	0.04	0.44	191	
CLIF-C OF	0.03	0.62	191	
Child-Pugh score	0.06	0.29	192	
Child-Pugh category	0.21	0.20	192	
**Shapiro-Wilk test**				
**Variables**	***p***	**W**		
Age	0.24	0.98		
Sodium	0.05	0.97		
Creatinine	0.00	0.78		
Bilirubin	0.00	0.73		
Albumin	0.58	0.99		
International normalized ratio	0.00	0.78		
Leukocytes	0.00	0.88		
C-reactive protein	0.00	0.76		
Aspartate aminotransferase	0.00	0.60		
Alanine aminotransferase	0.00	0.65		
Alkaline phosphatase	0.00	0.65		
Gamma-glutamyltransferase	0.00	0.28		
Platelets	0.00	0.85		
**T-test**				
**Variables**	***p***	**DF**	**T**	**Cohen's d**
Age	0.15	45.95	-1.21	-0.22
Albumin	0.35	188	-0.38	-0.07
**Mann-Whitney-U test**				
**Variables**	***p***	**U**	**Z**	**r**
Sodium	0.36	2533.5	-0.92	0.07
Creatinine	0.43	2569.5	-0.79	0.06
Bilirubin	0.05	2220.0	-1.96	0.14
International normalized ratio	0.91	2774.0	-0.11	0.01
Leukocytes	0.00	1941.0	-2.84	0.21
C-reactive protein	0.08	1883.0	-1.77	0.13
Aspartate aminotransferase	0.03	1193.0	-2.18	0.18
Alanine aminotransferase	0.80	2436.5	-0.26	0.02
Alkaline phosphatase	0.01	1207.0	-2.50	0.20
Gamma-glutamyltransferase	0.03	2041.0	-2.14	0.16

Platelets	0.49	1973.5	-0.70	0.05

Table S9. Validation cohort sensitivity analyses for immunosuppression. Analyses separated by statistical method. Abbreviations: MELD = Model for End-Stage Liver Disease score, CLIF-C = Chronic Liver Failure Consortium, AD = Acute decompensation, OF = Organ failure, [1]calculation of Odds ratio not possible due to small patient groups; [2]calculation of significance not possible as one variable is a constant; [3]calculation of Odds ratio due to table >2x2 not possible

**Tests vs. CMV DNAemia**				
**Pearson-Chi-Square test**				
**Variable**	***p***	**Odds ratio**	**Lower 95% CI**	**Higher 95% CI**
ACLF	0.00	40.63	12.04	137.10
pre-ACLF	0.06	3.61	0.90	14.45
Sex	0.14	0.52	0.22	1.25
28-day mortality	0.72	0.83	0.28	2.40
90-day mortality	0.84	0.92	0.39	2.17
Liver failure	0.01	10.50	1.06	104.42
Renal failure	0.00	24.32	9.26	63.87
Cerebral failure	0.16	[1]		
Coagulation failure	[2]			
Circulation failure	[2]			
Respiratory failure	[2]			
Precipitant	0.35			
ACLF precipitant	0.04	[3]		
**Biserial rank-correlation**				
**Variable**	***p***	**Corr. Coeff.**	**DF**	
ACLF grade	0.62	0.25	38	
West Haven	0.00	0.63	148	
MELD	0.86	0.15	148	
CLIF-C ACLF	0.00	0.68	148	
CLIF-C AD	0.11	0.74	38	
CLIF-C OF	0.46	0.56	110	
Child-Pugh category	0.00	0.64	111	
**Shapiro-Wilk test**				
**Variables**	***p***	**W**		
Age	0.45	0.99		
Sodium	0.00	0.94		
Creatinine	0.00	0.74		
Bilirubin	0.00	0.39		
Albumin	0.25	0.99		
International normalized ratio	0.00	0.86		
Leukocytes	0.00	0.89		
C-reactive protein	0.00	0.74		
Aspartate aminotransferase	0.00	0.45		
Alanine aminotransferase	0.00	0.59		
Platelets	0.00	0.89		
**T-test**				
**Variables**	***p***	**DF**	**T**	**Cohen's d**
Age	0.42	146	-0.21	-0.04
Albumin	0.29	143	-0.57	-0.11
**Mann-Whitney-U test**				
**Variables**	***p***	**U**	**Z**	**r**
Sodium	0.19	1684.5	-1.33	0.11
Creatinine	0.00	647.5	-6.00	0.49
Bilirubin	0.33	1763.5	-0.97	0.08
International normalized ratio	0.02	1479.5	-2.27	0.19
Leukocytes	0.75	1907.5	-0.32	0.03

C-reactive protein	0.08	1594	-1.73	0.14
Aspartate aminotransferase	0.06	1236	-1.86	0.16
Alanine aminotransferase	0.48	1735.5	-0.71	0.06
Platelets	0.22	1703.5	-1.24	0.10

Tests vs. EBV DNAemia

Pearson-Chi-Square test

**Variable**	***p***	**Odds ratio**	**Lower 95% CI**	**Higher 95% CI**
ACLF	0.00	46.88	9.50	231.32
pre-ACLF	0.09	4.33	0.68	27.35
Sex	0.02	0.20	0.04	0.88
28-day mortality	0.84	0.87	0.23	3.28
90-day mortality	0.81	1.13	0.40	3.21
Liver failure	0.67	[1]		
Renal failure	0.00	25.25	7.79	81.83
Cerebral failure	0.29	[1]		
Coagulation failure	[2]			
Circulation failure	[2]			
Respiratory failure	[2]			
Aetiology of cirrhosis	0.29	[3]		
ACLF precipitant	0.20	[3]		

Biserial rank-correlation

**Variable**	***p***	**Corr. Coeff.**	**DF**
ACLF grade	0.11	0.32	27
West Haven	0.00	0.61	133
MELD	0.77	0.18	133
CLIF-C ACLF	0.00	0.70	133
CLIF-C AD	0.01	0.92	27
CLIF-C OF	0.63	0.47	106
Child-Pugh category	0.00	0.63	96

Shapiro-Wilk test

**Variables**	***p***	**W**
Age	0.71	0.99
Sodium	0.00	0.93
Creatinine	0.00	0.74
Bilirubin	0.00	0.50
Albumin	0.42	0.99
International normalized ratio	0.00	0.87
Leukocytes	0.00	0.89
C-reactive protein	0.00	0.72
Aspartate aminotransferase	0.00	0.44
Alanine aminotransferase	0.00	0.57
Platelets	0.00	0.89

T-test

**Variables**	***p***	**DF**	**T**	**Cohen's d**
Age	0.16	131	-1.02	-0.25
Albumin	0.49	128	0.02	0.01

Mann-Whitney-U test

**Variables**	***p***	**U**	**Z**	**r**
Sodium	0.40	996	-0.85	0.07
Creatinine	0.00	316.5	-5.12	0.44
Bilirubin	0.81	1091.5	-0.24	0.02
International normalized ratio	0.55	1037	-0.59	0.05
Leukocytes	0.89	1107.5	-0.14	0.01
C-reactive protein	0.13	887	-1.53	0.13
Aspartate aminotransferase	0.03	555	-2.24	0.21
Alanine aminotransferase	0.28	942	-1.08	0.09
Platelets	0.20	927	-1.28	0.11

Tests vs. CMV and/or EBV DNAemia

Pearson-Chi-Square test

Variable	*p*	Odds ratio	Lower 95% CI	Higher 95% CI
ACLF	0.00	28.13	9.61	82.34
pre-ACLF	0.08	2.89	0.86	9.74
Sex	0.07	0.45	0.19	1.08
28-day mortality	0.84	0.90	0.33	2.45
90-day mortality	0.83	0.91	0.40	2.09
Liver failure	0.02	9.33	0.94	92.55
Renal failure	0.00	18.94	7.66	46.85
Cerebral failure	0.14	[1]		
Coagulation failure	[2]			
Circulation failure	[2]			
Respiratory failure	[2]			
Aetiology of cirrhosis	0.34	[3]		
ACLF precipitant	0.05	[3]		
**Biserial rank-correlation**				
**Variable**	***p***	**Corr. Coeff.**	**DF**	
ACLF grade	0.59	0.25	39	
West Haven	0.00	0.60	152	
MELD	0.88	0.17	152	
CLIF-C ACLF	0.00	0.65	152	
CLIF-C AD	0.08	0.74	39	
CLIF-C OF	0.78	0.52	113	
Child-Pugh category	0.00	0.62	114	
**Shapiro-Wilk test**				
**Variables**	***p***	**W**		
Age	0.51	0.99		
Sodium	0.00	0.94		
Creatinine	0.00	0.74		
Bilirubin	0.00	0.39		
Albumin	0.28	0.99		
International normalized ratio	0.00	0.86		
Leukocytes	0.00	0.89		
C-reactive protein	0.00	0.74		
Aspartate aminotransferase	0.00	0.46		
Alanine aminotransferase	0.00	0.60		
Platelets	0.00	0.89		
**T-test**				
**Variables**	***p***	**DF**	**T**	**Cohen's d**
Age	0.42	150	-0.194	-0.036
Albumin	0.25	147	-0.666	-0.125
**Mann-Whitney-U test**				
**Variables**	***p***	**U**	**Z**	**r**
Sodium	0.19	1891.5	-1.32	0.11
Creatinine	0.00	824	-5.82	0.47
Bilirubin	0.43	2017	-0.79	0.06
International normalized ratio	0.05	1741	-1.97	0.16
Leukocytes	0.93	2182	-0.09	0.01
C-reactive protein	0.06	1764	-1.85	0.15
Aspartate aminotransferase	0.03	1270.5	-2.13	0.18
Alanine aminotransferase	0.28	1863	-1.07	0.09
Platelets	0.27	1942.5	-1.10	0.09

Table S10. Missing data for ACLF-I and Validation cohort. Number of missing data points and percentage of the respective cohort.

**ACLF-I**	
ACLF	0
Pre-ACLF	0
Sex	0

28-day mortality	0
90-day mortality	0
Liver failure	0
Renal failure	0
Cerebral failure	0
Coagulation failure	0
Circulation failure	1 (0.5)
Respiratory failure	0
Diabetes	0
COPD	0
Heart failure	0
Hypertension	0
Coronary artery disease	0
Chronic kidney disease	0
Ascites	0
Hepatic encephalopathy	0
Gastrointestinal bleeding	0
Bacterial infections	0
Viral infections	4 (1.9)
Vasopressors	0
Dialysis	0
TIPS	0
Immunosuppression	13 (6.2)
Beta blockers	12 (5.7)
Antibiotic prophylaxis	12 (5.7)
Oxygen supplementation	10 (4.7)
Mechanical ventilation	0
Any oxygen supplement	0
Aetiology of cirrhosis	0
ACLF grade	0
West Haven	0
MELD	8 (3.8)
MELD sodium	8 (3.8)
CLIF-C ACLF	1 (0.5)
CLIF-C AD	1 (0.5)
CLIF-C OF	0
Child-Pugh score	0
Child-Pugh category	0
Age	0
Albumin	2 (0.9)
Sodium	0
Creatinine	0
Bilirubin	0
International normalized ratio	0
Leukocytes	1 (0.5)
C-reactive protein	15 (7.1)
Aspartate aminotransferase	54 (25.6)
Alanine aminotransferase	8 (3.8)
Alkaline phosphatase	38 (18.0)
Gamma-glutamyltransferase	7 (3.3)
Platelets	0

**Validation cohort**	
ACLF	0
pre-ACLF	0
Sex	0
28-day mortality	0
90-day mortality	0
Liver failure	0
Renal failure	0
Cerebral failure	0
Coagulation failure	0
Circulation failure	0
Respiratory failure	0
Precipitant	0
ACLF precipitant	6 (8.5)
ACLF grade	0
West Haven	0
MELD	0
CLIF-C ACLF	0
CLIF-C AD	0
CLIF-C OF	0
Child-Pugh category	0
Age	0
Sodium	0
Creatinine	0
Bilirubin	0
Albumin	3 (2.0)
International normalized ratio	0
Leukocytes	0
C-reactive protein	0
Aspartate aminotransferase	19 (12.4)
Alanine aminotransferase	3 (2.0)
Platelets	0

Supplementary methods

Information for in-house CMV and EBV qPCR experiments

Sample collection, storage and general handling

211 patients admitted to the Department of Internal Medicine I, University Hospital Frankfurt, Germany, from January 2021 to June 2023 were enrolled in the ACLF-I cohort. Serum samples of respective patients were collected at the time of inclusion. Likewise, 78 patients admitted to the Department of Internal Medicine III, University Hospital Aachen and Department of Internal Medicine IV, Jena University Hospital, from September 2010 to July 2019 and 75 patients admitted to the Medical Clinic and Polyclinic I, University Hospital Bonn, from June 2019 to April 2023 were collected as part of one external validation cohort. Blood sera were collected in all three centers according to standardized clinical practice. All collected samples were aliquoted and stored at -80°C immediately. During transport samples were maintained frozen. All samples underwent a minimal number of freeze-thaw cycles due to previous aliquoting of samples. Moreover, samples were thawed on ice to minimize the impact of freeze-thawing. Since viral DNA is stable for a largely extended time-period without major loss at applied storage conditions, as previously shown [1], we determined viral DNA and not the amount of infectious particles. DNA extraction efficiency, amplification efficiency, DNA contaminations and inter-assay variations were monitored and validated as described in the following sections.

Nucleic Acid Extraction

Total viral nucleic acids within patient sera were extracted using the High Pure Viral Nucleic Acid Kit (Roche Diagnostics). The manufacturer´s instructions was modified as follows. 200 µl of respective patient serum was mixed with 200 µl Binding buffer supplemented with 4 µl poly A, 50 µl Proteinase K and 10 µl of Phocine herpesvirus

(PhHV) as extraction control. PhHV provided with the Lightmix Modular PhHV spiked Extraction Control (660) kit (Roche Diagnostics) was prepared according to manufacturer's instructions. After addition to sera, the reaction mixture was vortexed briefly and incubated for 30 min at 72°C. Subsequent extraction was performed according to instructions of the kit´s manufacturer. For elution, elution buffer was preheated to 70°C and viral nucleic acids were eluted in 50 µl of elution buffer. Extracted viral nucleic acids were stored immediately at -20°C and processed within two weeks. Due to limited sample size extracted viral nucleic acids were not quantified but totally processed in subsequent qPCR experiments. DNA or RNA contamination of extracted samples were assed by qPCR (see qPCR validation and data analysis).

qPCR oligonucleotides and target information

For all qPCR experiments different hydrolysis probes targeting either viral DNA of human Cytomegalovirus (CMV) or Epstein-Barr virus (EBV) and the PhHV extraction control were used in a multiplex assay. All hydrolysis probe kits were obtained by Roche diagnostics. Respective hydrolysis probes, the targeting regions and the length of the amplicon are depicted in table S11. Primer sequences and exact targeting regions are not available.

Table S11: Hydrolysis probes and corresponding amplicon details.

**Kit name**	**Cat no.**	**Amplicon location**	**Amplicon length**
Lightmix® Modular PhHV spiked Extraction Control (660)	07093802001	N/A	85 bp
Lightmix® Modular Epstein-Barr virus (610)	10097710001	BNRF1 gene	78 bp

Lightmix® Modular human Cytomegalovirus (500)	08997837001	US17 gene<br>F fragment gene	151 bp<br>61 bp

qPCR protocol

All qPCR experiments were performed manually as multiplex-assay. The qPCR reaction for mixture was prepared as follows: 4 µl of Roche Lightcycler Multiplex RNA Virus/ DNA master (Roche Diagnostics) was mixed with 0.5 µl of respective hydrolysis probes (Table S2) and 2.5 µl of PCR-grade water (Roche Diagnostics) to a final volume of 8 µl per reaction. Then, 8 µl of the master mix was added per well of a white lightcycler 480 Multiwell plate (Roche Diagnostics). Finally, 12 µl of previously eluted viral nucleic acid extracts were added per well. All samples were measured in duplicate. In each qPCR run a positive control for CMV and EBV provided with the hydrolysis probe kits was run alongside to asses inter-assay variations. The multiplex qPCR was carried out in a Lightcycler 480 instrument II (Roche Diagnostics). The qPCR program consisted of an initial denaturation step at 95°C for 5 min and subsequent 45 cycles with denaturation at 95°C for 5 s, annealing at 60°C for 15 s and elongation at 72°C for 15 s with a ramp rate of 4.4, 2.2 and 4.4 respectively. After the final cycle the system was cooled down to 4°C. The CMV specific hydrolysis probe was detected using a 440-488 nm filter, a quant factor of 10 and a maximal integration time of 1 sec. The EBV specific hydrolysis probes was detected using a 533-610 nm filter with a quant factor of 10 and a maximum integration time of 2 sec. The PhHV specific hydrolysis probes were detected using a 618-660 nm filter, a quant factor of 10 and a maximum integration time of 3 sec.

qPCR validation and data analysis

Since the qPCR experiments were performed as multiplex-assays, preliminary a color compensation file was generated using the positive controls provided with the respective hydrolysis probe kits. The color compensation was applied to all subsequent qPCR experiments prior to analysis. Ct values were determined by applying the Second Derivative maximum algorithm using the Lightcycler® 480 software version 1.5.1 (Roche Diagnostics). To determine the lower limit of detection (LLOD) a dilution series ranging from 2 to 10^6 of CMV and EBV target molecules provided with the hydrolysis probe kits was performed. The CV values for each dilution series was assessed. 7 total copies per well resulted in successful detection with more than 95% confidence and a CV of 0.92% and 1.26% for the Ct values of CMV and EBV respectively. Thus, 7 copies per well were determined as the LLOD for CMV and EBV detection. The linear range with high coefficient of determination (R^2) for the linear regression models was determined as 10 to 10^6 target molecules per well for CMV and EBV. The coefficient of determination R^2 values of the linear regression models were 0.9998 and 0.9958 for CMV and EBV respectively. Thus, 10 target molecules per well were set as the lower limit of quantification (LLOQ) and 10^6 as the upper limit of quantification (ULQ) for CMV and EBV detection. Further, for CMV the qPCR efficiency was 99.33% and the amplification factor was 1.99. Accordingly, for EBV qPCR efficiency was 99.13% and the amplification factor was 1.99. The linear regression models with respective linear functions including slope and Y-intercept are depicted in Figure S1.

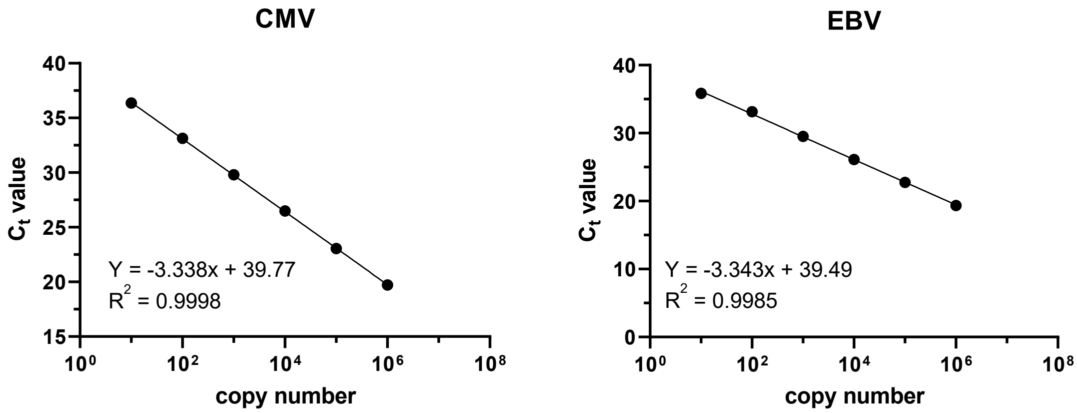

Fig. S1: Linear regression models of CMV and EBV standard curve for absolute quantification.

Ct values were converted into total copy number per well by extrapolation using the linear regression models. Viral loads were calculated by dividing the calculated copy numbers with the qPCR sample volume (12 µl) and by dividing with the concentration factor during viral nucleic acid extraction (for-fold concentration). Accordingly, 7 copies per well corresponding to the LLOD were converted into 150 copies/ml. Thus, 150 copies/ ml were set as the cut-off value for CMV and EBV detection. In this regards, all samples below the cut-off value were considered negative and conclusively non DNAemic. To assess nucleic acid contamination, the elution buffer which was used during viral nucleic acid extraction was measured with each qPCR run. All qPCR experiments in which the elution buffer was below the LLOD in multiple replicates were considered valid. To validate viral nucleic acid extraction quality, the Ct value of the PhHV extraction controls were determined. All samples with a Ct-value ranging between 27 and 30 for the PhHV extraction control were considered acceptable and thus included in subsequent analysis. The coefficient of variation (CV) of the Ct values for the PhHV extraction control for all samples within the ACLF-I cohort was 4.7%. The CV of the Ct values from the Aachen/Jena validation cohort was 4.9% and from the second validation cohort 3.1% for the extraction control. To asses inter-assay

variations for CMV and EBV viral nucleic acid detection, the CV of the Ct-value for the CMV and EBV positive controls run alongside each qPCR run was determined. Within the ACLF-I cohort the CV values were 1.69% for CMV and 0.94% for EBV positive controls. Within the Aachen/ Jena validation cohort the CV values were 1.3% for CMV and 0.44% for EBV positive controls. Within the Bonn validation cohort the CV values were 0.5% for CMV and 1.0% for EBV positive controls.

Experimental design

CMV and EBV DNAemia within all patient samples of the ACLF-I and the validation cohort were determined by the above mentioned qPCR method. Upon applying the quality control criteria the calculated DNAemia of all included patients were correlated to their respective clinical data. Presence of DNAemia (CMV and EBV combined or solely) within each subgroup (table S1) was determined as a proportion of the total number of each subgroup (Fig 1C and Fig 4A). Likewise, to asses DNAemia associated organ failure and inflammation the DNAemia detected proportion within the total collective was calculated (Fig 1.D, Fig 2 & Fig. 3) and correlated to respective parameters describing either organ failure or inflammation. The impact of DNAemia on the ACLF grade was analyzed by calculating the proportion of DNAemic patients within each ACLF grade subgroup. The total number of each subgroup and the proportion of DNAemic patients are indicated in table 1. All statistical methods to analyze significance are described in the materials and method section of the manuscript.

Cytokine analysis – Data analysis and validation

For cytokine profiling of respective serum samples a custom multiplex magnetic bead panel using the Luminex Discovery Assay Human Premixed Multi-Analyte Kit (Luminex Corporation) was performed. The multiplex assay was carried out as described in the materials and methods section. Each sample was measured in duplicates. A three fold

dilution series with six spiked standards of positive controls for respective cytokines and corresponding negative controls were measured alongside with each run to asses intra- and inter-assay variations. Mean CV values for an intermediate positive control within the linear range of each target cytokine are depicted in table S2. Runs with a CV below 15% for each positive control were considered valid. Accordingly, samples with a CV above 15% were excluded to maintain assay accuracy. Limit of detection was defined by a minimum of 50 magnetic beads bound to the target analyte. Displayed final Log_2 Fold changes were determined by calculating the binary logarithm of background subtracted fluorescence intensities normalized to respective negative controls corresponding to each run.

Table S12: Averaged CV and recovery rates of target analytes.

Target analyte	n	average CV (%)	average recovery (%)
CCL2/MCP-1	3	1.00	98.32
CCL3/MIP-1a	3	2.73	100.76
CCL4/MIP-1b	3	0.34	100.15
CXCL10/IP-10	3	2.13	100.19
GM-CSF	3	2.10	100.30
IFN-gamma	3	2.16	99.19
IL-10	3	3.99	101.86
IL-18	3	4.55	102.12
IL-1a	3	1.49	101.40
IL-1b	3	2.34	99.05
IL-1ra	3	2.70	98.92
IL-2	3	0.84	99.50
MIF	3	1.33	100.05
TNF-a	3	1.33	100.56

Supplementary references

1. Bajaj, J.S., et al., *Survival in infection-related acute-on-chronic liver failure is defined by extrahepatic organ failures.* Hepatology, 2014. **60**(1): p. 250-6.
2. Giamarellos-Bourboulis, E.J., et al., *Interferon-gamma driven elevation of CXCL9: a new sepsis endotype independently associated with mortality.* EBioMedicine, 2024. **109**: p. 105414.
3. Berres, M.L., et al., *CXCL9 is a prognostic marker in patients with liver cirrhosis receiving transjugular intrahepatic portosystemic shunt.* Journal of Hepatology, 2015. **62**(2): p. 332-339.
4. Noor, M.T. and P. Manoria, *Immune Dysfunction in Cirrhosis.* J Clin Transl Hepatol, 2017. **5**(1): p. 50-58.
5. Jalan, R., et al., *Development and validation of a prognostic score to predict mortality in patients with acute-on-chronic liver failure.* J Hepatol, 2014. **61**(5): p. 1038-47.
