# Supplementary material for: Higher prevalence of cytomegalovirus and Epstein–Barr virus in acute-on-chronic liver failure
Source: JHEP Rep. 2025 Oct 9;8(4):101627. doi: 10.1016/j.jhepr.2025.101627 (PMC13019566; doi:10.1016/j.jhepr.2025.101627)
Supplement: Multimedia component 2 [file mmc2.docx]

**JHEP Reports**

**CTAT methods**

Tables for a “Complete, Transparent, Accurate and Timely account” (CTAT) are now mandatory for all revised submissions. The aim is to enhance the reproducibility of methods.

- Only include the parts relevant to your study
- Refer to the CTAT in the main text as ‘Supplementary CTAT Table’
- Do not add subheadings
- Add as many rows as needed to include all information
- Only include one item per row

**If the CTAT form is not relevant to your study, please outline the reasons why:**

|  |
| --- |

- 1. **Antibodies**

| **Name** | **Citation** | **Supplier** | **Cat no.** | **Clone no.** |
| --- | --- | --- | --- | --- |
| **-** | **-** | **-** | **-** | **-** |

- 1. **Cell lines**

| **Name** | **Citation** | **Supplier** | **Cat no.** | **Passage no.** | **Authentication test method** |
| --- | --- | --- | --- | --- | --- |
| **-** | **-** | **-** | **-** | **-** | **-** |

- 1. **Organisms**

| **Name** | **Citation** | **Supplier** | **Strain** | **Sex** | **Age** | **Overall n number** |
| --- | --- | --- | --- | --- | --- | --- |
| **-** | **-** | **-** | **-** | **-** | **-** | **-** |

- 1. **Sequence based reagents**

| **Name** | **Sequence** | **Supplier** |
| --- | --- | --- |
| LightMix Modular CMV (500)  Cat no. 08997837001 | N/A | Roche Diagnostics Deutschland GmbH |
| LightMix Modular EBV (610)  Cat no. 10097710001 | N/A | Roche Diagnostics Deutschland GmbH |
| LightMix Modular PhHV spiked Extraction Control (660)  Cat no. 07093802001 | N/A | Roche Diagnostics Deutschland GmbH |

- 1. **Biological samples**

| **Description** | **Source** | **Identifier** |
| --- | --- | --- |
|  |  |  |

- 1. **Deposited data**

| **Name of repository** | **Identifier** | **Link** |
| --- | --- | --- |
|  |  |  |

- 1. **Software**

| **Software name** | **Manufacturer** | **Version** |
| --- | --- | --- |
| Lightcycler® 480 SW | Roche Diagnostics Ltd. | 1.5.1 |
| xPONENT software | Luminex Corp. | Version 4.3 Build 229 |

- 1. **Other (e.g. drugs, proteins, vectors etc.)**

| **Kit name** | **Cat no.** | **Supplier** |
| --- | --- | --- |
| High Pure Viral Nucleic Acid Kit | 11858874001 | Roche Diagnostics, (Mannheim, Germany) |
| LightCycler Multiplex RNA Virus Master | 06754155001 | Roche Diagnostics (Mannheim, Germany) |
| Luminex Discovery Assay Human Premixed Multi-Analyte Kit | LXSAHM-16 | R&D Systems (Minneapolis, MN, USA) |
| MAGPIX™ Drive Fluid PLUS | 4050030 | Luminex Corp. (Austin, TX, USA) |
| MAGPIX™ Calibration Kit | MPXCALK25 | Luminex Corp. (Austin, TX, USA) |
| MAGPIX™ Performance Verification Kit | MPXPVERK25 | Luminex Corp. (Austin, TX, USA) |
| ARCHITECT CMV IgG Reagents | 6C15-30 | Abbott GmbH (Wiesbaden, Germany) |
| ARCHITECT CMV IgM Reagent Kit | 6C16-25 | Abbott GmbH (Wiesbaden, Germany) |
| ARCHITECT EBV VCA IgM Reagent Kit | 3P66-25 | Abbott GmbH (Wiesbaden, Germany) |
| ARCHITECT EBV EBNA-1 IgG Reagent Kit | 3P67-25 | Abbott GmbH (Wiesbaden, Germany) |

- 1. **Please provide the details of the corresponding methods author for the manuscript:**

| Keerthihan Thiyagarajah  Paul-Ehrlich-Institut  Federal Institute for Vaccines and Biomedicines  Staff Unit V/I  Paul-Ehrlich-Str. 51-59  63225 Langen  Phone  +49  6103  77 5419  E-Mail Keerthihan.Thiyagarajah@pei.de |
| --- |

**2.0 Please confirm for randomised controlled trials all versions of the clinical protocol are included in the submission. These will be published online as supplementary information.**

|  |
| --- |
