# Supplementary material for: Higher prevalence of cytomegalovirus and Epstein–Barr virus in acute-on-chronic liver failure
Source: JHEP Rep. 2025 Oct 9;8(4):101627. doi: 10.1016/j.jhepr.2025.101627 (PMC13019566; doi:10.1016/j.jhepr.2025.101627)
Supplement: Multimedia component 4 [file mmc4.pdf]

Research article

JHEP|Reports

Higher prevalence of cytomegalovirus and Epstein–Barr virus in acute-on-chronic liver failure

Authors

Keerthihan Thiyagarajah, Jannik Sonnenberg, Esra Görgülü, …, Tony Bruns, Eberhard Hildt, Kai-Henrik Peiffer

Correspondence

kai-henrik.peiffer@ukmuenster.de (K.-H. Peiffer).

Graphical abstract

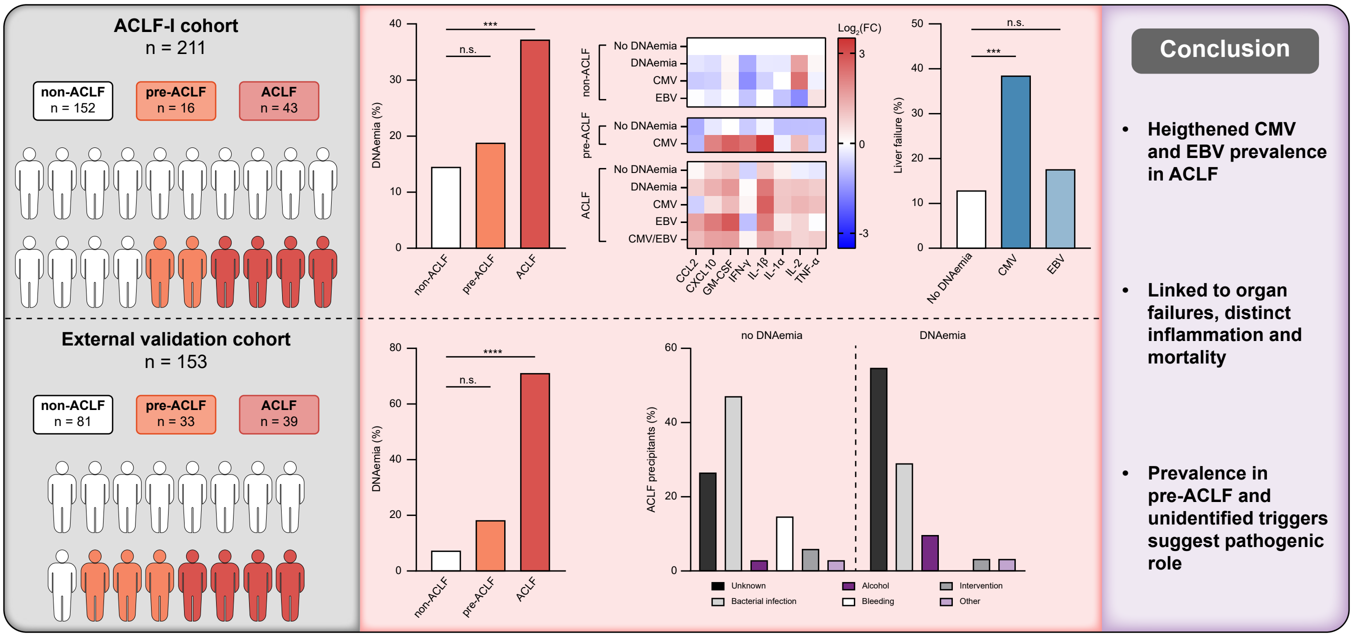

Highlights:

- CMV and EBV DNAemia are more frequent in patients with ACLF than in non-ACLF patients.
- DNAemia is associated with disease severity and a distinct inflammatory pattern.
- CMV correlates with liver failure, whereas EBV is associated with mechanical ventilation.
- Pre-ACLF is showing increased frequencies of CMV/EBV, although the results are not significant.
- CMV/EBV may be a trigger or sustainer of ACLF, but further research is needed to reach a definitive conclusion.

Impact and implications:

Despite ACLF being a life-threatening syndrome, the underlying precipitant cannot be identified in 30–40% of cases. As a result, intervention in ACLF development is limited to cases with known precipitants. To our knowledge, this is the first time it is shown that previously undiagnosed CMV and EBV DNAemia might act as a precipitating event inducing ACLF. We propose that routine screening and proper treatment for CMV and EBV in decompensated cirrhotic patients can intervene in ACLF development. This hypothesis needs to be assessed in prospective studies.

https://doi.org/10.1016/j.jhepr.2025.101627

© 2025 The Author(s). Published by Elsevier B.V. on behalf of European Association for the Study of the Liver (EASL). This is an open access article under the CC BY license (http://creativecommons.org/licenses/by/4.0/).

JHEP|Reports

Higher prevalence of cytomegalovirus and Epstein–Barr virus in acute-on-chronic liver failure

Keerthihan Thiyagarajah[1,2,†], **Jannik Sonnenberg**[3,†], Esra Görgülü[2], Pia Lembeck[3], Nico Kraus[2], Mirco Glitscher[1], Frank Erhard Uschner[3], Maximilian Joseph Brol[3], Wenyi Gu[3,4], Robert Schierwagen[3], Sabine Klein[3], Martin S. McCoy[3], Marcus Maximilian Mücke[2], Toska Wiedemann[2], Philipp A. Reuken[5], Johanna Reißing[6], Franziska Schneider[7], Nina Böhling[7], Michael Praktiknjo[3], Phil-Robin Tepasse[3], Julia Fischer[3], Stefan Zeuzem[2], Christoph Welsch[2], Sandra Ciesek[8], Andreas Stallmach[5], Jonel Trebicka[3], Johannes Chang[7], Tony Bruns[6], Eberhard Hildt[1,9], Kai-Henrik Peiffer[2,3,*]

JHEP Reports **2026**. vol. 8 | 1–12

Check for updates

Background & Aims: Acute-on-chronic liver failure (ACLF) is a life-threatening syndrome characterized by rapid deterioration of organ function in pre-existing chronic liver disease. Patients who develop ACLF within 90 days after decompensation are referred to as pre-ACLF. Known precipitants include bacterial infections and viral hepatitis. However, in 40–60% of patients, the precipitant remains unknown. Cytomegalovirus (CMV) and Epstein–Barr virus (EBV) are highly prevalent viruses, but their impact on ACLF is unclear.

Methods: 211 patients (43 ACLF, 16 pre-ACLF, and 152 non-ACLF) of the ACLF-I study and an external validation cohort with 153 patients (39 ACLF, 33 pre-ACLF, and 81 non-ACLF) were included. Sera were analyzed for CMV/EBV DNA (multiplex qPCR), cytokines in 102 ACLF-I samples (multiplex assays), and immunoglobulins in 80 case-control matched ACLF-I and 20 validation cohort patients (immunoassays), and correlated with clinical data.

Results: In the ACLF-I group, a higher prevalence of CMV DNAemia (8.9% *vs.* 25.6%, odds ratio [OR] 3.51, 95% CI 1.47–8.34, p <0.01) and EBV DNAemia (6.6% *vs.* 16.3%, OR 3.01, 95% CI 1.10–8.62, p <0.05) was observed in ACLF compared to non-ACLF cases, despite the absence of clinical signs of viral infections. 54.8% of DNAemic ACLF patients in the validation cohort had no identified precipitant compared to 26.5% in ACLF patients without DNAemia (p <0.05). CMV was associated with liver failure (p <0.001) and with 90-day mortality (p <0.001) in the regression model. DNAemia was associated with a distinct pattern of inflammatory activity. The results were validated externally. Serological analyses revealed that reactivation rather than primary infection occurred in most cases defined as DNAemic.

Conclusions: Presence of CMV/EBV DNAemia in chronic liver disease may contribute to the development of ACLF by exacerbating liver inflammation and impairing hepatocellular function.

Clinical trials registration: NCT04975490.

© 2025 The Author(s). Published by Elsevier B.V. on behalf of European Association for the Study of the Liver (EASL). This is an open access article under the CC BY license (http://creativecommons.org/licenses/by/4.0/).

Introduction

Liver cirrhosis and its complications were associated with 2.4% of global deaths in 2017, being prevalent in 112 million patients.[1] Acute-on-chronic liver failure (ACLF) is increasingly recognized as an important and distinct syndrome. Yet, multiple definitions are in use, generating a confusing situation for clinicians.[2,3] According to the EASL-Chronic Liver Failure Consortium (CLIF-C), it is characterized by the functional failure of one or more of six major organ systems (liver, kidney, brain, coagulation, circulation, and respiration) in patients with chronic liver disease.[4,5] It is associated with a 28-day mortality rate of at least 20% and up to 80% depending on the number of organ failures.[5] Known possible precipitants include systemic infections, invasive procedures, gastrointestinal hemorrhage, alcohol consumption, viral hepatitis, drug-induced liver injury, and autoimmune-related events.[6] Still, a precipitating event cannot be identified in 30–40% and the rate of unidentified precipitants exceeding 40% in the CANONIC study.[5] The distinctive spectrum of immune alterations associated with end-stage liver disease is called cirrhosis-associated immune dysfunction (CAID).[7] Affected patients are at an increased risk of developing clinically significant infections that are typically regarded as less pathogenic.

The EASL-CLIF-C defines pre-ACLF as the rapid development of ACLF within 90 days after decompensation of chronic liver disease.[8] The central hallmark of pre-ACLF is high systemic inflammation, as observed in the PREDICT study.[9] Over

* Corresponding author. Address: Medizinische Klinik B, University of Münster, Albert-Schweitzer-Campus 1, D-48149 Muenster, Germany. Tel.: +49 251 83 59689, +49 251 83 48288.
E-mail address: kai-henrik.peiffer@ukmuenster.de (K.-H. Peiffer).
† These authors share first authorship.
https://doi.org/10.1016/j.jhepr.2025.101627

the past few years, systemic inflammation has gained increasing attention as a predictor of ACLF, being more relevant for outcomes than circulatory dysfunction.[10] Systemic inflammation may impair the function of organ systems and cause immune-mediated tissue damage and metabolic changes.[11] It has recently even been shown to be a stronger predictor for bleeding than coagulopathy in acutely decompensated patients.[12]

Human herpesvirus (HHV) infections/reactivations are known precipitants of viral hepatitis and acute liver failure.[13,14] HHVs, like Cytomegalovirus (CMV) and Epstein–Barr virus (EBV), are highly prevalent, with 40–100% of populations being carriers of latent infections.[15,16] While in immunocompetent patients primary EBV infection has been shown to be a very rare cause of acute liver failure, CMV has not been convincingly linked to acute liver failure, to the best of our knowledge.[17,18] Yet, immunocompromised patients may develop fulminant hepatitis culminating in acute liver failure and the need for transplantation.[19,20] Furthermore, CMV and EBV have been described to be present in acutely decompensated liver cirrhosis and acute-on-chronic liver failure in case studies, lacking proof of a causal relation.[21] By modulating the innate immune system and suppressing host cell apoptosis, HHVs promote their own persistence, while active replication leads to increased cytokine production and immune cell activation.[22,23] As treatable factors, they may provide an opportunity to alleviate the clinical course of ACLF.

Therefore, this study aims to investigate the role of CMV and EBV in the context of ACLF. Patients with chronic liver disease from two cohorts from different German university hospitals were included and stratified regarding their disease trajectories as non-ACLF (compensated cirrhosis, stable decompensated cirrhosis/unstable decompensated cirrhosis), pre-ACLF, and ACLF. Findings from blood serum samples were subsequently correlated with clinical data gained throughout the treatment.

Materials and methods

Study design

In this retrospective analysis of the ongoing prospective longitudinal study Characterization and Pathogenesis of ACLF (ACLF-I, NCT04975490, Medical Faculty of Goethe University Frankfurt, No. 20-653) adult patients admitted to the Department of Internal Medicine I, University Hospital Frankfurt, Frankfurt, Germany, between 2021 and 2023 were included. Patients with liver cirrhosis who were admitted planned or unplanned were eligible for inclusion in the ACLF-I. Blood samples were collected at the time of inclusion. In total, 211 patients were included in this sub analysis of the ACLF-I, among these 43 presented with ACLF. Furthermore, 16 of the included patients developed ACLF within 90 days and were classified as pre-ACLF.

The following parameters were assessed for the ACLF-I patients: age, sex, aetiology of cirrhosis, ACLF/pre-ACLF, ACLF grade, 28-day and 90-day mortality, model for end-stage liver disease (MELD), MELD including sodium (MELDNa), Child-Pugh score, CLIF-C organ failure (OF) score, CLIF-C acute decompensation (AD) score, CLIF-C ACLF score, liver failure, renal failure, cerebral failure, coagulation failure, circulation failure, respiratory failure, sodium, potassium, creatinine, albumin, leucocytes, C-reactive protein (CRP), bilirubin, international normalized ratio (INR), aspartate aminotransferase (AST), alanine aminotransferase (ALT), alkaline phosphatase, gamma-GT, renal replacement treatment, ascites, hepatic encephalopathy, West Haven grade of hepatic encephalopathy, gastrointestinal hemorrhage, bacterial infections, viral infection, immunosuppression, medication, supplemental oxygen, and mechanical ventilation.

Data from two cohorts of patients from Germany with and without ACLF as well as pre-ACLF were included as an external validation cohort. The cohorts comprised 78 patients hospitalised for acute decompensation with ascites prior to puncture admitted to the Department of Internal Medicine III, University Hospital Aachen, Aachen, Germany, or the Department of Internal Medicine IV, Jena University Hospital, Jena, Germany, between 2010 and 2019 (33 non-ACLF, 7 pre-ACLF, 38 ACLF; Internal Review Boards: Jena University Hospital No. 683-02/3 and 2880-08/10; RWTH Aachen No. 327/19). A further 75 patients admitted to the Department of Medicine I, University Hospital Bonn, Bonn, Germany, between 2019 and 2023 with decompensated liver cirrhosis were included (49 non-ACLF and 26 pre-ACLF; Ethics review board No. 288/11, NCT04393519). Serum samples gathered at the time of inclusion were analysed retrospectively. Additionally, the ACLF precipitant was assessed. The pre-ACLF cohort from Bonn included the time to ACLF.

Written informed consent was obtained from each patient included in the study and the study protocols conform to the ethical guidelines of the 1975 Declaration of Helsinki, as reflected in the *a priori* approvals by the institutions' human research committees.

The diagnosis of cirrhosis was based on previous liver biopsy findings or a composite of clinical signs and findings provided by laboratory test results, endoscopy, and imaging. Diagnostic criteria for ACLF were based on the CANONIC study criteria.[5] Organ failure and organ dysfunction were defined according to the CLIF-C OF score, respectively failure of the liver, kidneys, brain, coagulation, circulatory, and respiratory system.[5]

Sample analysis

Samples within each center were handled and stored in the same way. After a blood sample was drawn, the samples were aliquoted and frozen at -80 °C within 2 h. Due to the process of aliquotation, freeze–thaw cycles were kept to a minimum prior to analysis and were identical within each cohort (see the supplementary data for further information).

CMV and EBV detection

Viral DNA extraction. Viral nucleic acids of blood sera were extracted using the High Pure Viral Nucleic Acid Kit (Roche Diagnostics, Mannheim, Germany) according to manufactureŕs instructions. Blood sera were previously spiked with Phocine herpesvirus (PhHV) DNA (Roche Diagnostics) to monitor extraction efficiency. 200 µl of blood sera was used as input and viral DNA was concentrated fourfold to a final concentration of 50 µl per sample during extraction.

Multiplex qPCR. Multiplex real-time quantitative PCR (qPCR) of extracted DNA samples was performed by using TaqMan hydrolysis probes (Roche Diagnostics) and the LightCycler Multiplex DNA master (Roche Diagnostics). 8 µl of master mix (Table S1) were mixed with 12 µl of extracted DNA sample to obtain a final reaction volume of 20 µl. The multiplex-qPCR was carried out in a Lightcycler 480 instrument II (Roche Diagnostics) following the recommended qPCR program for the respective TaqMan hydrolysis probes provided by the manufacturer. All samples were measured in duplicates.

Determination of serum viral loads. Multiplex-qPCR results were analysed using the Lightcycler 480 software. All qPCR data were colour compensated prior to analysis to correct results for crosstalk. Respective cycle threshold (Ct) values were determined by applying the Second Derivative maximum algorithm.

To determine the lower limit of detection (LLOD), a serial dilution ranging from 2 to 10^6 copies per reaction was performed. Seven copies per reaction was the lowest copy number detected in all replicates with more than 95% confidence. The calculated coefficient of variation of the Ct values (CV Ct) was 0.92% and 1.26% for CMV and EBV, respectively. Thus, seven copies per reaction was set as the LLOD. During each run, positive controls were run alongside to assess interassay variation. The interassay CV Ct was 1.69% and 0.94% for the CMV and EBV-positive controls throughout all runs. Respective Ct values were converted into total copy numbers using a standard curve ranging from 10 to 10^6 target molecules per reaction, representing the linear working range, as well as the lower and upper limit of quantification (LLOQ, ULOQ). The coefficient of determination for both standard curves was >0.99 (Fig. S1). Determined total copy numbers were translated into respective viral loads in blood serum using Equation 1:

$$\text{Viral load (copies / ml)} = (\text{total copies / reaction}) / (0.012 \text{ ml x } 4) \quad [1]$$

Accordingly, seven copies per reaction, presenting the LLOD, was equal to ~150 copies per ml blood serum. Hence, the cut-off value for samples to be considered as DNAemia for all clinical samples was set to 150 copies per ml for both viruses. The analysis was conducted in the same manner for samples from every cohort.

Cytokine analysis

Samples were processed within 2 h of collection by centrifugation at 2,000 × *g* for 10 min at 4 °C. The blood serum supernatant was aliquoted and stored at -80 °C until analysis. Haemolysed or lipemic samples were excluded from the study.

A custom multiplex magnetic bead panel (Luminex Discovery Assay Human Premixed Multi-Analyte Kit, Catalog No. LXSAHM-16, Luminex Corporation, Austin, TX, USA) was used to quantify inflammatory and immune-related cytokines. The assay was performed using the Luminex MAGPIX system according to the manufacturer's instructions. All samples were measured in duplicates.

Serum samples were thawed on ice and diluted 1:2 with assay buffer. Negative controls and internally standardised samples were included in every assay as quality controls and to adjust for background noise. Magnetic beads coated with specific capture antibodies were added to a 96-well plate and incubated with 50 µl of each sample for 2 h at room temperature under constant agitation. Following incubation, plates were washed three times using a magnetic plate washer to remove unbound components. Afterwards, biotinylated detection antibodies were added and incubated for 1 h at room temperature, followed by streptavidin–phycoerythrin conjugate incubation for 30 min. Following a final washing step, beads were resuspended in assay buffer and median fluorescence intensities were measured using the Luminex MAGPIX system.

Data were acquired using xPONENT software (Luminex Corporation) and subsequently analysed. Assay performance was assessed by intra-assay CV, with a threshold of <15% for acceptance. Samples with mean fluorescence intensity below zero after subtraction of analyte blanks were assigned a mean fluorescence intensity of zero. The detection limit was defined by a minimum of bound magnetic beads.

Serological analysis

Due to a lack of sample availability and volume, only a subpopulation was included in this analysis. Within the ACLF-I cohort, all cases of ACLF or DNAemia were included. However, the CMV DNAemic subgroup was limited to clinically relevant cases with CMV DNAemia above 600 copies/ml. Control samples were matched according to disease trajectory and severity scores, including all ACLF patients. Aditionally, pre-ACLF patients from the Bonn cohort were analysed using the same case-control approach. Anti-EBV and anti-CMV antibodies were assessed retrospectively using the Abbott EBV-VCA IgM, EBV EBNA-1 IgG, CMV IgM, and CMV IgG test kits on the Abbott Architect i1000SRTM platform (Abbott GmbH, Wiesbaden, Germany), according to the manufacturer's specifications.

Statistical analysis

Statistical analysis was performed using IBM SPSS Statistics for Windows, Version 29.0.0.0 (IBM Corporation, Armonk, NY, USA). Uni- and multivariable models were applied to identify correlations with CMV and EBV DNAemia and exclude possible confounding factors. In univariate statistical comparisons, tests against nominal variables were performed regarding the respective variable levels using Pearson Chi-square test for nominal variables, biserial correlation using Spearman-Rho coefficients for ordinal variables and *t* or Mann–Whitney *U* tests for metric variables. Whether normal distribution of metric variables could be assumed was tested by the Shapiro–Wilk test. Multiple linear regression using the backwards method was performed including variables with p <0.3 and variables of clinical relevance (see Table S2 for exact models). In all statistical analyses, significance was set at p <0.05. While initial analysis for virus prevalence were conducted including the full cohort, subgroup analysis for CMV and EBV were performed excluding the respective other group. Cases with missing data were excluded from the respective

analyses (Table S10). As the presented study is a sub-analysis of the ongoing ACLF-I clinical observation study, no sample size calculation to determine an adequate power to detect a prespecified effect size was performed.

Results

Patient characteristics

211 patients from the ACLF-I cohort with liver cirrhosis with or without ACLF were included. The cohort comprised 43 ACLF, 16 pre-ACLF and 152 non-ACLF patients. There were significant differences between the groups of patients with and without DNAemia of CMV or EBV regarding sex (26.5% *vs.* 46.3%, p = 0.013) and arterial hypertension (30.6% *vs.* 9.8%, p = 0.007) (data not shown). Immunosuppressive therapy showed no significant correlation with the presence of DNAemia (for sensitivity analyses, see Table S8). Detailed patient characteristics are depicted in Table 1. ACLF was present in 43 patients (20.4%, mean CLIF-C OF score, 10.1).

Higher prevalence of DNAemia in ACLF

With a threshold of 150 copies/ml, CMV was detected in ACLF and non-ACLF in a high percentage of patients (ACLF 48.8% *vs.* Non-ACLF 46.6%, p = 0.617). However, because the clinical relevance of CMV infection/reactivation correlates with viral load,[24] and to account for the high assay sensitivity, we further categorized the patients into groups with a higher and a lower DNA load (Fig. 1A,B). For further analysis, the group with >600 copies/ml was considered to have CMV DNAemia, while a lower DNA load was considered to be not clinically relevant (Fig. 1A).[25,26] Regarding EBV DNAemia, the initial threshold of 150 copies/ml resulted in a lower rate of detection than in CMV and a significant correlation with ACLF, and, therefore, was left unchanged.

Thereby, we identified 41 patients with CMV and/or EBV DNAemia within the ACLF-I collective (19.4%). 26 cases of CMV DNAemia (12.3%) and 17 cases of EBV DNAemia (8.1%) were identified with two patients presenting with both CMV and EBV. 16 cases of DNAemia could be attributed to the group of ACLF patients (37.2% *vs.* non-ACLF 14.5%; odds ratio [OR] 3.39; 95% CI, 1.60–7.18; p <0.001), respectively eleven cases of CMV (25.6% *vs.* non-ACLF 7.9%; OR 3.51; 95% CI, 1.47–8.34; p <0.01), and seven cases of EBV (16.3% *vs.* non-ACLF 6.6%; OR 3.01; 95% CI, 1.10–8.62; p <0.05). Two patients presented with CMV and EBV. In the pre-ACLF group, three patients tested positive for CMV (18.8% *vs.* non-ACLF 7.9%, p = 0.42) with no case of EBV (0% *vs.* non-ACLF 6.6%, p = 0.22) (Fig. 1C; all statistical analyses are detailed in Tables S2 and S3).

Serological prevalence of antibodies

In order to discriminate between primary infection and reactivation, serological analysis was performed to detect CMV and EBV-specific IgG and IgM. All patients with DNAemia and/or ACLF and matching controls were included. Presence of IgG for CMV (DNAemia 65% *vs.* no DNAemia 62.5%, p = 0.82) and EBV (EBNA-1 IgG DNAemia 95% *vs.* no DNAemia 95%, p = 1.00) did not differ significantly (Fig. 2B). CMV IgM were more frequent in the group with DNAemia yet not reaching significance (12.5% *vs.* 5%, p = 0.235), while EBV-VCA IgM

Table 1. Patient characteristics of the ACLF-I cohort.

Characteristic	No DNAemia	CMV and/or EBV DNAemia	*p* value
**N**	**170**	**41**	–
Age, years, mean ± SD	58.6 ± 12.0	55.5 ± 14.4	0.077
Female sex, n (%)	45 (26.5)	19 (46.3)	0.013
**Acute-on-chronic liver failure, n (%)**			
ACLF	27 (15.9)	16 (39.0)	<0.001
Pre-ACLF	13 (7.6)	3 (7.3)	0.943
Liver failure	22 (12.9)	12 (29.3)	0.011
Renal failure	16 (9.4)	5 (12.2)	0.593
Cerebral failure	6 (3.5)	5 (12.2)	0.025
Coagulation failure	11 (6.5)	4 (9.8)	0.462
Circulatory failure	18 (10.6)	6 (14.6)	0.472
Respiratory failure	5 (2.9)	3 (7.3)	0.188
**Etiology of cirrhosis, n (%)**			
Alcohol	88 (51.8)	25 (61.0)	0.288
MASH	21 (12.4)	4 (9.8)	0.644
Cholestatic liver disease	19 (11.2)	2 (4.9)	0.227
HCV	13 (7.6)	0 (0)	0.068
HBV (±HDV)	7 (4.1)	2 (4.9)	0.829
Autoimmune hepatitis	5 (2.9)	2 (4.9)	0.534
Other etiologies	17 (10.0)	6 (14.6)	0.393
**Laboratory values, median (IQR)**			
Creatinine, mg/L	1.13 (0.74)	1.14 (0.81)	0.997
Bilirubin, mg/dl	2.50 (4.80)	2.80 (15.20)	0.115
AST, U/L	61.0 (61.0)	80.50 (36.0)	0.066
ALT, U/L	34.0 (34.0)	33.50 (25.0)	0.792
INR	1.46 (0.72)	1.46 (0.53)	0.768
Albumin, g/dl	3.20 (1.0)	3.0 (0.70)	0.158
CRP, mg/L	1.74 (3.09)	3.40 (3.60)	0.068
White blood cell count, x 10^9/L	6.06 (5.04)	8.70 (8.0)	0.003
**Disease severity scores, median (IQR)**			
MELD	17 (11)	18 (15)	0.093
MELDNa	19 (12)	20 (17)	0.107
Child-Pugh score	9 (3)	9 (3)	0.147
CLIF-C ACLF	38 (12)	42 (14)	0.055
CLIF-C AD	60 (32)	71 (48)	0.069
CLIF-C OF	7 (2)	7 (4)	0.057
**Clinical data, n (%)**			
Diabetes mellitus	57 (33.5)	9 (22.0)	0.151
COPD	7 (4.1)	1 (2.4)	0.613
Heart failure	6 (3.5)	1 (2.4)	0.726
Arterial hypertension	52 (30.6)	4 (9.8)	0.007
Coronary artery disease	15 (8.8)	1 (2.4)	0.166
Chronic kidney disease	5 (2.9)	0 (0)	0.266
HCC	21 (12.4)	3 (7.3)	0.362
Immunosuppression*	14 (8.2)	5 (12.2)	0.299
In patients with ACLF	4 (2.4)	2 (4.9)	0.887
In patients with pre-ACLF	1 (0.6)	1 (2.4)	0.226
Ascites	100 (58.8)	29 (70.7)	0.160
Hepatic encephalopathy	40 (23.5)	14 (34.1)	0.162
Gastrointestinal hemorrhage	39 (22.9)	5 (12.2)	0.128
**Outcome, n (%)**			
28-day mortality	15 (8.8)	6 (14.6)	0.265
90-day mortality	33 (19.4)	8 (19.5)	0.988
Liver transplantation	12 (7.1)	1 (2.4)	0.269

The no DNAemia and CMV and/or EBV DNAemia subgroups of patients at the time of inclusion, organ failure definitions according to EASL CLIF-C (Pearson's Chi-square test, biserial rank-correlation, Mann-Whitney U test or t test depending on variable level). *Immunosuppressive regimes included azathioprine (n = 6), mycophenolate mofetil (n = 2) and corticosteroids (n = 12) with one patient receiving mycophenolate mofetil and corticosteroids (indications: autoimmune hepatitis [n = 10], systemic sclerosis [n = 1], rheumatoid arthritis [n = 1], Coombs-negative hemolysis [n = 1], and undocumented [n = 6]). ACLF, acute-on-chronic liver failure; AD, acute decompensation; ALT, alanine aminotransferase; AST, aspartate aminotransferase; CLIF-C, Chronic Liver Failure Consortium; CMV, cytomegalovirus; COPD, chronic obstructive pulmonary disease; CRP, C-reactive protein; EBV, Epstein–Barr virus; HCC, hepatocellular carcinoma; INR, international normalized ratio; MASH, metabolic-associated steatohepatitis; MELD, model of end-stage liver disease; MELDNa, model of end-stage liver disease including sodium; OF, organ failure.

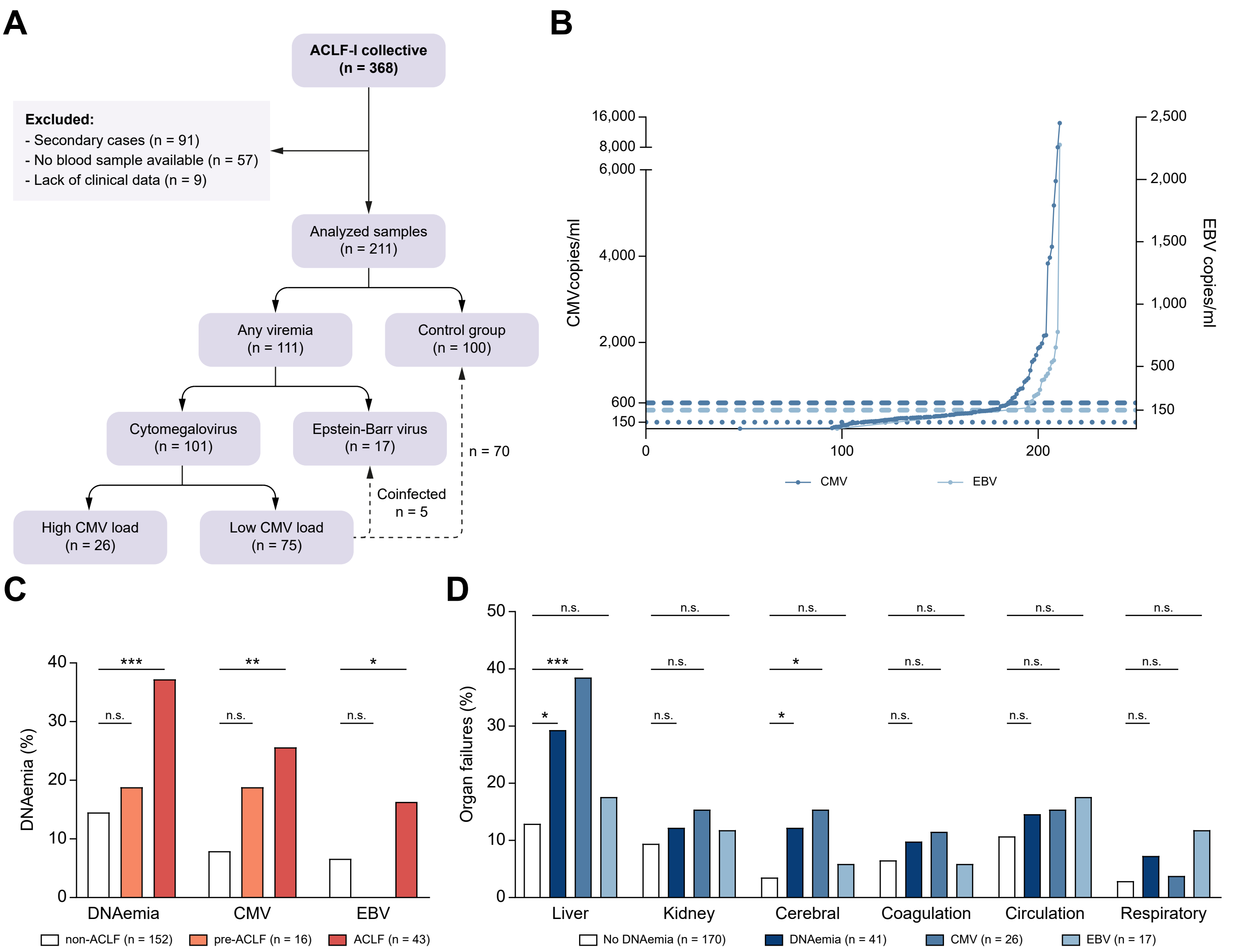

Fig. 1. ACLF-I patient collective, DNAemia, and organ failures. (A) Flow chart of ACLF-I patient selection. High CMV load >600 copies/ml, low CMV load 150–600 copies/ml. (B) Copies/ml of CMV and EBV, cases ranked ordinally. Dotted line represents the initial CMV 150 copies/ml cut-off, dashed lines represent cut-offs for samples considered as DNAemia (CMV 600 copies/ml; EBV 150 copies/ml). (C) Distribution of DNAemia in the ACLF-I collective (Pearson's Chi-square test: n.s., *p* >0.05, **p* <0.05, ***p* <0.01, ****p* <0.001). (D) Organ failures in the ACLF-I collective according to the CLIF-C ACLF score (Pearson's Chi-square test: n.s., *p* >0.05, **p* <0.05, ***p* <0.01, ****p* <0.001).

were borderline significantly more frequent in those patients defined as DNAemic in Pearson Chi-square test (15.0% *vs.* 2.5%, *p* = 0.048); this could not be reproduced in the logistic regression model (OR 6.88; 95% CI, 0.79–60.06; *p* = 0.08). These data indicate that reactivation rather than primary infection occurred in the vast majority of cases defined as DNAemic. Interestingly, CMV IgG prevalence was significantly higher in the ACLF group compared to non-ACLF group (80.0% *vs.* 55.0%; OR 3.27; 95% CI, 1.03–10.45; *p* <0.05).

Influence of sex on EBV

In the presented cohort, a significant difference in EBV DNAemia between male and female patients was found. EBV DNA was detected in sera from 16.7% of females compared to 6.0% from male patients (*p* <0.05). This correlation was confirmed in the EBV regression analysis with sex as independent influence factor. Within the CMV group no association with sex was found..

CMV is associated with liver failure and EBV with mechanical ventilation

CMV DNAemia was significantly associated with liver failure (bilirubin >12 mg/dl, *p* <0.01) and cerebral failure (West Haven Criteria grade 3 or 4, *p* <0.05) (Fig. 1D). The observed association with liver failure was additionally supported by a correlation with increased bilirubin, AST, alkaline phosphatase, and gamma-GT levels in the CMV group. EBV showed no significant correlation with a singular organ failure on univariate analysis. Yet, need for vasopressors for circulation failure and mechanical ventilation increased the risk for EBV DNAemia more than seven times. Consequently, the EBV regression

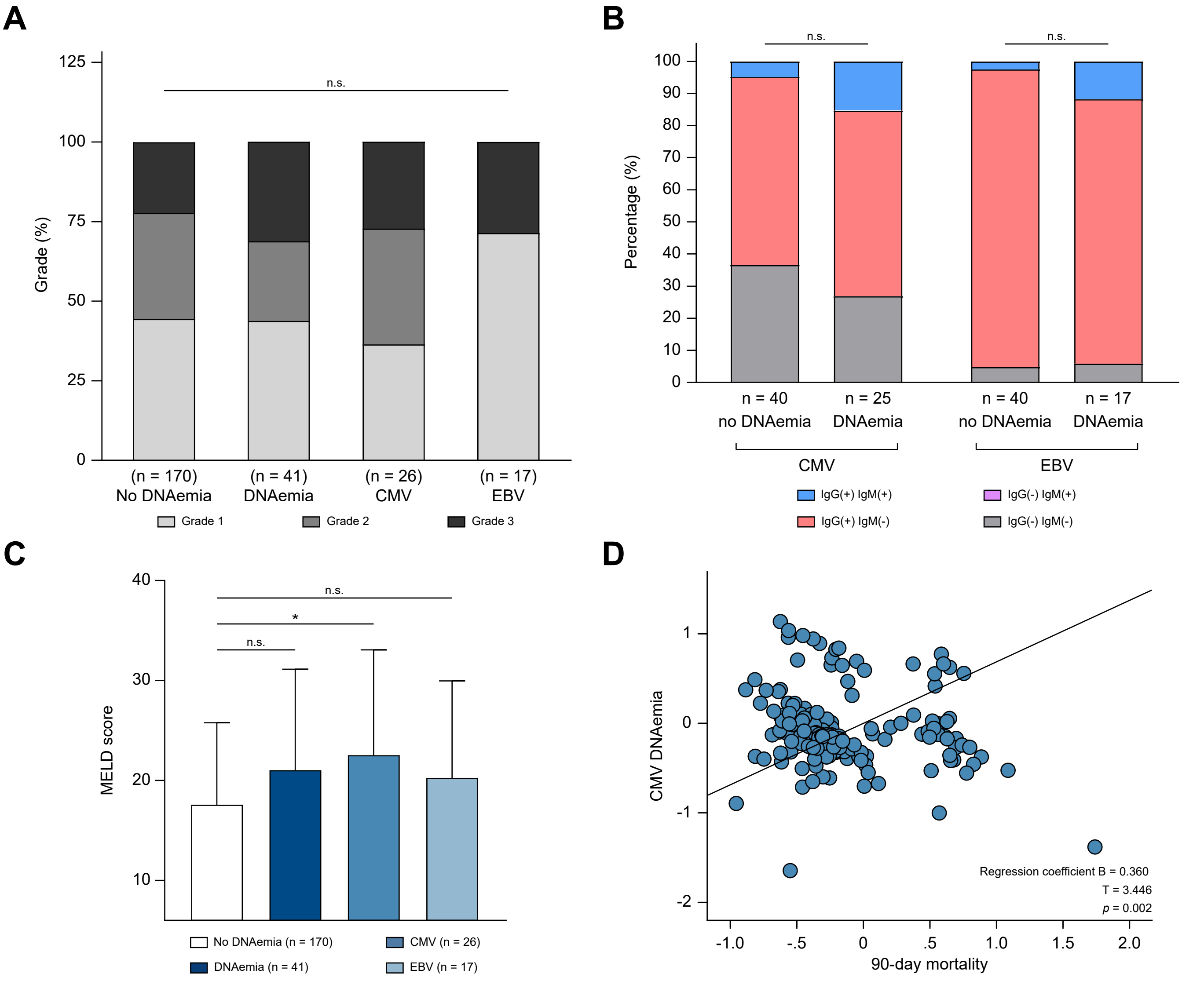

Fig. 2. ACLF grade, serological data, MELD score, and mortality in the ACLF-I collective. (A) ACLF grades in the ACLF-I collective (individual tests Pearson's chi-squared test, overarching test biserial correlation using Spearman-Rho coefficients: n.s.*p* >0.05, **p* <0.05, ****p* <0.001) (B) Prevalence of CMV and EBV antibodies in a case-control group of patients with DNAemia matched with non DNAemic patients. (C) MELD score in points (average, SD) in the ACLF-I cohort (Biserial rank-correlation: n.s.*p* >0.05, **p* <0.05). (D) Partial regression diagram of the independent variable 90-day mortality on the CMV DNAemia group in the ACLF-I collective, including the fitting line with suppressed intersection (Multiple linear regression, backward elimination method, *p* = 0.002).

analyses showed a significant independent influence for mechanical ventilation (no DNAemia 7.3% *vs.* DNAemia 33.3%).

Use of vasopressors (*p* <0.01) and mechanical ventilation (*p* <0.05) increased the risk for EBV DNAemia in the ACLF-I cohort. Aditionally, regression showed significantly reduced DNAemia in patients with gastrointestinal hemorrhage (hemorrhage 11.4% *vs.* no hemorrhage 21.6%). The ACLF grade was not significantly impacted by the presence of DNAemia (Fig. 2A). Patients testing positive for CMV displayed higher CLIF-C OF (mean 7.3 *vs.* 8.4, respectively; *p* <0.05) and CLIF-C AD (mean 63.4 *vs.* 76.6, respectively; *p* <0.05) scores as expression of the the acute disease severity. Additionally, the MELD score was significantly higher in patients with CMV (mean 17.6 *vs.* 22.6, respectively; *p* <0.05) (Fig. 2C).

Mortality

Mortality was included in the regression models due to the clinical relevance, although it did not significantly correlate with CMV and/or EBV DNAemia in univariate analysis. It was found that 28- and 90-day mortality was significantly associated with CMV DNAemia in separate models (Fig. 2D). For EBV, a significant correlation for 90-day mortality was found in the regression analyses, while 28-day mortality was included in the respective model but did not reach significance.

Inflammation increases in DNAemia

Within both the CMV and EBV DNAemia groups, higher leukocyte levels as markers of inflammation correlated with

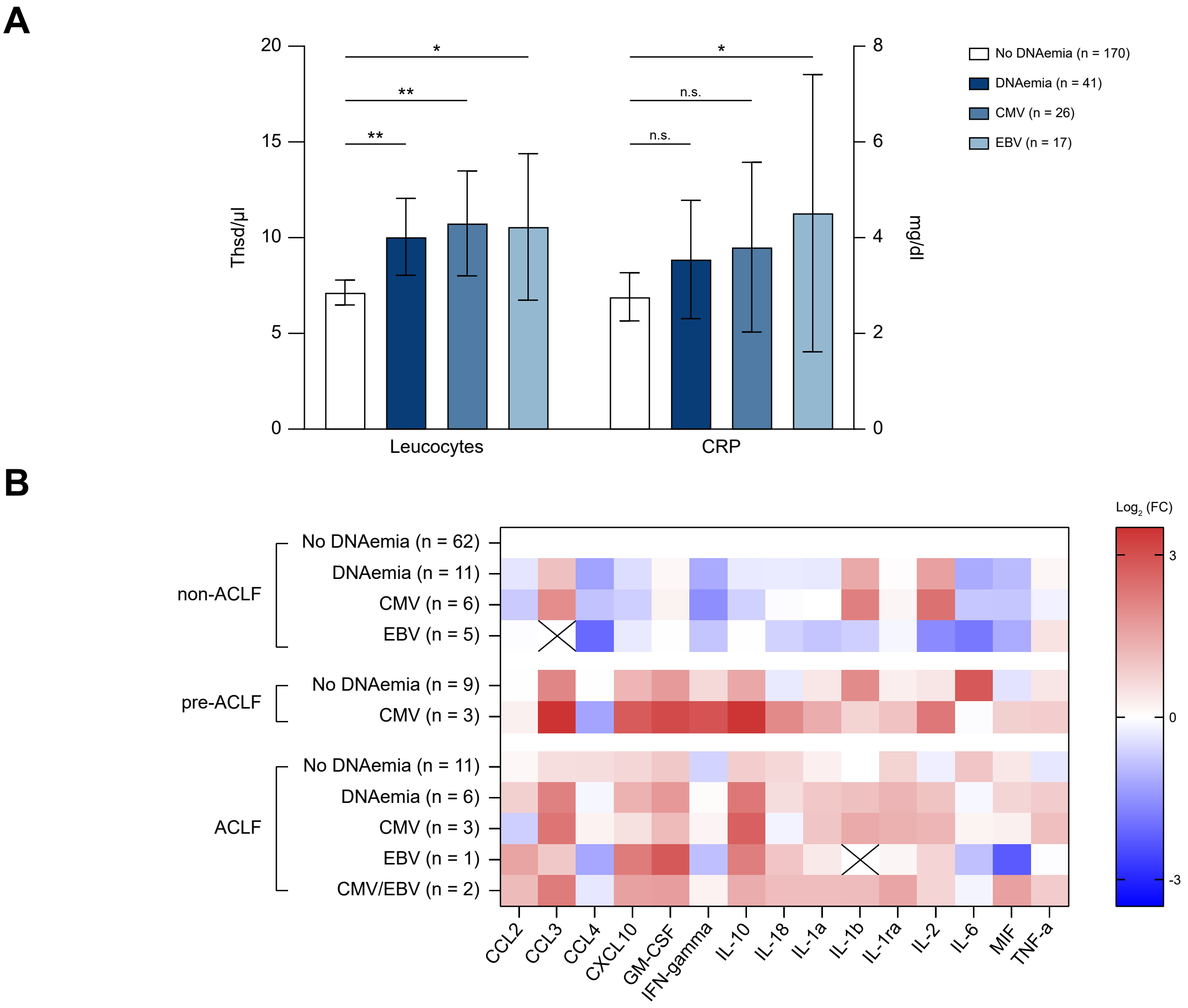

Fig. 3. Inflammation in the ACLF-I collective. (A) Mean serum leucocyte numbers and CRP levels (bars represent SD) in the ACLF-I collective (Mann-Whitney-U-test: $^{n.s.}p$ >0.05, *p <0.05, **p <0.01). (B) Log_2-transformed fold changes of cytokine levels. Results of statistical analyses can be found in Table S6. CCL, CC-chemokine ligand; CXCL, C-X-C motif chemokine ligand; GM-CSF, granulocyte-macrophage colony-stimulating factor; IFN, interferon; MIF, macrophage migration inhibitory factor; TNF-α, tumor necrosis factor α.

increased risk for DNAemia (CMV U = 1,440; z = -2.83; p <0.01; EBV U = 980; z = -2.16; p <0.05) (Fig. 3A). They gained additional significance as independent influencing factors in regression analysis for EBV. In parallel, there were higher levels of CRP in patients with EBV compared with those without (U = 757; z = -2.01; p <0.05). Furthermore, in the CMV group, CRP gained borderline significance in the regression models as an independent predictor (p <0.05).

Further exploring possible mechanisms leading to the above-described clinical findings, multiplex cytokine assays of patient blood samples were performed. A subpopulation of 102 patients of the ACLF-I collective was analysed for cytokine profiles, due to sample availability (Fig. 3B). Stratification for ACLF and DNAemia of this subgroup showed overall reduced cytokine levels in non-ACLF patients when presenting relevant CMV and/or EBV DNAemia. This switches towards an increase in cytokine levels in pre-ACLF patients and ACLF patients when additionally presenting DNAemia. Furthermore, the type-I interferon (IFN) inducible chemokine C-X-C motif chemokine ligand (CXCL)-10 was significantly upregulated in patients with CMV and/or EBV DNAemia (U = 48; z = -2.98; p <0.01). Yet, within ACLF patients with DNAemia, levels of IFN-γ and CC-chemokine ligand (CCL4) remained unchanged. Furthermore, patients with CMV DNAemia showed higher levels of IL-10 in pre-ACLF (p = 0.15) and ACLF (U = 17; z = -2.39; p <0.05) (Table S5).

External validation

A cohort of 153 patients admitted to the University hospitals in Aachen, Jena, and Bonn with acute decompensation of cirrhosis with and without ACLF was included to validate the results. Further focus for this cohort was laid on the inclusion of pre-ACLF patients, asonly a relatively small group with this trajectory was included in the ACLF-I. Details on patient characteristics can be found in Table 2 (for statistical analysis of the two individual cohorts, see Tables S6 and S7).

Prevalence of DNAemia in pre-ACLF and ACLF

We found 39 cases of DNAemia within the validation cohort (25.5%), with 35 cases of CMV DNAemia (22.9%) and 20 cases of EBV DNAemia (13.1%) identified (Table S4). Respectively,

Table 2. Patient characteristics of the validation cohort.

Characteristic	No DNAemia	CMV and/or EBV DNAemia	*p* value
**N**	**114**	**39**	–
Age, years, mean ± SD	60.6 ± 9.9	60.3 ± 10.8	0.435
Female sex, n (%)	41 (36.0)	8 (20.5)	0.074
**Acute-on-chronic liver failure, n (%)**			
ACLF	12 (10.5)	27 (69.2)	<0.001
Pre-ACLF	27 (23.7)	6 (15.4)	0.089
Liver failure	1 (0.9)	3 (7.7)	0.021
Renal failure	12 (10.5)	27 (69.2)	<0.001
Cerebral failure	6 (5.3)	0 (0)	0.144
**Etiology of cirrhosis, n (%)**			
Alcohol	92 (80.7)	27 (69.2)	0.137
MASH	7 (6.1)	2 (5.1)	0.817
Cholestatic liver disease	3 (2.6)	1 (2.6)	0.982
HCV	3 (2.6)	1 (2.6)	0.982
HBV	1 (0.9)	2 (5.1)	0.098
Autoimmune hepatitis	1 (0.9)	0 (0)	0.557
Other etiologies	7 (6.1)	6 (15.4)	0.074
**Laboratory values, median (IQR)**			
Creatinine, mg/L	1.07 (0.71)	2.55 (2.82)	<0.001
Bilirubin, mg/dl	1.28 (1.30)	1.52 (2.40)	0.418
AST, U/L	57 (53)	79.5 (80)	0.031
ALT, U/L	33 (34)	44 (44)	0.301
INR	1.3 (0.2)	1.4 (0.5)	0.042
Albumin, g/dl	2.9 (0.8)	2.7 (0.9)	0.612
CRP, mg/L	1.62 (1.56)	2.56 (2.68)	0.065
White blood cell count, x 10^9/L	6.84 (4.52)	6.95 (5.60)	0.943
**Disease severity scores, median (IQR)**			
MELD	12 (6)	22 (13)	<0.001
CLIF-C ACLF	48 (6)	44 (11)	0.320
CLIF-C AD	52 (8)	51 (14)	0.960
CLIF-C OF	6 (0)	7 (2)	<0.001
**Clinical data, n (%)**			
Diabetes mellitus	33 (28.9)	19 (48.7)	0.024
COPD	11 (9.6)	1 (2.6)	0.155
Heart failure	14 (12.3)	5 (12.8)	0.930
Arterial hypertension	33 (28.9)	19 (48.7)	0.024
Coronary artery disease	11 (9.6)	4 (10.3)	0.912
Chronic kidney disease	16 (14.0)	8 (20.5)	0.337
HCC	6 (5.3)	3 (7.7)	0.578
Immunosuppression*	1 (0.9)	0 (0)	0.557
In patients with pre-ACLF	1 (0.9)	0 (0)	0.632
**Outcome, n (%)**			
28-day mortality	19 (16.7)	6 (15.4)	0.852
90-day mortality	31 (27.2)	10 (25.6)	0.850

The no DNAemia and CMV and/or EBV DNAemia subgroups of patients at the time of inclusion, organ failure definitions according to EASL CLIF-C (Pearson‘s Chi-square test, biserial rank-correlation, Mann-Whitney *U* test or *t* test depending on variable level). *Immunosuppressive regime with mycophenolic acid and everolimus due to heart transplantation. ACLF, acute-on-chronic liver failure; AD, acute decompensation; ALT, alanine aminotransferase; AST, aspartate aminotransferase; CLIF-C, Chronic Liver Failure Consortium; CMV, cytomegalovirus; COPD, chronic obstructive pulmonary disease; CRP, C-reactive protein; EBV, Epstein–Barr virus; HCC, hepatocellular carcinoma; INR, international normalized ratio; MASH, metabolic-associated steatohepatitis; MELD, model of end-stage liver disease; MELDNa, model of end-stage liver disease including sodium; OF, organ failure.

16 patients were presenting with CMV and EBV. 26 cases of CMV (66.7% *vs.* non-ACLF 4.9%; OR 40.6; 95% CI, 12.0–137.1; p <0.00001) and 15 cases of EBV (38.5% *vs.* non-ACLF 2.5%; OR 46.9; 95% CI, 9.5–231.3; p <0.00001) could be attributed to the ACLF group. In the pre-ACLF group, three cases of EBV DNAemia (9.1%; OR 4.0; 95% CI, 0.6–25.1; p = 0.11) and five cases of CMV DNAemia (15.2%; OR 3.5; 95% CI, 0.9–13.9; p = 0.06) were identified (Fig. 4A) (see Tables S2 and S4 for all statistical analyses). Serological results revealed a similar picture to the ACLF-I cohort, showing no significant differences in IgG (CMV IgG DNAemia 66.7% *vs.* no DNAemia 64.3%; EBV EBNA-1 IgG DNAemia 66.7% *vs.* no DNAemia 100%) and CMV IgM (DNAemia 16.7% *vs.* no DNAemia 0%) (data not shown).

Disease severity and DNAemia

Congruently to the ACLF-I cohort, DNAemia correlated strongly with MELD score (CMV r = 0.679; p <0.0001; EBV r = 0.699; p <0.0001; Fig. 4B). No impact on the ACLF grade was identified. Furthermore, a higher CLIF-C ACLF score (r = 0.922; p <0.01) correlated with EBV DNAemia. A direct comparison between the cohorts revealed lower ACLF grades in the validation cohort compared to the ACLF-I (p <0.0001).

Within the validation cohort, correlations could be identified for DNAemia with ACLF and especially renal failure, with every ACLF patient in this cohort presenting this organ failure. Consequently, higher creatinine similarly showed a strong correlation (p <0.00001) and was included in the regression models as a significant influence factor. Congruent results were found in the subgroups of CMV and EBV. In parallel to ACLF-I, CMV DNAemia showed a significant correlation with liver failure (OR 10.6; 95% CI, 1.1–105.3; p <0.05). In addition, increased INR correlated with CMV (U = 1,480.5; z = -2.33; p <0.05).

Inflammation and mortality in DNAemia

In this cohort, no significant association of CRP and leukocytes with CMV and/or EBV DNAemia could be found. Immunosuppressive therapy showed no significant correlation with the presence of DNAemia, as in the ACLF-I cohort (see Table S9 for sensitivity analyses).

Sex did not have a significant impact on CMV and/or EBV DNAemia or CMV DNAemia alone in the validation cohort. Yet, male patients were affected by EBV DNAemia significantly more frequently (17.3% *vs.* females 4.1%, p <0.05). Sex did gain significance in the regression models for CMV.

In parallel to the ACLF-I cohort, no significant difference in mortality of the groups could be identified on univariable analysis (Fig. 4D). Regression analysis yielded models including 28-day and 90-day mortality as significant influence factors for CMV in separate models.

ACLF precipitants and DNAemia

For the validation cohort documentation of ACLF precipitants was available (Fig. 4C). Analysis showed significant differences between the groups with and without DNAemia (p <0.05) as bacterial infections dominated in the no DNAemia group as most frequent precipitant (47.1% *vs.* DNAemia 29.0%, p = 0.14), while most patients within the DNAemia group had an unknown precipitant (54.8% *vs.* no DNAemia 26.5%; OR 3.37; 95% CI, 1.19–9.54; p <0.05).

Discussion

ACLF is a severe clinical syndrome, being associated with mortality rates of up to 80%.[5] Although infection and reactivation of CMV and EBV are frequent in immune dysfunction, a correlation with ACLF has so far not been investigated.

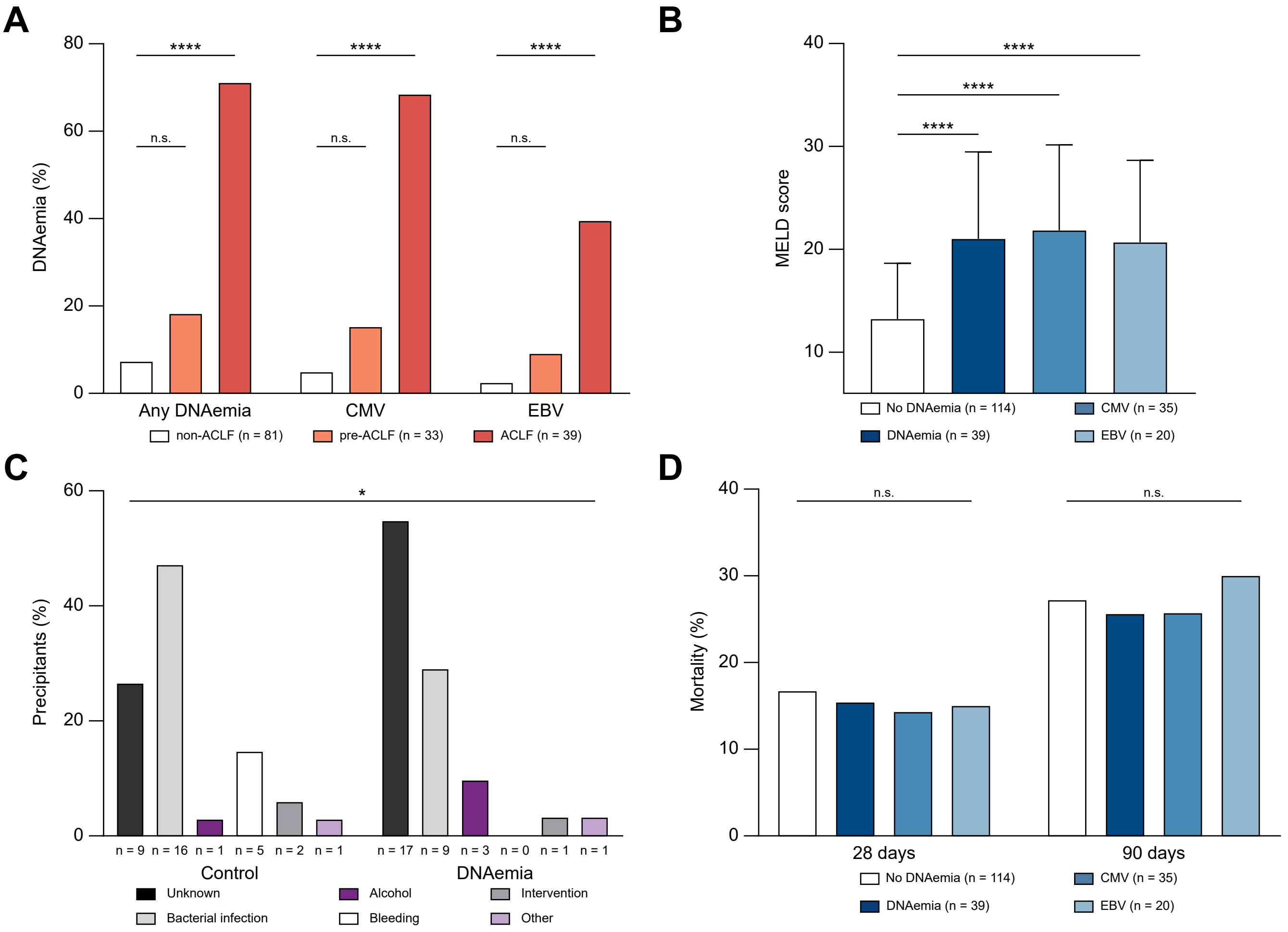

Fig. 4. DNAemia, precipitants of ACLF and severity of disease in the validation cohort. (A) Distribution of CMV and EBV DNAemia over the validation cohort (Pearson's chi-squared test: $^{n.s.}p$ >0.05, $^{****}p$ <0.0001). (B) MELD score in points (average, SD) in the validation cohort (Biserial rank-correlation: $^{****}p$ <0.0001). (C) ACLF precipitants in the validation cohort (Pearson's chi-squared test: $^{*}p$ <0.05). (D) Mortality in the validation cohort (Pearson's chi-squared test: $^{n.s.}p$ >0.05).

The first important finding of the present study is the presence of CMV and EBV DNAemia in significantly higher frequency in ACLF patients, which could be validated in the validation cohort. Reactivation in patients who are critically ill is frequent and can occur in 6 and up to 55% of patients in intensive care units.[27,28] Therefore, our identified rates of DNAemia of 19.4% and 25.5% are therefore in line with prior publications regarding critically ill patients. The serological analysis of immunoglobulins suggests that, with IgG present in 65% of CMV and 100% of EBV cases, reactivations of previous infections occurred as expected when considering the high seroprevalence of these viruses in adult populations. Whilefew patients with CMV DNAemia did not express any immunoglobulins, this may be caused by CAID and does not necessarily infer primary infection. The difference in CMV IgG between ACLF and non-ACLF, albeit interesting, may be unspecific as IgM showed no such difference.

Yet, clinically relevant HHV infection is considered an issue attributed mainly to immunosuppression, neoplasia, and critically ill patients and reactivation is debated wheter to impact outcomes in intensive care unit cohorts.[29,30] Therefore, diagnostics and treatment of HHV reactivation is currently no standard procedure in intensive care units.[31] In the presented cohorts, DNAemia correlated with possible consequences of infections, such as liver failure for CMV, mechanical ventilation and the need for vasopressors for EBV.[28] The associations with organ failures and mortality may reflect more advanced disease and immune dysfunction, as the single time point analysed does not allow to infer causality.

Besides, as inflammation is gaining increasing attention as an important factor in ACLF,[32] we found significant correlations between CMV and EBV prevalence and inflammatory markers, such as leucocytes and CRP, in the ACLF-I cohort. For the immunosuppressive therapies present in the cohorts (*e.g.* because of autoimmune hepatitis), no correlation with DNAemia was found and sensitivity analyses revealed no contradicting results (Tables S8 and S9). Further cytokine analysis showed overall reduced cytokine levels in non-ACLF but with DNAemia. This footprint switched toward an increased cytokine expression in pre-ACLF and ACLF patients, in line with previously described characteristics of inflammation in ACLF.[12] However, IFN-γ levels remained relatively low

to unchanged in the ACLF DNAemia groups. Detection of low IFN-γ levels is in accordance with a recent study describing decreasing levels in patients with acutely decompensated cirrhosis as associated with progression of organ failures and posing a risk factor for the development of ACLF.[33] Furthermore, IFN-γ is reduced in patients affected by immunosuppressive therapies and CAID.[34,35] IFN-stimulated genes in peripheral blood mononuclear cells of patients with alcoholic cirrhosis are also known to show decreased constitutive expression and lower induction.[36] Therefore, patients with lower IFN-γ levels may be at risk of developing CMV/EBV DNAemia.[37] Since we did not observe a correlation between DNAemia and immunosuppressive therapies, the relatively reduced levels in ACLF patients maypredispose this group for CMV/EBV DNAemia. Our findings further included increased IL-10 levels in CMV and EBV positive patients with pre-ACLF and ACLF, which is part of the viral immunoevasive mechanisms.[38] The increase in IL-10 may be of clinical importance due to weakening effects on the immune system. The elevated levels of the IFN-induced chemokine CXCL10 in patients with CMV and/or EBV DNAemia indicate a virally driven surge in type-I IFN production. In the context of cirrhosis, type-I IFNs act on myeloid cells, thereby priming subsequent inflammatory responses and enhancing susceptibility to inflammatory caspase activation and inflammasome assembly, driving inflammation and organ failure in cirrhosis.[39] Therefore, the cytokine data may support a potential role of CMV/EBV as a precipitant or sustainer of ACLF, depending on the time of DNAemia development. However, longitudinal data are needed to further clarify the viral contribution to these nonspecific markers and distinguish the findings from the systemic inflammation intrinsic to ACLF.

Recently, associations with mortality in patients critically ill with COVID-19 have been published, supporting our findings of mortality being associated with CMV/EBV DNAemia.[29] However, although it has been frequently reported that HHV reactivation can alter patients' prognoses, a causal link remains to be proven.

Therefore, we also investigated patients with pre-ACLF and precipitating events to determine whether CMV and/or EBV DNAemia is a cause or consequence of ACLF. Given that only a few patients in the ACLF-I cohort presented with pre-ACLF, a validation cohort from Bonn, including 26 pre-ACLF 49 non-ACLF, was incorporated in this study. In the past, few studies have evaluated the impact of CMV and/or EBV DNAemia on ACLF and liver function, especially in terms of the possible triggering effect.[40,41] Given that use of non-standardized diagnostic criteria for ACLF and lack of validation, the results were not transferable. In this study, we used criteria established by the EASL-CLIF-C for the diagnosis of ACLF.

In all cohorts and in nearly every subgroup, the frequency of DNAemia increased steadily from non-ACLF over pre-ACLF to ACLF, although not reaching significance in patients with pre-ACLF in the ACLF-I and validation cohorts (Figs 1C and 4A). Further analysis of the documented ACLF precipitants identified a gap of known triggers in the validation DNAemia groups. Given that CMV and EBV infections are known triggers for systemic inflammation and can cause cytokine production and persistent immune cell activation, they might act as extrahepatic precipitants for the development of ACLF.[42] Intrahepatic insults are also possible in the form of CMV hepatitis, as observed in this study, given that CMV was associated with liver failure.[43] In addition, as a known precipitant of ACLF, gastrointestinal hemorrhage was associated with a lower risk for DNAemia. However, future studies are required to investigate this trend further.

In the ACLF-I cohort, serological and/or PCR screening for CMV and EBV infection/reactivation during treatment was performed in only 6% of patients and no case of retrospectively identified CMV and/or EBV DNAemia was identified throughout the treatment period. No clinical signs of CMV or EBV infections were documented. Trials conducted of treatment of CMV reactivation in patients who were critically ill have thus far reported negative results.[44–46] However, these results are not transferable to ACLF collectives and the situation might be different in patients with ACLF, given that CMV and EBV infection/replication might act as the underlying precipitant of ACLF. A hallmark and sustainer of ACLF is continuous inflammation, presenting an additional characteristic not similarly relevant in patients in general intensive care units.[10,47] Furthermore, the prospective identification of patients with pre-ACLF being investigated in studies such as MICROB-PREDICT could offer treatment opportunities not available for other syndromes. However, the definitive identification of the clinical significance of CMV and EBV reactivation for outcomes, screening, and identification of patients at high risk of developing unfavorable clinical courses and, therefore, in need of prompt treatment, should be evaluated in comparable further studies.

Taken together, our data describe a correlation between DNAemia and ACLF, suggesting DNAemia as prognostic marker for ACLF. However, because of the retrospective nature of our study, no definitive causal relationship between DNAemia and ACLF can be established. Accordingly, prospective analyses of patient material and correlations with the clinical situation is needed in further studies.

Another limitation of our study is that, because of the availability of samples in this retrospective analysis of prospective trials, blood serum samples were used for analysis. Aside from higher sensitivity for detection of CMV, previous studies have shown that CMV DNA can be up to 10-fold higher in whole blood compared with serum samples, likely because of the detection of intracellular viruses.[48,49] In addition, different commercial reagents and laboratory-developed tests have shown up to a 3 $\log_{10}$ difference in CMV viral load detection.[50] Therefore, the above-mentioned LLOD and thresholds regarding considerations of results as relevant DNAemia can not be directly translated into clinically used international units. Furthermore, the threshold for CMV copies was derived from previous publications on transplant recipients and has not been prospectively validated in non-solid organ transplant recipients.[25,26] Thus, this might underestimate the prevalence and viral load, which could affect classifications and associations.

A strength of this study is the external validation, which was conducted with patient cohorts recruited in two different German research projects. Aside from the external validation and increased heterogeneity, the burden of disease was

distributed differently from the ACLF-I cohort, presenting significantly lower ACLF grades compared to the ACLF-I cohort. With lower ACLF grades, these patients might exhibit less immune dysfunction than found in critically ill cohorts and the impact of CAID might also be lower. The differences in terms of organ failures between the cohorts can be explained by the aetiologies of ACLF, with the ACLF-I cohort presenting higher frequencies of liver failure and the Aachen/Jena cohort exclusively presenting ACLFs including renal failure. Therefore, the validation cohort provides valuable insights, given that renal failure ranks among the most frequent organ failures found in ACLF and was underrepresented in the ACLF-I cohort (27.9%).[5]

Conclusions

Our data show that CMV and EBV DNAemia is associated with ACLF, the severity of disease, the underlying stage of liver cirrhosis, mortality, and a distinct pattern of inflammation. Reactivation/infection with these viruses could contribute to development/aggravation of the syndrome by exacerbating liver inflammation and impairing hepatocellular function. Our data also suggest that CMV and EBV DNAemia act as potentially treatable precipitants of ACLF. However, further studies are needed to evaluate the role of these viruses and antiviral treatments in the context of ACLF.

Affiliations

[1]Paul-Ehrlich-Institut, Langen, Germany; [2]Goethe University Frankfurt, Medical Clinic 1, University Hospital, Frankfurt am Main, Germany; [3]Medizinische Klinik B, University of Münster, Münster, Germany; [4]Department of Gastroenterology, Ren Ji Hospital, School of Medicine, Shanghai Jiao Tong University, Shanghai, China; [5]Klinik für Innere Medizin IV, Jena University Hospital, Friedrich Schiller University Jena, Jena, Germany; [6]Medizinische Klinik III, RWTH Aachen University, Aachen, Germany; [7]Department of Internal Medicine I, University Hospital Bonn, Bonn, Germany; [8]Institut für Medizinische Virologie, Goethe University Frankfurt, Frankfurt, Germany; [9]Hasso-Plattner-Institute-Digital Health Cluster, University Potsdam, Potsdam, Germany

Abbreviations

ACLF, acute-on-chronic liver failure; AD, acute decompensation; ALT, alanine aminotransferase; AST, aspartate aminotransferase; CAID, cirrhosis-associated immune dysfunction; CCL, CC-chemokine ligand; CLIF-C, Chronic Liver Failure Consortium; CMV, cytomegalovirus; COPD, chronic obstructive pulmonary disease; CRP, C-reactive protein; Ct, cycle threshold; CV, coefficient of variation; CXCL, C-X-C motif chemokine ligand; EBV, Epstein–Barr virus; GM-CSF, granulocyte-macrophage colony-stimulating factor; HCC, hepatocellular carcinoma; HHV, human herpesvirus; IFN, interferon; INR, international normalized ratio; LLOD, lower limit of detection; MASH, metabolic-associated steatohepatitis; MELD, model of end-stage liver disease; MELDNa, model of end-stage liver disease including sodium; MIF, macrophage migration inhibitory factor; OF, organ failure; OR, odds ratio; PhHV, Phocine herpesvirus; qPCR, real-time quantitative PCR; TNF-α, tumor necrosis factor α.

Financial support

This work was funded by the State Hesse, Germany ('Landes-Offensive zur Entwicklung Wissenschaftlich-ökonomischer Exzellenz', ACLF-I, project P7), the Deutsche Forschungsgemeinschaft (DFG, German Research Foundation; 493624047), Clinician Scientist Careers Münster (322274963), SFB1382 Project ID 403224013/B07, Clinician Scientist Program Münster No. 2024_002, and the Advanced Clinician Scientist Program (ACCENT, funding code 01EO2107), sponsored by the German Federal Ministry of Education and Research (BMBF).

Conflict of interest

The authors declare no conflicts of interest that pertain to this work.

Please refer to the accompanying ICMJE disclosure forms for further details.

Authors' contributions

Study concept and design: KT, JS, EH, KHP. Data acquisition: KT, JS, EG, FEU, MJB, WG, RS, SK, MSM, MMM, TW, PAR, JR, FS, NB, NK, MP, PT, JF, SZ, CW, JT, AS, JC, TB, KHP. Analysis and interpretation of data: KT, JS, PL, MG, NK, EH, KHP. Drafting of manuscript, statistical analysis: JS. Critical revision of manuscript for important intellectual content: JS, KT, AS, JT, JC, TB, EH, KHP. Obtained funding: JS, SZ, CW, SC, JT, TB, EH, KHP. Administrative, technical, or material support: JT, JC, TB, EH, KHP. Study supervision: CW, JT, EH, KHP. All authors approved the final version of the article, including the authorship list.

Data availability

The datasets generated and/or analyzed during the study are not publicly available because of ongoing expansion and analysis, but are available from the corresponding author on reasonable request.

Acknowledgements

We thank all contributors of the ACLF-I as well as those involved in the recruitment of the validation cohorts in Aachen, Jena, and Bonn.

Supplementary data

Supplementary data to this article can be found online at https://doi.org/10.1016/j.jhepr.2025.101627.

References

Author names in bold designate shared co-first authorship

[1] Sepanlou SG, Safiri S, Bisignano C, et al., GBD 2017 Cirrhosis Collaborators. The global, regional, and national burden of cirrhosis by cause in 195 countries and territories, 1990-2017: a systematic analysis for the Global Burden of Disease Study 2017. Lancet Gastroenterol Hepatol 2020;5:245–266.

[2] Lee BP, Cullaro G, Vosooghi A, et al. Discordance in categorization of acute-on-chronic liver failure in the United Network for Organ Sharing database. J Hepatol 2022;76:1122–1126.

[3] **Zaccherini G**, **Weiss E**, Moreau R. Acute-on-chronic liver failure: definitions, pathophysiology and principles of treatment. JHEP Rep 2021;3:100176.

[4] European Association for the Study of the Liver. EASL Clinical Practice Guidelines on acute-on-chronic liver failure. J Hepatol 2023;79:461–491.

[5] Moreau R, Jalan R, Gines P, et al. Acute-on-chronic liver failure is a distinct syndrome that develops in patients with acute decompensation of cirrhosis. Gastroenterology 2013;144:1426–1437.

[6] Moreau R, Jalan R, Gines P, et al. Acute-on-chronic liver failure in cirrhosis. Nat Rev Dis Primers 2016;2:16041.

[7] Albillos A, Martin-Mateos R, Van der Merwe S, et al. Cirrhosis-associated immune dysfunction. Nat Rev Gastroenterol Hepatol 2022;19:112–134.

[8] Trebicka J, Fernandez J, Papp M, et al. The PREDICT study uncovers three clinical courses of acutely decompensated cirrhosis that have distinct pathophysiology. J Hepatol 2020;73:842–854.

[9] Trebicka J, Fernandez J, Arroyo V, et al. Reply to: correspondence on 'The PREDICT study uncovers three clinical courses of acutely decompensated cirrhosis that have distinct pathophysiology'. J Hepatol 2021;74:480–481.

[10] **Clària J**, **Stauber RE**, Coenraad MJ, et al. Systemic inflammation in decompensated cirrhosis: characterization and role in acute-on-chronic liver failure. Hepatology 2016;64:1249–1264.

[11] **Arroyo V**, **Angeli P**, **Moreau R**, et al. The systemic inflammation hypothesis: towards a new paradigm of acute decompensation and multiorgan failure in cirrhosis. J Hepatol 2021;74:670–685.

[12] Zanetto A, Pelizzaro F, Campello E, et al. Severity of systemic inflammation is the main predictor of ACLF and bleeding in individuals with acutely decompensated cirrhosis. J Hepatol 2023;78:301–311.

[13] **Jeong CY**, **Choi GH**, Jang ES, et al. Etiology and clinical characteristics of acute viral hepatitis in South Korea during 2020–2021: a prospective multicenter study. Scientific Rep 2023;13:14271.

[14] Patterson J, Hussey HS, Silal S, et al. Systematic review of the global epidemiology of viral-induced acute liver failure. BMJ Open 2020;10:e037473.

[15] **Kuri A**, **Jacobs BM**, Vickaryous N, et al. Epidemiology of Epstein-Barr virus infection and infectious mononucleosis in the United Kingdom. BMC Public Health 2020;20:912.
[16] Fowler K, Mucha J, Neumann M, et al. A systematic literature review of the global seroprevalence of cytomegalovirus: possible implications for treatment, screening, and vaccine development. BMC Public Health 2022;22:1659.
[17] Nakajima K, Hiejima E, Nihira H, et al. Case report: a case of Epstein-Barr virus-associated acute liver failure requiring hematopoietic cell transplantation after emergent liver transplantation. Front Immunol 2022;13:825806.
[18] Da Cunha T, Wu GY. Cytomegalovirus hepatitis in immunocompetent and immunocompromised hosts. J Clin Translational Hepatol 2021;9:106–115.
[19] Petrova M, Kamburov V. Epstein-Barr virus: silent companion or causative agent of chronic liver disease? World J Gastroenterol 2010;16:4130–4134.
[20] Yang Q, Zhou Z, Yang X, et al. Latent cytomegalovirus reactivation in patients with liver failure: a 10-year retrospective case-control study, 2011-2020. Front Cell Infect Microbiol 2021;11:642500.
[21] Rosi S, Poretto V, Cavallin M, et al. Hepatic decompensation in the absence of obvious precipitants: the potential role of cytomegalovirus infection/reactivation. BMJ Open Gastroenterol 2015;2:e000050.
[22] Picarda G, Benedict CA. Cytomegalovirus: shape-shifting the immune system. J Immunol 2018;200:3881–3889.
[23] Silva JM, Alves CEC, Pontes GS. Epstein-Barr virus: the mastermind of immune chaos. Front Immunol 2024;15:1297994.
[24] Razonable RR, Hayden RT. Clinical utility of viral load in management of cytomegalovirus infection after solid organ transplantation. Clin Microbiol Rev 2013;26:703–727.
[25] Blom KB, Kro GB, Midtvedt K, et al. Cellular immunity against cytomegalovirus and risk of infection after kidney transplantation. Front Immunol 2024;15:1414830.
[26] Li J, Gardiner BJ, Stankovic S, et al. Cytomegalovirus immunity assays predict viremia but not replication within the lung allograft. Transplant Direct 2023;9:e1501.
[27] Papazian L, Hraiech S, Lehingue S, et al. Cytomegalovirus reactivation in ICU patients. Intensive Care Med 2016;42:28–37.
[28] Guiouillier F, Derely J, Salvadori A, et al. Reactivation of Epstein-Barr virus among intensive care patients: a prospective observational study. Intensive Care Med 2024;50:418–426.
[29] Mattei A, Schiavoni L, Riva E, et al. Epstein-Barr virus, cytomegalovirus, and herpes simplex-1/2 reactivations in critically ill patients with COVID-19. Intensive Care Med Exp 2024;12:40.
[30] Frantzeskaki FG, Karampi ES, Kottaridi C, et al. Cytomegalovirus reactivation in a general, nonimmunosuppressed intensive care unit population: incidence, risk factors, associations with organ dysfunction, and inflammatory biomarkers. J Crit Care 2015;30:276–281.
[31] Forel JM, Martin-Loeches I, Luyt CE. Treating HSV and CMV reactivations in critically ill patients who are not immunocompromised: pro. Intensive Care Med 2014;40:1945–1949.
[32] Arroyo V, Angeli P, Moreau R, et al. Mechanisms of decompensation and organ failure in cirrhosis: from peripheral arterial vasodilation to systemic inflammation hypothesis. J Hepatol 2015;63:1272–1284.
[33] **Cao Z**, **Yao Y**, **Cai M**, et al. Blood markers for type-1, -2, and -3 inflammation are associated with severity of acutely decompensated cirrhosis. J Hepatol 2025;82:836–850.
[34] **Traska AK**, **Nowacki TM**, Vollenberg R, et al. Immunomonitoring via ELISPOT assay reveals attenuated T-cell immunity to CMV in immunocompromised liver-transplant patients. Cells 2024;13:741.
[35] Irvine KM, Ratnasekera I, Powell EE, et al. Causes and consequences of innate immune dysfunction in cirrhosis. Front Immunol 2019;10:293.
[36] **Weiss E**, **Rautou PE**, **Fasseu M**, et al. Type I interferon signaling in systemic immune cells from patients with alcoholic cirrhosis and its association with outcome. J Hepatol 2017;66:930–941.
[37] Chiche L, Forel JM, Thomas G, et al. Interferon-gamma production by natural killer cells and cytomegalovirus in critically ill patients. Crit Care Med 2012;40:3162–3169.
[38] van de Berg PJ, Heutinck KM, Raabe R, et al. Human cytomegalovirus induces systemic immune activation characterized by a type 1 cytokine signature. J Infect Dis 2010;202:690–699.
[39] Rooney M, Duduskar SN, Ghait M, et al. Type-I interferon shapes peritoneal immunity in cirrhosis and drives caspase-5-mediated progranulin release upon infection. J Hepatol 2024;81:971–982.
[40] **Hu J**, **Zhao H**, **Lou D**, et al. Human cytomegalovirus and Epstein-Barr virus infections, risk factors, and their influence on the liver function of patients with acute-on-chronic liver failure. BMC Infect Dis 2018;18:577.
[41] Gupta E, Ballani N, Kumar M, et al. Role of non-hepatotropic viruses in acute sporadic viral hepatitis and acute-on-chronic liver failure in adults. Indian J Gastroenterol 2015;34:448–452.
[42] Clària J, Arroyo V, Moreau R. The acute-on-chronic liver failure syndrome, or when the innate immune system goes astray. J Immunol 2016;197:3755–3761.
[43] Dancygier H. Viral infections by nonhepatotropic viruses. Clin Hepatol 2010;2:823–830.
[44] Cowley NJ, Owen A, Shiels SC, et al. Safety and efficacy of antiviral therapy for prevention of cytomegalovirus reactivation in immunocompetent critically ill patients: a randomized clinical trial. JAMA Intern Med 2017;177:774–783.
[45] Limaye AP, Stapleton RD, Peng L, et al. Effect of ganciclovir on IL-6 levels among cytomegalovirus-seropositive adults with critical illness a randomized clinical trial. JAMA 2017;318:731–740.
[46] Papazian L, Jaber S, Hraiech S, *et al.* Preemptive ganciclovir for mechanically ventilated patients with cytomegalovirus reactivation. Ann Intensive Care 202;11:33.
[47] Martin-Mateos R, Alvarez-Mon M, Albillos A. Dysfunctional immune response in acute-on-chronic liver failure: it takes two to tango. Front Immunol 2019;10:973.
[48] Rzepka M, Depka D, Gospodarek-Komkowska E, et al. Whole blood versus plasma samples-how does the type of specimen collected for testing affect the monitoring of cytomegalovirus viremia? Pathogens 2022;11:1384.
[49] Lisboa LF, Asberg A, Kumar D, et al. The clinical utility of whole blood versus plasma cytomegalovirus viral load assays for monitoring therapeutic response. Transplantation 2011;91:231–236.
[50] Kraft CS, Armstrong WS, Caliendo AM. Interpreting quantitative cytomegalovirus DNA testing: understanding the laboratory perspective. Clin Infect Dis 2012;54:1793–1797.

Keywords: Acute-on-chronic liver failure; Cytomegalovirus; Epstein–Barr virus; Human herpesvirus; Precipitant; Sustainer.
Received 20 March 2025; received in revised form 29 September 2025; accepted 2 October 2025; Available online 9 October 2025

Journal of Hepatology, Volume *8*

Supplemental information

Higher prevalence of cytomegalovirus and Epstein–Barr virus in acute-on-chronic liver failure

Keerthihan Thiyagarajah, Jannik Sonnenberg, Esra Görgülü, Pia Lembeck, Nico Kraus, Mirco Glitscher, Frank Erhard Uschner, Maximilian Joseph Brol, Wenyi Gu, Robert Schierwagen, Sabine Klein, Martin S. McCoy, Marcus Maximilian Mücke, Toska Wiedemann, Philipp A. Reuken, Johanna Reißing, Franziska Schneider, Nina Böhling, Michael Praktiknjo, Phil-Robin Tepasse, Julia Fischer, Stefan Zeuzem, Christoph Welsch, Sandra Ciesek, Andreas Stallmach, Jonel Trebicka, Johannes Chang, Tony Bruns, Eberhard Hildt, and Kai-Henrik Peiffer

Higher prevalence of Cytomegalovirus and Epstein–Barr virus in acute-on-chronic liver failure

Keerthihan Thiyagarajah, Jannik Sonnenberg (shared first)**,** Esra Görgülü, Pia Lembeck, Nico Kraus, Mirco Glitscher, Frank Erhard Uschner Maximilian Joseph Brol, Wenyi Gu, Robert Schierwagen, Sabine Klein, Martin S. McCoy, Markus Maximilian Mücke, Toska Wiedemann, Philipp A. Reuken, Johanna Reißing, Franziska Schneider, Nina Böhling, Michael Praktiknjo, I-Robin Tepasse, Julia Fischer, Stefan Zeuzem, Christoph Welsch, Sandra Ciesek, Andreas Stallmach, Jonel Trebicka, Johannes Chang, Tony Bruns, Eberhard Hildt, Kai-Henrik Peiffer

Table of contents

Supplementary analyses 2

Supplementary individual validation cohorts analyses 20

Supplementary sensitivity analyses 29

Supplementary methods 38

Supplementary references 45

Supplementary analyses

Table S1. Multiplex-qPCR master mix. Abbreviations: CN = Catalogue Number, ddH2O = double-distilled water

Ingredients	1x reaction
LightCycler Multiplex DNA Master (CN 07339577001)	4 µl
CMV probe (CN 08997837001)	0.5 µl
EBV probe (CN 10097710001)	0.5 µl
PhHV probe (CN 07093802001)	0.5 µl
ddH2O	2.5 µl

Table S2. Multiple regression analyses models and results of the ACLF-I and validation cohort. Linear regression models using the backwards method. Abbreviations: TIPS = Transjugular intrahepatic portosystemic stent, Regr. Coeff. = Regression coefficient, SE = Standard error, T = t-value

ACLF-I

CMV DNAemia

**Variables included**	**90-day mortality**, age, sex, aetiology of cirrhosis, Diabetes, Arterial hypertension, Coronary artery disease, Bacterial infections, Vasopressors, TIPS, beta blocker treatment, Supplemental oxygen including mechanical ventilation, West Haven grade of Hepatic encephalopathy, albumin, bilirubin, leucocytes, C-reactive protein, Gamma-glutamyltransferase, alkaline phosphatase				
	**Results:**	Regr. Coeff.	SE	T	*p*
	90-day mortality	0.39	0.10	3.81	0.00
	Etiology of cirrhosis	-0.02	0.01	-2.17	0.04
	Arterial hypertension	-0.38	0.13	-3.00	0.01
	Age	0.01	0.01	2.05	0.05
	C-reactive protein	0.04	0.02	2.08	0.05
	Gamma-glutamyltransferase	0.00	0.00	2.83	0.01
	Corrected $R^2 = 0.575$	F=8.652	*p*=0.000023		
**Variables included**	**28-day mortality**, age, sex, aetiology of cirrhosis, Diabetes, Arterial hypertension, Coronary artery disease, Bacterial infections, Vasopressors, TIPS, beta blocker treatment, Supplemental oxygen including mechanical ventilation, West Haven grade of Hepatic encephalopathy, albumin, bilirubin, leucocytes, C-reactive protein, Gamma-glutamyltransferase, alkaline phosphatase				
	**Results:**	Regr. Coeff.	SE	T	*p*
	28-day mortality	0.25	0.12	2.05	0.05
	Arterial hypertension	-0.30	0.14	-2.22	0.03
	C-reactive protein	0.04	0.02	1.99	0.06
	Gamma-glutamyltransferase	0.00	0.00	1.90	0.07
	Corrected $R^2 = 0.426$	F=7.321	*p*=0.00031		

EBV DNAemia

**Variables included**	**90-day mortality**, sex, aetiology of cirrhosis, Diabetes, Arterial hypertension, Ascites, Gastrointestinal bleeding, Bacterial infections, Vasopressors, Dialysis, TIPS, Immunosuppression, Antibiotic prophylaxis, Mechanical ventilation, albumin, leucocytes, C-reactive protein, Alanine transaminase				
	**Results:**	Regr. Coeff.	SE	T	*p*
	Sex	-0.09	0.04	-2.09	0.04
	90-day mortality	-0.14	0.05	-2.63	0.01
	Ascites	0.08	0.04	2.06	0.04
	TIPS	-0.11	0.05	-2.05	0.04
	Mechanical ventilation	0.28	0.12	2.38	0.02
	Leukocytes	0.02	0.00	3.93	0.00
	Corrected R^2 = 0.172	F=6.441	p=0.0000046		
**Variables included**	**28-day mortality**, sex, aetiology of cirrhosis, Diabetes, Arterial hypertension, Ascites, Gastrointestinal bleeding, Bacterial infections, Vasopressors, Dialysis, TIPS, Immunosuppression, Antibiotic prophylaxis, Mechanical ventilation, albumin, leucocytes, C-reactive protein, Alanine transaminase				
	**Results:**	Regr. Coeff.	SE	T	*p*
	28-day mortality	-0.14	0.07	-1.93	0.06
	Arterial hypertension	-0.08	0.04	-1.83	0.07
	Ascites	0.07	0.04	1.66	0.10
	Antibiotic prophylaxis	-0.10	0.05	-1.99	0.05
	Mechanical ventilation	0.28	0.12	2.35	0.02
	Leukocytes	0.02	0.00	3.78	0.00
	Corrected R^2 = 0.165	F=6.153	p=0.0000086		

CMV and/or EBV DNAemia

**Variables included**	**90-day mortality**, age, sex, aetiology of cirrhosis, Cerebral failure, Diabetes, Arterial hypertension, Coronary artery disease, Chronic renal failure, Ascites, Gastrointestinal bleeding, Bacterial infections, Vasopressors, TIPS, Immunosuppression, beta blocker treatment, Supplemental oxygen including mechanical ventilation, Albumin, bilirubin, Leukocytes, C-reactive protein, Aspartate aminotransferase, alkaline phosphatase, Gamma-glutamyltransferase				
	**Results:**	Regr. Coeff.	SE	T	*p*
	Arterial hypertension	-0.21	0.08	-2.62	0.01
	Gastrointestinal bleeding	-0.20	0.08	-2.54	0.01
	Vasopressors	0.60	0.21	2.83	0.01
	Bilirubin	0.01	0.00	1.90	0.06
	Leukocytes	0.01	0.01	1.71	0.09
	Aspartate aminotransferase	0.00	0.00	-1.84	0.07
	Corrected R^2 = 0.230	F=6.737	p=0.0000043		
**Variables included**	**28-day mortality**, age, sex, aetiology of cirrhosis, Cerebral failure, Diabetes, Arterial hypertension, Coronary artery disease, Chronic renal failure, Ascites, Gastrointestinal bleeding, Bacterial infections, Vasopressors, TIPS, Immunosuppression, beta blocker treatment, Supplemental oxygen including mechanical ventilation, Albumin, bilirubin, Leukocytes, C-reactive protein, Aspartate aminotransferase, alkaline phosphatase, Gamma-glutamyltransferase				
	**Results:**	Regr. Coeff.	SE	T	*p*

Arterial hypertension	-0.21	0.08	-2.62	0.01
Gastrointestinal bleeding	-0.20	0.08	-2.54	0.01
Vasopressors	0.60	0.21	2.83	0.01
Bilirubin	0.01	0.00	1.90	0.06
Leukocytes	0.01	0.01	1.71	0.09
Aspartate aminotransferase	0.00	0.00	-1.84	0.07
Corrected R^2 = 0.230	F=6.737	*p*=0.0000043		

Validation cohort

CMV DNAemia

Variables included: **90-day mortality**, sex, aetiology of cirrhosis, ACLF precipitant, cerebral failure, liver failure, age, albumin, sodium, creatinine, International normalized ratio, C-reactive protein, Aspartate aminotransferase, platelets

**Results:**	Regr. Coeff.	SE	T	*p*
90-day mortality	-0.27	0.12	-2.31	0.03
Sex	-0.28	0.11	-2.44	0.02
Cerebral failure	-0.85	0.26	-3.28	0.00
ACLF precipitant	0.09	0.04	2.19	0.03
Albumin	-0.01	0.01	-1.70	0.10
Sodium	-0.02	0.01	-1.71	0.09
Creatinine	0.17	0.04	4.65	0.00
Corrected R^2 = 0.459	F=7.677	*p*=0.0000033		

Variables included: **28-day mortality**, sex, aetiology of cirrhosis, ACLF precipitant, cerebral failure, liver failure, age, albumin, sodium, creatinine, International normalized ratio, C-reactive protein, Aspartate aminotransferase, platelets

**Results:**	Regr. Coeff.	SE	T	*p*
28-day mortality	-0.27	0.13	-2.13	0.04
Sex	-0.24	0.11	-2.10	0.04
Cerebral failure	-0.70	0.23	-3.00	0.00
Sodium	-0.02	0.01	-2.57	0.01
Creatinine	0.13	0.04	3.72	0.00
Corrected R^2 = 0.425	F=9.116	*p*=0.0000033		

EBV DNAemia

Variables included: **90-day mortality**, sex, aetiology of cirrhosis, ACLF precipitant, cerebral failure, age, creatinine, C-reactive protein, Aspartate aminotransferase, Alanine transaminase, platelets

**Results:**	Regr. Coeff.	SE	T	*p*
90-day mortality	-0.19	0.11	-1.74	0.09
Cerebral failure	-0.62	0.21	-2.96	0.01
Age	-0.01	0.01	-2.37	0.02
Creatinine	0.16	0.04	4.42	0.00
Aspartate aminotransferase	0.00	0.00	2.57	0.01

	Platelets	0.00	0.00	-1.76	0.09
	Corrected R^2 = 0.507	F=8.712	*p*=0.0000049		
**Variables included**	**28-day mortality**, sex, aetiology of cirrhosis, ACLF precipitant, cerebral failure, age, creatinine, C-reactive protein, Aspartate aminotransferase, Alanine transaminase, platelets				
	**Results:**	Regr. Coeff.	SE	T	*p*
	Cerebral failure	-0.63	0.22	-2.91	0.01
	Age	-0.02	0.01	-2.74	0.01
	Creatinine	0.19	0.04	5.13	0.00
	Aspartate aminotransferase	0.00	0.00	1.90	0.06
	Corrected R^2 = 0.460	F=10.577	*p*=0.0000056		

CMV and/or EBV DNAemia

**Variables included**	**90-day mortality**, age, sex, aetiology of cirrhosis, ACLF precipitant, cerebral failure, liver failure, Renal failure, International normalized ratio, platelets				
	**Results:**	Regr. Coeff.	SE	T	*p*
	90-day mortality	-0.24	0.11	-2.10	0.04
	Sex	-0.32	0.11	-2.81	0.01
	Cerebral failure	-0.97	0.25	-3.86	0.00
	ACLF precipitant	0.10	0.04	2.48	0.02
	Albumin	-0.02	0.01	-2.28	0.03
	Creatinine	0.18	0.04	4.82	0.00
	Corrected R^2 = 0.445	F=8.471	*p*=0.0000023		
**Variables included**	**28-day mortality**, age, sex, aetiology of cirrhosis, ACLF precipitant, Cerebral failure, Liver failure, Renal failure, International normalized ratio, platelets				
	**Results:**	Regr. Coeff.	SE	T	*p*
	28-day mortality	-0.28	0.13	-2.20	0.03
	Sex	-0.25	0.11	-2.22	0.03
	Cerebral failure	-0.72	0.23	-3.07	0.00
	Sodium mmol/l	-0.02	0.01	-2.47	0.02
	Creatinine mg/dl	0.13	0.04	3.66	0.00
	Corrected R^2 = 0.424	F=7.814	*p*=0.00015		

Table S3. ACLF-I analyses. Analyses separated by statistical method. Abbreviations: COPD = Chronic obstructive pulmonary disease, TIPS = Transjugular intrahepatic portosystemic stent, MELD = Model for End-Stage Liver Disease score, CLIF-C = Chronic Liver Failure Consortium, AD = Acute decompensation, OF = Organ failure; [1]calculation of Odds ratio not possible due to small patient groups; [2]calculation of Odds ratio due to table >2x2 not possible

Tests vs. CMV DNAemia

Pearson-Chi-Square test

**Variable**	***p***	**Odds ratio**	**Lower 95% CI**	**Higher 95% CI**
ACLF	0.00	3.51	1.47	8.34
Pre-ACLF	0.42	1.73	0.46	6.52
Sex	0.10	0.49	0.21	1.15

28-day mortality	0.10	2.46	0.81	7.47
90-day mortality	0.38	1.53	0.59	3.94
Liver failure	0.00	4.20	1.70	10.43
Renal failure	0.35	1.75	0.54	5.71
Cerebral failure	0.01	4.97	1.30	19.00
Coagulation failure	0.35	1.89	0.49	7.27
Circulation failure	0.48	1.53	0.47	4.92
Respiratory failure	0.80	1.32	0.15	11.77
Diabetes	0.29	0.59	0.23	1.56
COPD	0.95	0.93	0.11	7.89
Heart failure	0.33 [1]			
Hypertension	0.01	0.19	0.04	0.83
Coronary artery disease	0.11 [1]			
Chronic kidney disease	0.38 [1]			
Ascites	0.53	1.32	0.56	3.14
Hepatic encephalopathy	0.10	2.03	0.85	4.83
Gastrointestinal bleeding	0.39	0.61	0.20	1.88
Bacterial infections	0.30	1.55	0.68	3.55
Viral infections	0.63	1.71	0.18	15.93
Vasopressors	0.14	3.46	0.60	19.91
Dialysis	0.49 [1]			
TIPS	0.20	0.39	0.09	1.73
Immunosuppression	0.89	1.12	0.24	5.31
Beta blockers	0.01	0.28	0.10	0.80
Antibiotic prophylaxis	0.59	1.32	0.48	3.63
Oxygen supplementation	0.08	2.64	0.86	8.12
Mechanical ventilation	0.11	2.61	0.76	8.96
Any oxygen supplement	0.48	2.23	0.22	22.25
Aetiology of cirrhosis	0.21 [2]			
**Biserial rank-correlation**				
**Variable**	***p***	**Corr. Coeff.**	**DF**	
ACLF grade	0.65	0.08	38	
West Haven	0.07	0.24	196	
MELD	0.04	0.45	188	
MELD sodium	0.09	0.51	188	
CLIF-C ACLF	0.09	0.49	195	
CLIF-C AD	0.02	0.64	195	
CLIF-C OF	0.01	0.35	196	
Child-Pugh score	0.09	0.20	196	
Child-Pugh category	0.07	0.13	196	
**Shapiro-Wilk test**				
**Variables**	***p***	**W**		
Age	0.18	0.98		
Sodium	0.03	0.98		
Creatinine	0.00	0.73		
Bilirubin	0.00	0.73		
Albumin	0.21	0.99		
International normalized ratio	0.00	0.78		
Leukocytes	0.00	0.88		
C-reactive protein	0.00	0.77		
Aspartate aminotransferase	0.00	0.63		
Alanine aminotransferase	0.00	0.65		
Alkaline phosphatase	0.00	0.66		
Gamma-glutamyltransferase	0.00	0.27		
Platelets	0.00	0.84		
**T-test**				
**Variables**	***p***	**DF**	**T**	**Cohen's d**
Age	0.07	209	-1.432	-0.25
Albumin	0.21	207	-1.005	-0.18
**Mann-Whitney-U test**				
**Variables**	***p***	**U**	**Z**	**r**

Sodium	0.56	2055.00	-0.58	0.04
Creatinine	0.79	2139.00	-0.27	0.02
Bilirubin	0.03	1635.00	-2.14	0.15
International normalized ratio	0.95	2193.00	-0.06	0.00
Leukocytes	0.00	1440.00	-2.83	0.20
C-reactive protein	0.16	1578.00	-1.41	0.10
Aspartate aminotransferase	0.01	926.00	-2.44	0.20
Alanine aminotransferase	0.21	1741.00	-1.26	0.09
Alkaline phosphatase	0.01	869.00	-2.77	0.22
Gamma-glutamyltransferase	0.01	1484.00	-2.52	0.18
Platelets	0.43	1627.00	-0.66	0.05

Tests vs. EBV DNAemia

**Pearson-Chi-Square test**<br>**Variable**	***p***	**Odds ratio**	**Lower 95% CI**	**Higher 95% CI**
ACLF	0.03	3.01	1.10	8.62
Pre-ACLF	0.22	[1]		
Sex	0.02	0.32	0.12	0.88
28-day mortality	0.68	0.65	0.08	5.21
90-day mortality	0.44	0.55	0.12	2.54
Liver failure	0.59	1.44	0.38	5.42
Renal failure	0.75	1.28	0.27	6.12
Cerebral failure	0.63	1.71	0.19	15.09
Coagulation failure	0.92	0.90	0.11	7.46
Circulation failure	0.38	1.80	0.47	6.86
Respiratory failure	0.07	4.40	0.79	24.64
Diabetes	0.18	0.42	0.12	1.54
COPD	0.39	[1]		
Heart failure	0.63	1.71	0.19	15.09
Hypertension	0.10	0.30	0.07	1.37
Coronary artery disease	0.68	0.65	0.08	5.21
Chronic kidney disease	0.47	[1]		
Ascites	0.06	3.27	0.90	11.79
Hepatic encephalopathy	0.28	1.77	0.62	5.10
Gastrointestinal bleeding	0.29	0.45	0.10	2.04
Bacterial infections	0.09	2.44	0.86	6.89
Viral infections	0.53	[1]		
Vasopressors	0.00	8.89	1.81	43.75
Dialysis	0.26	3.48	0.34	35.42
TIPS	0.21	0.29	0.04	2.28
Immunosuppression	0.19	2.46	0.62	9.66
Beta blockers	0.31	0.58	0.20	1.67
Antibiotic prophylaxis	0.14	0.24	0.03	1.84
Oxygen supplementation	0.04	3.52	1.00	12.39
Mechanical ventilation	0.40	1.96	0.39	9.74
Any oxygen supplement	0.01	7.42	1.15	47.94
Aetiology of cirrhosis	0.93	[2]		
**Biserial rank-correlation**<br>**Variable**	***p***	**Corr. Coeff.**	**DF**	
ACLF grade	0.45	0.31	34	
West Haven	0.56	0.14	186	
MELD	0.33	0.35	182	
MELD sodium	0.22	0.38	182	
CLIF-C ACLF	0.10	0.43	185	
CLIF-C AD	0.38	0.66	185	
CLIF-C OF	0.43	0.23	186	
Child-Pugh score	0.49	0.34	186	
Child-Pugh category	0.14	0.09	186	
**Shapiro-Wilk test**<br>**Variables**	***p***	**W**		

Age	0.47	0.99
Sodium	0.04	0.97
Creatinine	0.00	0.79
Bilirubin	0.00	0.72
Albumin	0.27	0.99
International normalized ratio	0.00	0.77
Leukocytes	0.00	0.87
C-reactive protein	0.00	0.77
Aspartate aminotransferase	0.00	0.63
Alanine aminotransferase	0.00	0.63
Alkaline phosphatase	0.00	0.66
Gamma-glutamyltransferase	0.00	0.28
Platelets	0.00	0.91

**T-test**<br>**Variables**	***p***	**DF**	**T**	**Cohen's d**
Age	0.38	185	-0.30	-0.08
Albumin	0.09	26.02	-1.40	-0.24

**Mann-Whitney-U test**<br>**Variables**	***p***	**U**	**Z**	**r**
Sodium	0.36	1252.00	-0.91	0.07
Creatinine	0.72	1369.00	-0.36	0.03
Bilirubin	0.59	1330.50	-0.54	0.04
International normalized ratio	0.67	1354.50	-0.43	0.03
Leukocytes	0.03	980.00	-2.16	0.16
C-reactive protein	0.04	756.50	-2.01	0.15
Aspartate aminotransferase	0.82	719.50	-0.23	0.02
Alanine aminotransferase	0.03	818.00	-2.17	0.16
Alkaline phosphatase	0.87	960.00	-0.17	0.01
Gamma-glutamyltransferase	0.84	1198.50	-0.20	0.02
Platelets	0.53	916.50	-0.71	0.06

Tests vs. CMV and/or EBV DNAemia

**Pearson-Chi-Square test**<br>**Variable**	***p***	**Odds ratio**	**Lower 95% CI**	**Higher 95% CI**
ACLF	0.00	3.39	1.60	7.18
Pre-ACLF	0.94	0.95	0.26	3.51
Sex	0.01	0.42	0.21	0.84
28-day mortality	0.26	1.77	0.64	4.89
90-day mortality	0.99	1.01	0.43	2.38
Liver failure	0.01	2.78	1.24	6.25
Renal failure	0.59	1.34	0.46	3.89
Cerebral failure	0.03	3.80	1.10	13.12
Coagulation failure	0.46	1.56	0.47	5.18
Circulation failure	0.47	1.44	0.53	3.89
Respiratory failure	0.19	2.61	0.60	11.38
Diabetes	0.15	0.56	0.25	1.25
COPD	0.61	0.58	0.07	4.87
Heart failure	0.73	0.68	0.08	5.84
Hypertension	0.01	0.25	0.08	0.72
Coronary artery disease	0.17	0.26	0.03	2.01
Chronic kidney disease	0.27	[1]		
Ascites	0.16	1.69	0.81	3.54
Hepatic encephalopathy	0.16	1.69	0.81	3.52
Gastrointestinal bleeding	0.13	0.47	0.17	1.27
Bacterial infections	0.13	1.70	0.85	3.38
Viral infections	0.95	1.08	0.12	9.93
Vasopressors	0.03	4.49	1.07	18.77
Dialysis	0.78	1.39	0.14	13.73
TIPS	0.10	0.37	0.11	1.27
Immunosuppression	0.30	1.77	0.59	5.30

Beta blockers	0.03	0.42	0.20	0.92
Antibiotic prophylaxis	0.73	0.85	0.34	2.10
Oxygen supplementation	0.03	2.73	1.05	7.06
Mechanical ventilation	0.21	2.02	0.66	6.19
Any oxygen supplement	0.05	4.39	0.85	22.62
Aetiology of cirrhosis	0.39	[2]		
**Biserial rank-correlation**				
**Variable**	***p***	**Corr. Coeff.**	**DF**	
ACLF grade	0.75	0.11	43	
West Haven	0.12	0.19	211	
MELD	0.09	0.40	203	
MELD sodium	0.11	0.45	203	
CLIF-C ACLF	0.06	0.43	210	
CLIF-C AD	0.07	0.59	210	
CLIF-C OF	0.06	0.28	211	
Child-Pugh score	0.15	0.26	211	
Child-Pugh category	0.05	0.14	211	
**Shapiro-Wilk test**				
**Variables**	***p***	**W**		
Age	0.38	0.99		
Sodium	0.04	0.98		
Creatinine	0.00	0.74		
Bilirubin	0.00	0.73		
Albumin	0.15	0.99		
International normalized ratio	0.00	0.79		
Leukocytes	0.00	0.89		
C-reactive protein	0.00	0.79		
Aspartate aminotransferase	0.00	0.62		
Alanine aminotransferase	0.00	0.64		
Alkaline phosphatase	0.00	0.66		
Gamma-glutamyltransferase	0.00	0.27		
Platelets	0.00	0.85		
**T-test**				
**Variables**	***p***	**DF**	**T**	**Cohen's d**
Age	0.08	209	-1.43	-0.59
Albumin	0.16	207	-1.01	-0.52
**Mann-Whitney-U test**				
**Variables**	***p***	**U**	**Z**	**r**
Sodium	0.56	3283.00	-0.58	0.04
Creatinine	1.00	3484.00	0.00	0.00
Bilirubin	0.11	2932.00	-1.58	0.11
International normalized ratio	0.77	3382.00	-0.29	0.02
Leukocytes	0.00	2417.00	-3.00	0.21
C-reactive protein	0.07	2318.00	-1.83	0.13
Aspartate aminotransferase	0.07	1579.00	-1.84	0.15
Alanine aminotransferase	0.79	3049.00	-0.26	0.02
Alkaline phosphatase	0.06	1780.00	-1.86	0.14
Gamma-glutamyltransferase	0.05	2555.00	-2.00	0.14
Platelets	0.32	2585.00	-0.28	0.02

Table S4. Validation cohort analyses. Analyses separated by statistical method. Abbreviations: MELD = Model for End-Stage Liver Disease score, CLIF-C = Chronic Liver Failure Consortium, AD = Acute decompensation, OF = Organ failure, [1]calculation of Odds ratio not possible due to small patient groups; [2]calculation of significance not possible as one variable is a constant; [3]calculation of Odds ratio due to table >2x2 not possible

Tests vs. CMV DNAemia

**Pearson-Chi-Square test**				
**Variable**	***p***	**Odds ratio**	**Lower 95% CI**	**Higher 95% CI**
ACLF	0.00	40.63	12.04	137.10
pre-ACLF	0.07	3.47	0.87	13.89
Sex	0.15	0.53	0.22	1.27
28-day mortality	0.74	0.83	0.29	2.42
90-day mortality	0.86	0.93	0.39	2.20
Liver failure	0.01	10.59	1.07	105.34
Renal failure	0.00	24.56	9.35	64.49
Cerebral failure	0.17	[1]		
Coagulation failure	[2]			
Circulation failure	[2]			
Respiratory failure	[2]			
Immunosuppression	0.58	[3]		
Precipitant	0.34			
ACLF precipitant	0.04	[3]		
**Biserial rank-correlation**				
**Variable**	***p***	**Corr. Coeff.**	**DF**	
ACLF grade	0.62	0.25	38	
West Haven	0.34	0.15	149	
MELD	0.00	0.68	149	
CLIF-C ACLF	0.30	0.74	38	
CLIF-C AD	0.91	0.56	111	
CLIF-C OF	0.00	0.64	111	
Child-Pugh category	0.39	0.11	149	
**Shapiro-Wilk test**				
**Variables**	***p***	**W**		
Age	0.44	0.99		
Sodium	0.00	0.94		
Creatinine	0.00	0.74		
Bilirubin	0.00	0.39		
Albumin	0.27	0.99		
International normalized ratio	0.00	0.86		
Leukocytes	0.00	0.89		
C-reactive protein	0.00	0.74		
Aspartate aminotransferase	0.00	0.44		
Alanine aminotransferase	0.00	0.59		
Platelets	0.00	0.90		
**T-test**				
**Variables**	***p***	**DF**	**T**	**Cohen's d**
Age	0.43	147	-0.182	-0.035
Albumin	0.29	144	-0.565	-0.124
**Mann-Whitney-U test**				
**Variables**	***p***	**U**	**Z**	**r**
Sodium	0.18	1698	-1.33	0.11
Creatinine	0.00	655.5	-6.00	0.49
Bilirubin	0.32	1774	-0.99	0.08
International normalized ratio	0.02	1480.5	-2.33	0.19
Leukocytes	0.77	1928.5	-0.30	0.02
C-reactive protein	0.09	1612	-1.71	0.14
Aspartate aminotransferase	0.06	1243.5	-1.89	0.16
Alanine aminotransferase	0.50	1758.5	-0.67	0.06
Platelets	0.20	1707.5	-1.29	0.11

Tests vs. EBV DNAemia

**Pearson-Chi-Square test**				
**Variable**	***p***	**Odds ratio**	**Lower 95% CI**	**Higher 95% CI**

ACLF	0.00	46.88	9.50	231.32
pre-ACLF	0.10	4.17	0.66	26.30
Sex	0.02	0.20	0.04	0.90
28-day mortality	0.85	0.88	0.24	3.31
90-day mortality	0.80	1.15	0.41	3.25
Liver failure	0.67	[1]		
Renal failure	0.00	25.50	7.87	82.63
Cerebral failure	0.29	[1]		
Coagulation failure	[2]			
Circulation failure	[2]			
Respiratory failure	[2]			
Aetiology of cirrhosis	0.67	[3]		
ACLF precipitant	0.28	[3]		
Immunosuppression	0.20	[1]		

Biserial rank-correlation

Variable	*p*	Corr. Coeff.	DF
ACLF grade	0.11	0.32	27
West Haven	0.74	0.18	134
MELD	0.00	0.70	134
CLIF-C ACLF	0.01	0.92	27
CLIF-C AD	0.62	0.47	107
CLIF-C OF	0.00	0.63	96
Child-Pugh category	0.72	0.03	134

Shapiro-Wilk test

Variables	*p*	W
Age	0.72	0.99
Sodium	0.00	0.93
Creatinine	0.00	0.74
Bilirubin	0.00	0.50
Albumin	0.45	0.99
International normalized ratio	0.00	0.87
Leukocytes	0.00	0.89
C-reactive protein	0.00	0.72
Aspartate aminotransferase	0.00	0.44
Alanine aminotransferase	0.00	0.57
Platelets	0.00	0.89

T-test

Variables	*p*	DF	T	Cohen's d
Age	0.16	132	-0.994	-0.241
Albumin	0.49	129	0.026	0.007

Mann-Whitney-U test

Variables	*p*	U	Z	r
Sodium	0.40	1005	-0.84	0.07
Creatinine	0.00	321.5	-5.11	0.44
Bilirubin	0.79	1098	-0.26	0.02
International normalized ratio	0.52	1038.5	-0.64	0.06
Leukocytes	0.90	1119.5	-0.13	0.01
C-reactive protein	0.13	895	-1.53	0.13
Aspartate aminotransferase	0.02	558	-2.26	0.21
Alanine aminotransferase	0.30	956.5	-1.04	0.09
Platelets	0.19	929	-1.32	0.11

Tests vs. CMV and/or EBV DNAemia

Pearson-Chi-Square test

Variable	*p*	Odds ratio	Lower 95% CI	Higher 95% CI
ACLF	0.00	28.13	9.61	82.34
pre-ACLF	0.09	2.78	0.83	9.35
Sex	0.07	0.46	0.19	1.09
28-day mortality	0.85	0.91	0.34	2.47
90-day mortality	0.85	0.923	0.403	2.115

Liver failure	0.02	9.42	0.95	93.37
Renal failure	0.00	19.13	7.73	47.31
Cerebral failure	0.14 [1]			
Coagulation failure	[2]			
Circulation failure	[2]			
Respiratory failure	[2]			
Aetiology of cirrhosis	0.56 [3]			
ACLF precipitant	0.33 [3]			
Immunosuppression	0.05 [1]			
**Biserial rank-correlation**				
**Variable**	***p***	**Corr. Coeff.**	**DF**	
ACLF grade	0.59	0.25	39	
West Haven	0.22	0.17	153	
MELD	0.00	0.65	153	
CLIF-C ACLF	0.32	0.74	39	
CLIF-C AD	0.96	0.52	114	
CLIF-C OF	0.00	0.62	114	
Child-Pugh category	0.33	0.12	153	
**Shapiro-Wilk test**				
**Variables**	***p***	**W**		
Age	0.50	0.99		
Sodium	0.00	0.94		
Creatinine	0.00	0.74		
Bilirubin	0.00	0.39		
Albumin	0.30	0.99		
International normalized ratio	0.00	0.87		
Leukocytes	0.00	0.89		
C-reactive protein	0.00	0.74		
Aspartate aminotransferase	0.00	0.46		
Alanine aminotransferase	0.00	0.60		
Platelets	0.00	0.90		
**T-test**				
**Variables**	***p***	**DF**	**T**	**Cohen's d**
Age	0.43	147	-0.182	-0.03
Albumin	0.29	144	-0.565	-0.124
**Mann-Whitney-U test**				
**Variables**	***p***	**U**	**Z**	**r**
Sodium	0.18	1907	-1.33	0.11
Creatinine	0.00	835	-5.81	0.47
Bilirubin	0.42	2029.5	-0.81	0.07
International normalized ratio	0.04	1742.5	-2.03	0.16
Leukocytes	0.94	2206	-0.07	0.01
C-reactive protein	0.07	1783	-1.84	0.15
Aspartate aminotransferase	0.03	1278	-2.16	0.19
Alanine aminotransferase	0.30	1888.5	-1.04	0.08
Platelets	0.25	1948.5	-1.15	0.09

Table S5. ACLF-I cytokine analyses. Abbreviations: CCL = CC-chemokine ligand; CXCL = C-X-C motif chemokine ligand; GM-CSF = Granulocyte-macrophage colony-stimulating factor; IFN-gamma = Interferon gamma; IL = Interleukin; MIF = Macrophage migration inhibitory factor; TNF-a = Tumor necrosis factor a.

Non-ACLF no DNAemia vs. non-ACLF CMV and/or EBV DNAemia

**Shapiro-Wilk test**		
**Variables**	***p***	**W**
CCL2	0.00	0.83
CCL3	0.00	0.42
CCL4	0.00	0.20

CXCL10	0.00	0.33
GM-CSF	0.00	0.29
IFN-gamma	0.00	0.40
IL-10	0.00	0.35
IL-18	0.00	0.49
IL-1a	0.00	0.68
IL-1b	0.00	0.42
IL-1ra	0.00	0.84
IL-2	0.00	0.25
IL-6	0.00	0.43
MIF	0.00	0.65
TNF-a	0.00	0.89

Mann-Whitney-U test

**Variables**	***p***	**U**	**Z**	**r**
CCL2	0.84	323	-0.20	0.02
CCL3	0.71	323	-0.38	0.04
CCL4	0.60	308	-0.52	0.06
CXCL10	0.47	295	-0.72	0.08
GM-CSF	0.23	264	-1.21	0.14
IFN-gamma	0.75	321	-0.32	0.04
IL-10	0.95	337	-0.06	0.01
IL-18	0.85	329	-0.19	0.02
IL-1a	0.64	311	-0.46	0.05
IL-1b	0.31	280	-1.01	0.12
IL-1ra	0.85	329	-0.19	0.02
IL-2	0.71	320	-0.38	0.04
IL-6	0.67	314	-0.42	0.05
MIF	0.59	307	-0.53	0.06
TNF-a	0.52	299	-0.65	0.08

Non-ACLF no DNAemia vs. non-ACLF CMV DNAemia

Shapiro-Wilk test

**Variables**	***p***	**W**
CCL2	0.00	0.82
CCL3	0.00	0.44
CCL4	0.00	0.21
CXCL10	0.00	0.32
GM-CSF	0.00	0.29
IFN-gamma	0.00	0.40
IL-10	0.00	0.34
IL-18	0.00	0.50
IL-1a	0.00	0.69
IL-1b	0.00	0.43
IL-1ra	0.00	0.86
IL-2	0.00	0.26
IL-6	0.00	0.44
MIF	0.00	0.66
TNF-a	0.00	0.91

Mann-Whitney-U test

**Variables**	***p***	**U**	**Z**	**r**
CCL2	0.43	147	-0.79	0.10
CCL3	0.55	165	-0.60	0.07
CCL4	0.43	150	-0.78	0.09
CXCL10	0.31	140	-1.01	0.12
GM-CSF	0.73	171	-0.34	0.04
IFN-gamma	0.36	145	-0.91	0.11
IL-10	0.84	177	-0.21	0.02
IL-18	0.47	153	-0.72	0.09
IL-1a	0.85	177	-0.19	0.02
IL-1b	0.24	135	-1.19	0.14

IL-1ra	0.40	147	-0.84	0.10
IL-2	0.60	165	-0.53	0.06
IL-6	0.56	159	-0.58	0.07
MIF	0.99	186	-0.01	0.00
TNF-a	0.73	170	-0.35	0.04

Non-ACLF no DNAemia vs. non-ACLF EBV DNAemia

**Shapiro-Wilk test**		
**Variables**	***p***	**W**
CCL2	0.00	0.84
CCL3	0.00	0.47
CCL4	0.00	0.20
CXCL10	0.00	0.32
GM-CSF	0.00	0.27
IFN-gamma	0.00	0.41
IL-10	0.00	0.35
IL-18	0.00	0.48
IL-1a	0.00	0.66
IL-1b	0.00	0.62
IL-1ra	0.00	0.83
IL-2	0.00	0.22
IL-6	0.00	0.43
MIF	0.00	0.66
TNF-a	0.00	0.89

**Mann-Whitney-U test**				
**Variables**	***p***	**U**	**Z**	**r**
CCL2	0.57	129	-0.57	0.07
CCL3	0.20	115	-1.28	0.16
CCL4	0.95	153	-0.06	0.01
CXCL10	1.00	155	0.00	0.00
GM-CSF	0.13	93	-1.50	0.18
IFN-gamma	0.61	134	-0.51	0.06
IL-10	0.90	150	-0.13	0.02
IL-18	0.62	134	-0.50	0.06
IL-1a	0.35	116	-0.93	0.11
IL-1b	0.80	145	-0.26	0.03
IL-1ra	0.22	104	-1.22	0.15
IL-2	1.00	155	0.00	0.00
IL-6	0.19	101	-1.30	0.16
MIF	0.42	121	-0.81	0.10
TNF-a	0.17	97	-1.38	0.17

Non-ACLF no DNAemia vs. pre-ACLF no DNAemia

**Shapiro-Wilk test**		
**Variables**	***p***	**W**
CCL2	0.00	0.81
CCL3	0.00	0.39
CCL4	0.00	0.23
CXCL10	0.00	0.36
GM-CSF	0.00	0.32
IFN-gamma	0.00	0.45
IL-10	0.00	0.36
IL-18	0.00	0.50
IL-1a	0.00	0.71
IL-1b	0.00	0.49
IL-1ra	0.00	0.83
IL-2	0.00	0.25
IL-6	0.00	0.18
MIF	0.00	0.67
TNF-a	0.00	0.93

Mann-Whitney-U test				
**Variables**	***p***	**U**	**Z**	**r**
CCL2	0.95	271	-0.06	0.01
CCL3	0.10	202	-1.65	0.20
CCL4	0.05	167	-1.95	0.23
CXCL10	0.47	238	-0.72	0.09
GM-CSF	0.02	145	-2.35	0.28
IFN-gamma	0.02	143	-2.37	0.28
IL-10	0.02	141	-2.39	0.28
IL-18	0.82	266	-0.23	0.03
IL-1a	0.12	189	-1.57	0.19
IL-1b	0.04	167	-2.05	0.24
IL-1ra	0.41	231	-0.83	0.10
IL-2	0.02	160	-2.29	0.27
IL-6	0.01	136	-2.47	0.29
MIF	0.77	262	-0.29	0.03
TNF-a	0.03	153	-2.18	0.26

Non-ACLF no DNAemia vs. pre-ACLF CMV DNAemia

Shapiro-Wilk test		
**Variables**	***p***	**W**
CCL2	0.00	0.83
CCL3	0.00	0.45
CCL4	0.00	0.21
CXCL10	0.00	0.33
GM-CSF	0.00	0.30
IFN-gamma	0.00	0.37
IL-10	0.00	0.28
IL-18	0.00	0.45
IL-1a	0.00	0.64
IL-1b	0.00	0.63
IL-1ra	0.00	0.82
IL-2	0.00	0.28
IL-6	0.00	0.46
MIF	0.00	0.70
TNF-a	0.00	0.88

Mann-Whitney-U test				
**Variables**	***p***	**U**	**Z**	**r**
CCL2	0.95	90	-0.06	0.01
CCL3	0.00	11	-3.19	0.40
CCL4	0.48	71	-0.71	0.09
CXCL10	0.23	55	-1.19	0.15
GM-CSF	0.07	35	-1.84	0.23
IFN-gamma	0.34	63	-0.95	0.12
IL-10	0.15	48	-1.42	0.18
IL-18	0.49	71	-0.69	0.09
IL-1a	0.08	38	-1.72	0.21
IL-1b	0.87	88	-0.17	0.02
IL-1ra	0.05	32	-1.92	0.24
IL-2	0.15	53	-1.44	0.18
IL-6	0.24	56	-1.17	0.15
MIF	0.26	57	-1.13	0.14
TNF-a	0.12	43	-1.56	0.19

Non-ACLF no DNAemia vs. ACLF no DNAemia

Shapiro-Wilk test		
**Variables**	***p***	**W**
CCL2	0.00	0.86
CCL3	0.00	0.55

CCL4	0.00	0.24
CXCL10	0.00	0.40
GM-CSF	0.00	0.34
IFN-gamma	0.00	0.40
IL-10	0.00	0.42
IL-18	0.00	0.52
IL-1a	0.00	0.71
IL-1b	0.00	0.65
IL-1ra	0.00	0.79
IL-2	0.00	0.23
IL-6	0.00	0.57
MIF	0.00	0.71
TNF-a	0.00	0.91

**Mann-Whitney-U test**				
**Variables**	***p***	**U**	**Z**	**r**
CCL2	0.99	335	-0.01	0.00
CCL3	0.04	232	-2.05	0.24
CCL4	0.08	227	-1.76	0.21
CXCL10	0.06	220	-1.87	0.22
GM-CSF	0.01	164	-2.76	0.32
IFN-gamma	0.18	255	-1.34	0.16
IL-10	0.01	177	-2.54	0.30
IL-18	0.01	174	-2.58	0.30
IL-1a	0.07	225	-1.79	0.21
IL-1b	0.31	279	-1.02	0.12
IL-1ra	0.04	206	-2.08	0.24
IL-2	0.02	203	-2.36	0.28
IL-6	0.00	151	-2.94	0.34
MIF	0.28	271	-1.09	0.13
TNF-a	0.88	331	-0.15	0.02

Non-ACLF no DNAemia vs. ACLF CMV and/or EBV DNAemia

**Shapiro-Wilk test**		
**Variables**	***p***	**W**
CCL2	0.00	0.83
CCL3	0.00	0.52
CCL4	0.00	0.21
CXCL10	0.00	0.40
GM-CSF	0.00	0.34
IFN-gamma	0.00	0.41
IL-10	0.00	0.41
IL-18	0.00	0.53
IL-1a	0.00	0.70
IL-1b	0.00	0.63
IL-1ra	0.00	0.75
IL-2	0.00	0.24
IL-6	0.00	0.46
MIF	0.00	0.70
TNF-a	0.00	0.92

**Mann-Whitney-U test**				
**Variables**	***p***	**U**	**Z**	**r**
CCL2	0.22	102	-1.22	0.15
CCL3	0.03	103	-2.23	0.27
CCL4	0.12	115	-1.55	0.19
CXCL10	0.00	48	-2.98	0.36
GM-CSF	0.00	47	-3.04	0.37
IFN-gamma	0.17	123	-1.38	0.17
IL-10	0.01	58	-2.78	0.34
IL-18	0.14	119	-1.46	0.18
IL-1a	0.01	71	-2.49	0.30

IL-1b	0.37	148	-0.89	0.11
IL-1ra	0.09	107	-1.72	0.21
IL-2	0.01	79	-2.59	0.31
IL-6	0.12	89	-1.58	0.19
MIF	0.40	147	-0.84	0.10
TNF-a	0.03	84	-2.22	0.27

Non-ACLF no DNAemia vs. ACLF CMV DNAemia

**Shapiro-Wilk test**		
**Variables**	***p***	**W**
CCL2	0.00	0.83
CCL3	0.00	0.50
CCL4	0.00	0.21
CXCL10	0.00	0.32
GM-CSF	0.00	0.27
IFN-gamma	0.00	0.41
IL-10	0.00	0.35
IL-18	0.00	0.49
IL-1a	0.00	0.68
IL-1b	0.00	0.61
IL-1ra	0.00	0.86
IL-2	0.00	0.23
IL-6	0.00	0.46
MIF	0.00	0.68
TNF-a	0.00	0.92

**Mann-Whitney-U test**				
**Variables**	***p***	**U**	**Z**	**r**
CCL2	0.61	48	-0.51	0.06
CCL3	0.10	51	-1.67	0.21
CCL4	0.68	80	-0.41	0.05
CXCL10	0.03	23	-2.19	0.27
GM-CSF	0.02	17	-2.41	0.30
IFN-gamma	0.41	67	-0.82	0.10
IL-10	0.02	17	-2.39	0.30
IL-18	0.77	84	-0.30	0.04
IL-1a	0.08	37	-1.75	0.22
IL-1b	0.47	72	-0.72	0.09
IL-1ra	0.48	71	-0.70	0.09
IL-2	0.16	54	-1.40	0.17
IL-6	0.12	22	-1.54	0.19
MIF	0.85	87	-0.19	0.02
TNF-a	0.17	50	-1.36	0.17

Non-ACLF no DNAemia vs. ACLF EBV DNAemia

**Shapiro-Wilk test**		
**Variables**	***p***	**W**
CCL2	0.00	0.84
CCL3	0.00	0.51
CCL4	0.00	0.21
CXCL10	0.00	0.36
GM-CSF	0.00	0.31
IFN-gamma	0.00	0.41
IL-10	0.00	0.38
IL-18	0.00	0.51
IL-1a	0.00	0.68
IL-1b	0.00	0.61
IL-1ra	0.00	0.86
IL-2	0.00	0.23
IL-6	0.00	0.45

MIF	0.00	0.67		
TNF-a	0.00	0.92		
**Mann-Whitney-U test**				
**Variables**	***p***	**U**	**Z**	**r**
CCL2	0.12	3	-1.54	0.20
CCL3	0.18	12	-1.34	0.17
CCL4	0.23	10	-1.19	0.15
CXCL10	0.11	2	-1.59	0.20
GM-CSF	0.11	2	-1.62	0.20
IFN-gamma	0.74	25	-0.33	0.04
IL-10	0.10	1	-1.65	0.21
IL-18	0.17	6	-1.37	0.17
IL-1a	0.31	13	-1.02	0.13
IL-1b	0.34	15	-0.95	0.12
IL-1ra	0.41	16	-0.82	0.10
IL-2	0.10	5	-1.66	0.21
IL-6	0.78	26	-0.28	0.03
MIF	0.38	15	-0.88	0.11
TNF-a	0.60	22	-0.52	0.07

Non-ACLF no DNAemia vs. ACLF CMV and/or EBV DNAemia

**Shapiro-Wilk test**				
**Variables**	***p***	**W**		
CCL2	0.00	0.83		
CCL3	0.00	0.51		
CCL4	0.00	0.21		
CXCL10	0.00	0.36		
GM-CSF	0.00	0.30		
IFN-gamma	0.00	0.42		
IL-10	0.00	0.38		
IL-18	0.00	0.53		
IL-1a	0.00	0.69		
IL-1b	0.00	0.64		
IL-1ra	0.00	0.75		
IL-2	0.00	0.24		
IL-6	0.00	0.45		
MIF	0.00	0.69		
TNF-a	0.00	0.92		
**Mann-Whitney-U test**				
**Variables**	***p***	**U**	**Z**	**r**
CCL2	0.16	25	-1.41	0.18
CCL3	0.27	40	-1.09	0.14
CCL4	0.15	25	-1.43	0.18
CXCL10	0.13	23	-1.50	0.19
GM-CSF	0.18	28	-1.33	0.17
IFN-gamma	0.23	31	-1.21	0.15
IL-10	0.40	40	-0.85	0.11
IL-18	0.04	10	-2.01	0.25
IL-1a	0.12	22	-1.57	0.20
IL-1b	0.17	29	-1.36	0.17
IL-1ra	0.11	20	-1.62	0.20
IL-2	0.07	21	-1.81	0.23
IL-6	0.42	41	-0.81	0.10
MIF	0.06	13	-1.89	0.24
TNF-a	0.06	13	-1.91	0.24

Table S6. Serological analysis. Abbreviations: CMV = Cytomegalovirus, EBV = Epstein-Barr-virus, VCA = Capsid-Antigen, EBNA = Epstein-Barr nuclear antigen, IgG = Immunglobulin G, IgM = Immunglobulin M; [1]calculation of Odds ratio not possible

due to small patient groups; [2]calculation of significance not possible as one variable is a constant

ACLF-I cohort tests vs. DNAemia

Pearson-Chi-Square test<br>Variable	*p*	Odds ratio	Lower 95% CI	Higher 95% CI
CMV IgG	0.82	1.11	0.45	2.77
CMV IgM	0.24	2.71	0.49	14.90
EBNA-1 IgG	1.00	1.00	0.13	7.47
EBV-VCA IgM	0.05	6.88	0.79	60.06

ACLF-I cohort tests vs. CMV DNAemia

Pearson-Chi-Square test<br>Variable	*p*	Odds ratio	Lower 95% CI	Higher 95% CI
CMV IgG	0.65	1.28	0.44	3.67
CMV IgM	0.14	3.62	0.61	21.44

ACLF-I cohort tests vs. EBV DNAemia

Pearson-Chi-Square test<br>Variable	*p*	Odds ratio	Lower 95% CI	Higher 95% CI
EBNA-1 IgG	0.89	0.84	0.07	9.96
EBV-VCA IgM	0.15	5.20	0.44	61.68

ACLF-I cohort tests in the respective subgroups vs. ACLF

Pearson-Chi-Square test<br>Variable	*p*	Odds ratio	Lower 95% CI	Higher 95% CI
CMV IgG	0.04	3.27	1.03	10.45
CMV IgM	0.54	1.68	0.31	9.07
EBNA-1 IgG	0.16	[1]		
EBV-VCA IgM	0.85	[1]		

Validation cohort tests vs. DNAemia

Pearson-Chi-Square test<br>Variable	*p*	Odds ratio	Lower 95% CI	Higher 95% CI
CMV IgG	0.92	1.111	0.148	8.367
CMV IgM	0.12	[1]		
EBNA-1 IgG	0.02	[1]		
EBV-VCA IgM	[2]			

Validation cohort tests vs. CMV DNAemia

Pearson-Chi-Square test<br>Variable	*p*	Odds ratio	Lower 95% CI	Higher 95% CI
CMV IgG	0.74	0.71	0.10	5.12
CMV IgM	0.25	[1]		

Validation cohort tests vs. EBV DNAemia

Pearson-Chi-Square test<br>Variable	*p*	Odds ratio	Lower 95% CI	Higher 95% CI
EBNA-1 IgG	0.10	[1]		
EBV-VCA IgM	[2]			

Supplementary individual validation cohorts analyses

Table S7. Validation cohort from Aachen/Jena. Analyses separated by statistical method, linear regression models using the backwards method. Abbreviations: MELD = Model for End-Stage Liver Disease score, CLIF-C = Chronic Liver Failure Consortium, AD = Acute decompensation, OF = Organ failure, [1]calculation of Odds ratio not possible due to small patient groups; [2]calculation of significance not possible as one variable is a constant; [3]calculation of Odds ratio due to table >2x2 not possible

CMV DNAemia

**Variables included**	**90-day mortality**, age, sex, aetiology of cirrhosis, ACLF precipitant, West Haven grade of Hepatic encephalopathy, bilirubin, albumin, sodium, creatinine, International normalized ratio, platelets				
	**Results:**	Regr. Coeff.	SE	T	*p*
	90-day mortality	-0.28	0.15	-1.82	0.08
	West Haven grade of Hepatic encephalopathy	-0.18	0.08	-2.14	0.04
	International normalized ratio	0.35	0.20	1.75	0.09
	Corrected R² = 0.225	F=4.484	p=0.0095		
**Variables included**	**28-day mortality**, age, sex, aetiology of cirrhosis, ACLF precipitant, West Haven grade of Hepatic encephalopathy, bilirubin, albumin, sodium, creatinine, International normalized ratio, platelets				
	**Results:**	Regr. Coeff.	SE	T	*p*
	28-day mortality	-0.39	0.18	-2.17	0.04
	Bilirubin	0.02	0.01	2.38	0.02
	West Haven grade of Hepatic encephalopathy	-0.24	0.08	-3.01	0.01
	Sodium	-0.02	0.01	-1.72	0.10
	Creatinine	0.09	0.05	1.88	0.07
	Corrected R² = 0.331	F=4.559	p=0.003		

EBV DNAemia

**Variables included**	**90-day mortality**, age, sex, aetiology of cirrhosis, ACLF precipitant, West Haven grade of Hepatic encephalopathy, albumin, creatinine, platelets				
	**Results:**	Regr. Coeff.	SE	T	*p*
	West Haven grade of Hepatic encephalopathy	-0.17	0.08	-2.13	0.04
	Age	-0.02	0.01	-2.26	0.03
	Creatinine	0.13	0.06	2.13	0.04
	Corrected R² = 0.371	F=5.907	p=0.004		
**Variables included**	**28-day mortality**, age, sex, aetiology of cirrhosis, ACLF precipitant, West Haven grade of Hepatic encephalopathy, albumin, creatinine, platelets				
	**Results:**	Regr. Coeff.	SE	T	*p*
	West Haven grade of Hepatic encephalopathy	-0.17	0.08	-2.13	0.04
	Age	-0.02	0.01	-2.26	0.03
	Creatinine	0.13	0.06	2.13	0.04
	Corrected R² = 0.371	F=5.907	p=0.004		

CMV and/or EBV DNAemia

**Variables included**	**90-day mortality**, age, sex, aetiology of cirrhosis, ACLF precipitant, Cerebral failure, Liver failure, Renal failure, International normalized ratio, platelets				
	**Results:**	Regr. Coeff.	SE	T	*p*
	90-day mortality	-0.28	0.15	-1.88	0.07
	West Haven grade of Hepatic encephalopathy	-0.18	0.08	-2.20	0.03
	International normalized ratio	0.34	0.20	1.76	0.09
	Corrected R^2 = 0.362	F=6.245	p=0.00074		
**Variables included**	**28-day mortality**, age, sex, aetiology of cirrhosis, ACLF precipitant, Cerebral failure, Liver failure, Renal failure, International normalized ratio, platelets				
	**Results:**	Regr. Coeff.	SE	T	*p*
	**28-day mortality**	-0.55	0.16	-3.43	0.00
	Cerebral failure	-0.98	0.22	-4.42	0.00
	International normalized ratio	0.36	0.16	2.28	0.03
	Platelets	0.00	0.00	-2.08	0.05
	Corrected R^2 = 0.424	F=7.814	p=0.00015		

Tests vs. CMV DNAemia

**Pearson-Chi-Square test**				
**Variable**	***p***	**Odds ratio**	**Lower 95% CI**	**Higher 95% CI**
ACLF	0.00	89.82	19.82	738.64
pre-ACLF	0.02	[1]		
Sex	0.43	0.65	0.22	1.93
28-day mortality	0.27	0.41	0.08	2.09
90-day mortality	0.26	0.54	0.18	1.59
Liver failure	0.09	6.00	0.59	60.79
Renal failure	0.00	89.82	19.82	738.64
Cerebral failure	0.06	[1]		
Coagulation failure	[2]			
Circulation failure	[2]			
Respiratory failure	[2]			
Aetiology of cirrhosis	0.23	[3]		
ACLF precipitant	0.19	[3]		
Immunosuppression	0.46	[1]		
**Biserial rank-correlation**				
**Variable**	***p***	**Corr. Coeff.**	**DF**	
ACLF grade	0.62	0.25	38	
West Haven	0.02	0.34	76	
MELD	0.00	0.81	76	
CLIF-C ACLF	0.11	0.74	38	
CLIF-C AD	0.82	1.00	38	
CLIF-C OF	0.31	0.41	38	
Child-Pugh category	0.64	-0.06	76	
**Shapiro-Wilk test**				
**Variables**	***p***	**W**		
Age	0.55	0.98		
Sodium	0.00	0.92		
Creatinine	0.00	0.85		
Bilirubin	0.00	0.49		
Albumin	0.58	0.98		
International normalized ratio	0.00	0.91		
Leukocytes	0.00	0.92		

C-reactive protein	0.00	0.83
Aspartate aminotransferase	0.00	0.48
Alanine aminotransferase	0.00	0.63
Platelets	0.00	0.87

T-test

Variables	*p*	DF	T	Cohen’s d
Age	0.41	74.00	-0.23	-0.06
Albumin	0.11	35.69	1.24	0.35

Mann-Whitney-U test

Variables	*p*	U	Z	r
Sodium	0.25	556.50	-1.14	0.13
Creatinine	0.00	108.00	-6.01	0.69
Bilirubin	0.82	641.00	-0.22	0.03
International normalized ratio	0.08	499.50	-1.77	0.20
Leukocytes	0.49	597.50	-0.69	0.08
C-reactive protein	0.98	659.00	-0.03	0.00
Aspartate aminotransferase	0.84	418.50	-0.20	0.03
Alanine aminotransferase	0.55	593.50	-0.60	0.07
Platelets	0.09	505.50	-1.69	0.19

Tests vs. EBV DNAemia

Pearson-Chi-Square test

Variable	*p*	Odds ratio	Lower 95% CI	Higher 95% CI
ACLF	0.00	51.82	6.14	437.17
pre-ACLF	0.02	[1]		
Sex	0.15	0.32	0.07	1.61
28-day mortality	0.71	0.73	0.14	3.87
90-day mortality	0.80	0.86	0.26	2.87
Liver failure	0.57	[1]		
Renal failure	0.00	51.82	6.14	437.17
Cerebral failure	0.14	[1]		
Coagulation failure	[2]			
Circulation failure	[2]			
Respiratory failure	[2]			
Aetiology of cirrhosis	0.23	[3]		
ACLF precipitant	0.44	[3]		
Immunosuppression	0.57	[1]		

Biserial rank-correlation

Variable	*p*	Corr. Coeff.	DF
ACLF grade	0.11	0.32	27
West Haven	0.02	0.40	65
MELD	0.00	0.84	65
CLIF-C ACLF	0.01	0.92	27
CLIF-C AD	0.50	0.56	38
CLIF-C OF	0.18	0.42	27
Child-Pugh category	0.23	-0.15	65

Shapiro-Wilk test

Variables	*p*	W
Age	0.96	0.99
Sodium	0.00	0.89
Creatinine	0.00	0.83
Bilirubin	0.00	0.57
Albumin	0.27	0.97
International normalized ratio	0.00	0.90
Leukocytes	0.00	0.91
C-reactive protein	0.00	0.81
Aspartate aminotransferase	0.00	0.51
Alanine aminotransferase	0.00	0.62
Platelets	0.00	0.87

T-test

Variables	*p*	DF	T	Cohen's d
Age	0.10	63.00	-1.28	-0.37
Albumin	0.08	17.62	1.46	0.54

Mann-Whitney-U test

Variables	*p*	U	Z	r
Sodium	0.69	365.50	-0.41	0.05
Creatinine	0.00	68.50	-4.93	0.61
Bilirubin	0.99	391.00	-0.02	0.00
International normalized ratio	0.32	327.50	-0.99	0.12
Leukocytes	0.31	325.50	-1.01	0.13
C-reactive protein	0.85	380.00	-0.18	0.02
Aspartate aminotransferase	0.68	250.00	-0.41	0.06
Alanine aminotransferase	0.66	355.50	-0.44	0.06
Platelets	0.02	235.00	-2.39	0.30

Tests vs. CMV and/or EBV DNAemia

Pearson-Chi-Square test

Variable	*p*	Odds ratio	Lower 95% CI	Higher 95% CI
ACLF	0.00	46.64	9.56	227.63
pre-ACLF	0.00	[1]		
Sex	0.34	0.59	0.20	1.75
28-day mortality	0.46	0.59	0.14	2.43
90-day mortality	0.33	0.60	0.21	1.69
Liver failure	0.11	5.54	0.55	55.96
Renal failure	0.00	46.64	9.56	227.63
Cerebral failure	0.05	[1]		
Coagulation failure	[2]			
Circulation failure	[2]			
Respiratory failure	[2]			
Aetiology of cirrhosis	0.23	[3]		
ACLF precipitant	0.23	[3]		
Immunosuppression	0.44	[1]		

Biserial rank-correlation

Variable	*p*	Corr. Coeff.	DF
ACLF grade	0.59	0.25	39
West Haven	0.03	0.35	78
MELD	0.00	0.80	78
CLIF-C ACLF	0.08	0.74	39
CLIF-C AD	0.75	0.81	39
CLIF-C OF	0.25	0.42	39
Child-Pugh category	0.82	-0.03	78

Shapiro-Wilk test

Variables	*p*	W
Age	0.67	0.99
Sodium	0.00	0.92
Creatinine	0.00	0.84
Bilirubin	0.00	0.48
Albumin	0.54	0.98
International normalized ratio	0.00	0.91
Leukocytes	0.00	0.92
C-reactive protein	0.00	0.83
Aspartate aminotransferase	0.00	0.50
Alanine aminotransferase	0.00	0.64
Platelets	0.00	0.87

T-test

Variables	*p*	DF	T	Cohen's d
Age	0.37	76.00	-0.33	-0.08
Albumin	0.11	40.28	1.26	0.34

Mann-Whitney-U test

Variables	*p*	U	Z	r

Sodium	0.46	639.00	-0.74	0.08
Creatinine	0.00	147.50	-5.82	0.66
Bilirubin	0.66	668.00	-0.44	0.05
International normalized ratio	0.06	532.50	-1.85	0.21
Leukocytes	0.39	627.00	-0.86	0.10
C-reactive protein	0.96	706.00	-0.05	0.01
Aspartate aminotransferase	0.68	439.50	-0.41	0.05
Alanine aminotransferase	0.73	663.50	-0.34	0.04
Platelets	0.04	514.50	-2.03	0.23

Table S7. Validation cohort from Bonn. Analyses separated by statistical method, linear regression models using the backwards method. Abbreviations: MELD = Model for End-Stage Liver Disease score, CLIF-C = Chronic Liver Failure Consortium, AD = Acute decompensation, OF = Organ failure, [1]calculation of Odds ratio not possible due to small patient groups; [2]calculation of significance not possible as one variable is a constant; [3]calculation of Odds ratio due to table >2x2 not possible

CMV DNAemia

**Variables included**	**90-day mortality**, age, sex, aetiology of cirrhosis, ACLF precipitant, Sodium, Creatinine, Bilirubin, Albumin, C-reactive protein, Alkaline phosphatase, Haemoglobin, Time from ACLF to Death				
	**Results:**	Regr. Coeff.	SE	T	*p*
	Sex	0.67	0.33	2.00	0.09
	90-day mortality	-0.69	0.31	-2.26	0.06
	Aetiology of cirrhosis	0.24	0.07	3.58	0.01
	Age	0.03	0.01	3.25	0.02
	Sodium	-0.06	0.02	-3.22	0.02
	Bilirubin	-0.48	0.15	-3.17	0.02
	Albumin	-0.05	0.02	-2.19	0.07
	C-reactive protein	-0.02	0.01	-3.19	0.02
	Corrected R² = 0.587	F=3.485	p=0.073		
**Variables included**	**28-day mortality**, age, sex, aetiology of cirrhosis, ACLF precipitant, Sodium, Creatinine, Bilirubin, Albumin, C-reactive protein, Alkaline phosphatase, Haemoglobin, Time from ACLF to Death				
	**Results:**	Regr. Coeff.	SE	T	*p*
	28-day mortality	0.44	0.16	2.84	0.02
	ACLF precipitant	0.23	0.05	4.84	0.00
	Age	0.02	0.01	2.71	0.03
	Sodium	-0.04	0.01	-3.44	0.01
	Alkaline phosphatase	0.00	0.00	1.94	0.09
	Time from ACLF to Death	0.00	0.00	3.09	0.01
	Corrected R² = 0.651	F=5.348	p=0.017		

EBV DNAemia

**Variables included**	**90-day mortality**, sex, aetiology of cirrhosis, ACLF precipitant, Sodium, Creatinine, Bilirubin, Albumin, Leukocytes, C-reactive protein, Platelets, International normalized ratio, Gamma-glutamyltransferase, Time to death				
	**Results:**	Regr. Coeff.	SE	T	*p*

	ACLF precipitant	0.06	0.03	2.07	0.06
	International normalized ratio	-0.40	0.19	-2.14	0.05
	Corrected R^2 = 0.284	F=4.375	p=0.032		
**Variables included**	**28-day mortality**, sex, aetiology of cirrhosis, ACLF precipitant, Sodium, Creatinine, Bilirubin, Albumin, Leukocytes, C-reactive protein, Platelets, International normalized ratio, Gamma-glutamyltransferase, Time to death				
	**Results:**	Regr. Coeff.	SE	T	*p*
	Sex	0.36	0.07	5.25	0.00
	28-day mortality	0.35	0.06	6.34	0.00
	ACLF precipitant	0.23	0.02	10.33	0.00
	Sodium	-0.04	0.01	-5.01	0.00
	Creatinine	0.28	0.05	5.66	0.00
	Bilirubin	0.43	0.04	11.06	0.00
	Albumin	0.03	0.00	8.31	0.00
	Leukocytes	-0.10	0.01	-7.06	0.00
	C-reactive protein	0.00	0.00	2.95	0.03
	Platelets	0.00	0.00	3.77	0.01
	International normalized ratio	-1.54	0.13	-11.86	0.00
	Time to Death	0.00	0.00	5.47	0.00
	Corrected R^2 = 0.932	F=20.419	p=0.002		

CMV and/or EBV DNAemia

**Variables included**	**90-day mortality**, sex, aetiology of cirrhosis, ACLF precipitant, Sodium, Albumin, Platelets, Alkaline phosphatase, Bilirubin, International normalized ratio				
	**Results:**	Regr. Coeff.	SE	T	*p*
	Aetiology of cirrhosis	0.07	0.03	1.96	0.06
	Sodium	-0.03	0.01	-3.34	0.00
	International normalized ratio	-0.59	0.28	-2.12	0.05
	Corrected R^2 = 0.343	F=5.168	p=0.008		
**Variables included**	**28-day mortality**, sex, aetiology of cirrhosis, ACLF precipitant, Sodium, Albumin, Platelets, Alkaline phosphatase, Bilirubin, International normalized ratio				
	**Results:**	Regr. Coeff.	SE	T	*p*
	Aetiology of cirrhosis	0.07	0.03	1.96	0.06
	Sodium	-0.03	0.01	-3.34	0.00
	International normalized ratio	-0.59	0.28	-2.12	0.05
	Corrected R^2 = 0.343	F=5.168	p=0.008		

Tests vs. CMV DNAemia

**Pearson-Chi-Square test**<br>**Variable**	***p***	**Odds ratio**	**Lower 95% CI**	**Higher 95% CI**
pre-ACLF	0.37	1.96	0.45	8.57
Sex	0.41	0.50	0.09	2.67
28-day mortality	0.16	2.95	0.61	14.18
90-day mortality	0.31	2.19	0.47	10.29
Liver failure	1			

Renal failure	0.72 [2]
Cerebral failure	[1]
Coagulation failure	[1]
Circulation failure	[1]
Respiratory failure	[1]
Aetiology of cirrhosis	0.05 [3]
ACLF precipitant	0.19 [3]
Immunosuppression	[1]

Biserial rank-correlation

Variable	*p*	Corr. Coeff.	DF
West Haven	[1]		
MELD	0.38	0.54	73.00
CLIF-C AD	0.64	0.64	73.00
CLIF-C OF	[1]		
Child-Pugh category	0.18	0.14	73.00

Shapiro-Wilk test

Variables	*p*	W
Age	0.61	0.94
Sodium	0.48	0.93
Creatinine	0.99	0.99
Bilirubin	0.04	0.83
Albumin	0.49	0.93
International normalized ratio	0.02	0.79
Leukocytes	0.06	0.84
C-reactive protein	0.25	0.90
Aspartate aminotransferase	0.46	0.93
Alanine aminotransferase	0.59	0.94
Platelets	0.09	0.86
Time to ACLF	0.10	0.86
Time to death	0.00	0.67
Time ACLF to death	0.00	0.60

T-test

Variables	*p*	T	DF	Cohen's d
Age	0.22	-0.77	71.00	-0.29
Sodium	0.09	-1.34	71.00	-0.50
Creatinine	0.30	-0.54	71.00	-0.20
Albumin	0.19	-0.89	71.00	-0.33
Leukocytes	0.38	-0.31	71.00	-0.12
C-reactive protein	0.28	-0.59	71.00	-0.22
Aspartate aminotransferase	0.50	0.00	70.00	0.00
Alanine transaminase	0.45	0.13	69.00	0.05
Platelets	0.47	0.09	71.00	0.03
Time to ACLF	0.34	-0.41	24.00	-0.22

Mann-Whitney-U test

Variables	*p*	U	Z	r
Bilirubin	0.16	180.00	-1.41	0.17
International normalized ratio	0.81	246.50	-0.24	0.03
Total protein	0.38	83.50	-0.88	0.13
PTT	0.73	240.50	-0.35	0.04
Time to Death	0.60	46.50	-0.53	0.10
Time ACLF to Death	0.84	15.50	-0.20	0.05

Tests vs. EBV DNAemia

Pearson-Chi-Square test

Variable	*p*	Odds ratio	Lower 95% CI	Higher 95% CI
pre-ACLF	0.51	1.96	0.26	14.83
Sex	0.11 [2]			
28-day mortality	0.68	1.64	0.16	17.23
90-day mortality	0.87	1.21	0.12	12.60
Liver failure	[1]			

Renal failure	0.80	[2]		
Cerebral failure	[1]			
Coagulation failure	[1]			
Circulation failure	[1]			
Respiratory failure	[1]			
Aetiology of cirrhosis	0.12	[3]		
ACLF precipitant	0.36	[3]		
Immunosuppression	[1]			
**Biserial rank-correlation**				
**Variable**	***p***	**Corr. Coeff.**	**DF**	
West Haven	[1]			
MELD	0.409	0.03	69	
CLIF-C AD	0.530	0.76	69	
CLIF-C OF	[1]			
Child-Pugh category	0.135	0.28	69	
**Shapiro-Wilk test**				
**Variables**	***p***	**W**		
Age	0.47	0.93		
Sodium	0.67	0.95		
Creatinine	0.95	0.98		
Bilirubin	0.08	0.85		
Albumin	0.59	0.94		
International normalized ratio	0.05	0.82		
Leukocytes	0.11	0.86		
C-reactive protein	0.11	0.86		
Aspartate aminotransferase	0.34	0.91		
Alanine aminotransferase	0.77	0.96		
Platelets	0.16	0.87		
Time to ACLF	0.03	0.81		
Time to death	0.00	0.68		
Time ACLF to death	0.00	0.63		
**T-test**				
**Variables**	***p***	**T**	**DF**	**Cohen's d**
Age	0.41	-0.24	67.00	-0.12
Sodium	0.18	-0.94	67.00	-0.49
Creatinine	0.26	0.64	21.87	0.10
Bilirubin	0.00	-6.65	22.45	-1.03
Albumin	0.26	-0.65	67.00	-0.34
Leukocytes	0.21	0.80	67.00	0.41
C-reactive protein	0.24	-0.70	67.00	-0.36
Aspartate aminotransferase	0.35	0.38	64.00	0.27
Alanine transaminase	0.28	0.60	66.00	0.31
Platelets	0.02	2.07	67.00	1.07
**Mann-Whitney-U test**				
**Variables**	***p***	**U**	**Z**	**r**
International normalized ratio	0.01	23.50	-2.80	0.34
PTT	0.20	80.00	-1.29	0.15
Haemoglobin	0.69	75.00	-0.40	0.05
Time to ACLF	0.50	15.50	-0.68	0.14
Time to Death	0.10	0.00	-1.66	0.35
Time ACLF to Death	0.44	4.50	-0.78	0.18

Tests vs. CMV and/or EBV DNAemia

**Pearson-Chi-Square test**				
**Variable**	***p***	**Odds ratio**	**Lower 95% CI**	**Higher 95% CI**
pre-ACLF	0.70	1.30	0.33	5.11
Sex	0.22	0.38	0.07	1.91
28-day mortality	0.32	2.10	0.47	9.43
90-day mortality	0.55	1.561	0.357	6.832
Liver failure	[1]			

Renal failure	0.69 [2]			
Cerebral failure	[1]			
Coagulation failure	[1]			
Circulation failure	[1]			
Respiratory failure	[1]			
Aetiology of cirrhosis	0.17 [3]			
ACLF precipitant	0.19 [3]			
Immunosuppression	[1]			
**Biserial rank-correlation**				
**Variable**	***p***	**Corr. Coeff.**	**DF**	
West Haven	[1]			
MELD	0.40	0.29	75.00	
CLIF-C AD	0.62	0.74	75.00	
CLIF-C OF	[1]			
Child-Pugh category	0.15	0.20	75.00	
**Shapiro-Wilk test**				
**Variables**	***p***	**W**		
Age	0.61	**0.94**		
Sodium	0.48	0.93		
Creatinine	0.99	0.99		
Bilirubin	0.04	0.83		
Albumin	0.49	0.93		
International normalized ratio	0.02	0.79		
Leukocytes	0.06	0.84		
C-reactive protein	0.25	0.90		
Aspartate aminotransferase	0.46	0.93		
Alanine aminotransferase	0.59	0.94		
Platelets	0.09	0.86		
Time to ACLF	0.10	0.86		
Time to death	0.00	0.67		
Time ACLF to death	0.00	0.60		
**T-test**				
**Variables**	***p***	**T**	**DF**	**Cohen's d**
Age	0.33	-0.45	73.00	-0.15
Sodium	0.04	-1.79	73.00	-0.61
Creatinine	0.37	-0.33	73.00	-0.11
Albumin	0.14	-1.08	73.00	-0.37
Leukocytes	0.34	0.42	73.00	0.14
C-reactive protein	0.33	-0.44	73.00	-0.15
Aspartate aminotransferase	0.50	0.00	70.00	0.00
Alanine transaminase	0.36	0.37	71.00	0.13
Platelets	0.10	1.30	73.00	0.44
Time to ACLF	0.34	-0.41	24.00	-0.22
**Mann-Whitney-U test**				
**Variables**	***p***	**U**	**Z**	**r**
Bilirubin	0.03	184.50	-2.19	0.25
International normalized ratio	0.29	259.00	-1.05	0.12
Total protein	0.41	104.50	-0.82	0.12
PTT	0.71	301.50	-0.37	0.04
Time to Death	0.60	46.50	-0.53	0.10
Time ACLF to Death	0.84	15.50	-0.20	0.05

Supplementary sensitivity analyses

Table S8. ACLF-I sensitivity analyses for immunosuppression. Analyses separated by statistical method. Abbreviations: COPD = Chronic obstructive pulmonary disease, TIPS = Transjugular intrahepatic portosystemic stent, MELD = Model for End-Stage Liver Disease score, CLIF-C = Chronic Liver Failure Consortium, AD = Acute decompensation, OF = Organ failure, [1]calculation of Odds ratio not possible due to small patient groups; [2]calculation of Odds ratio due to table >2x2 not possible

**Tests vs. CMV DNAemia**				
**Pearson-Chi-Square test**				
**Variable**	***p***	**Odds ratio**	**Lower 95% CI**	**Higher 95% CI**
ACLF	0.00	4.13	1.64	10.41
Pre-ACLF	0.91	1.09	0.23	5.21
Sex	0.06	0.44	0.18	1.06
28-day mortality	0.50	1.57	0.41	5.98
90-day mortality	0.79	1.15	0.40	3.34
Liver failure	0.00	4.08	1.58	10.55
Renal failure	0.74	1.25	0.34	4.66
Cerebral failure	0.07	3.57	0.83	15.37
Coagulation failure	0.35	1.88	0.49	7.31
Circulation failure	0.83	1.16	0.31	4.30
Respiratory failure	0.66	1.65	0.18	15.44
Diabetes	0.18	0.50	0.18	1.41
COPD	0.33	[1]		
Heart failure	0.37	[1]		
Hypertension	0.03	0.21	0.05	0.93
Coronary artery disease	0.14	[1]		
Chronic kidney disease	0.43	[1]		
Ascites	0.55	1.32	0.53	3.27
Hepatic encephalopathy	0.15	1.93	0.78	4.77
Gastrointestinal bleeding	0.44	0.64	0.21	2.00
Bacterial infections	0.56	1.29	0.55	3.06
Viral infections	0.64	1.71	0.18	15.96
Vasopressors	0.14	3.46	0.60	19.99
Dialysis	0.49	[1]		
TIPS	0.24	0.42	0.09	1.87
Beta blockers	0.01	0.25	0.08	0.79
Antibiotic prophylaxis	0.43	1.51	0.54	4.23
Oxygen supplementation	0.20	2.19	0.65	7.40
Mechanical ventilation	0.29	2.07	0.53	8.17
Any oxygen supplement	0.49	2.22	0.22	22.23
Aetiology of cirrhosis	0.16	[2]		
**Biserial rank-correlation**				
**Variable**	***p***	**Corr. Coeff.**	**DF**	
ACLF grade	0.65	0.08	38	
West Haven	0.00	0.24	180	
MELD	0.46	0.25	46	
MELD sodium	0.07	0.46	173	
CLIF-C ACLF	0.11	0.52	173	
CLIF-C AD	0.08	0.51	179	
CLIF-C OF	0.02	0.64	179	
Child-Pugh score	0.01	0.32	180	
Child-Pugh category	0.10	0.18	180	
**Shapiro-Wilk test**				
**Variables**	***p***	**W**		
Age	0.05	0.97		
Sodium	0.05	0.97		

Creatinine	0.00	0.77		
Bilirubin	0.00	0.72		
Albumin	0.55	0.99		
International normalized ratio	0.00	0.78		
Leukocytes	0.00	0.87		
C-reactive protein	0.00	0.74		
Aspartate aminotransferase	0.00	0.60		
Alanine aminotransferase	0.00	0.66		
Alkaline phosphatase	0.00	0.65		
Gamma-glutamyltransferase	0.00	0.27		
Platelets	0.00	0.85		
**T-test**				
**Variables**	***p***	**DF**	**T**	**Cohen's d**
Sodium	0.38	178	-0.30	-0.07
Albumin	0.36	176	-0.36	-0.08
**Mann-Whitney-U test**				
**Variables**	***p***	**U**	**Z**	**r**
Age	0.14	1525.5	-1.46	0.11
Creatinine	0.74	1792.5	-0.33	0.02
Bilirubin	0.02	1311.5	-2.36	0.18
International normalized ratio	0.96	1860.0	-0.05	0.00
Leukocytes	0.01	1260.0	-2.54	0.19
C-reactive protein	0.23	1358.5	-1.21	0.09
Aspartate aminotransferase	0.01	739.5	-2.58	0.22
Alanine aminotransferase	0.15	1420.5	-1.45	0.11
Alkaline phosphatase	0.01	737.0	-2.51	0.21
Gamma-glutamyltransferase	0.02	1285.5	-2.32	0.17
Platelets	0.63	1444.5	-0.48	0.04
**Tests vs. EBV DNAemia**				
**Pearson-Chi-Square test**				
**Variable**	***p***	**Odds ratio**	**Lower 95% CI**	**Higher 95% CI**
ACLF	0.01	4.34	1.38	13.66
Pre-ACLF	0.28	[1]		
Sex	0.12	0.42	0.14	1.27
28-day mortality	0.88	0.85	0.10	6.99
90-day mortality	0.69	0.73	0.16	3.44
Liver failure	0.37	1.86	0.48	7.23
Renal failure	0.64	1.46	0.30	7.11
Cerebral failure	0.46	[1]		
Coagulation failure	0.99	1.01	0.12	8.48
Circulation failure	0.71	1.35	0.28	6.57
Respiratory failure	0.33	2.92	0.30	28.11
Diabetes	0.12	0.32	0.07	1.46
COPD	0.46	[1]		
Heart failure	0.44	2.32	0.25	21.40
Hypertension	0.21	0.39	0.08	1.80
Coronary artery disease	0.88	0.85	0.10	6.99
Chronic kidney disease	0.54	[1]		
Ascites	0.18	2.42	0.65	9.02
Hepatic encephalopathy	0.68	1.29	0.38	4.34
Gastrointestinal bleeding	0.42	0.54	0.12	2.51
Bacterial infections	0.33	1.73	0.57	5.21
Viral infections	0.56	[1]		
Vasopressors	0.02	6.33	1.05	38.17
Dialysis	0.22	3.92	0.38	40.44
TIPS	0.30	0.35	0.04	2.80
Beta blockers	0.19	0.45	0.13	1.53
Antibiotic prophylaxis	0.22	0.29	0.04	2.34
Oxygen supplementation	0.43	1.89	0.38	9.45
Mechanical ventilation	0.90	1.15	0.14	9.76

Any oxygen supplement	0.22	3.92	0.38	40.44
Aetiology of cirrhosis	0.92	2		

Biserial rank-correlation

Variable	*p*	Corr. Coeff.	DF
ACLF grade	0.45	0.31	34
West Haven	0.05	0.25	170
MELD	0.29	0.17	41
MELD sodium	0.45	0.36	167
CLIF-C ACLF	0.22	0.37	167
CLIF-C AD	0.15	0.39	169
CLIF-C OF	0.32	0.69	169
Child-Pugh score	0.84	0.29	170
Child-Pugh category	0.84	0.23	170

Shapiro-Wilk test

Variables	*p*	W
Age	0.42	0.98
Sodium	0.04	0.97
Creatinine	0.00	0.78
Bilirubin	0.00	0.72
Albumin	0.68	0.99
International normalized ratio	0.00	0.76
Leukocytes	0.00	0.86
C-reactive protein	0.00	0.75
Aspartate aminotransferase	0.00	0.60
Alanine aminotransferase	0.00	0.64
Alkaline phosphatase	0.00	0.65
Gamma-glutamyltransferase	0.00	0.29
Platelets	0.00	0.91

T-test

Variables	*p*	DF	T	Cohen’s d
Age	0.38	168	-0.32	-0.09
Albumin	0.23	20.22	-0.72	-0.14

Mann-Whitney-U test

Variables	*p*	U	Z	r
Sodium	0.09	795.0	-1.69	0.13
Creatinine	0.88	1066.0	-0.15	0.01
Bilirubin	0.38	936.5	-0.88	0.07
International normalized ratio	0.79	1044.0	-0.27	0.02
Leukocytes	0.02	684.0	-2.29	0.18
C-reactive protein	0.03	538.5	-2.24	0.18
Aspartate aminotransferase	0.54	511.5	-0.62	0.06
Alanine aminotransferase	0.09	647.0	-1.67	0.13
Alkaline phosphatase	0.13	512.0	-1.53	0.13
Gamma-glutamyltransferase	0.48	872.5	-0.70	0.05
Platelets	0.84	710.5	-0.20	0.02

Tests vs. CMV and/or EBV DNAemia

Pearson-Chi-Square test

Variable	*p*	Odds ratio	Lower 95% CI	Higher 95% CI
ACLF	0.00	3.68	1.65	8.22
Pre-ACLF	0.66	0.71	0.15	3.30
Sex	0.03	0.44	0.20	0.93
28-day mortality	0.60	1.38	0.42	4.49
90-day mortality	0.79	0.88	0.33	2.30
Liver failure	0.01	2.99	1.28	7.00
Renal failure	0.88	1.09	0.34	3.49
Cerebral failure	0.25	2.27	0.54	9.56
Coagulation failure	0.41	1.65	0.49	5.51
Circulation failure	0.98	1.02	0.32	3.22
Respiratory failure	0.35	2.24	0.39	12.71
Diabetes	0.08	0.46	0.19	1.11

COPD	0.23 [1]			
Heart failure	0.89	0.86	0.10	7.62
Hypertension	0.02	0.29	0.10	0.87
Coronary artery disease	0.25	0.31	0.04	2.48
Chronic kidney disease	0.33 [1]			
Ascites	0.31	1.50	0.69	3.26
Hepatic encephalopathy	0.39	1.42	0.64	3.15
Gastrointestinal bleeding	0.20	0.52	0.19	1.43
Bacterial infections	0.49	1.29	0.63	2.67
Viral infections	0.91	1.14	0.12	10.50
Vasopressors	0.10	3.46	0.74	16.17
Dialysis	0.75	1.46	0.15	14.43
TIPS	0.16	0.42	0.12	1.45
Beta blockers	0.02	0.35	0.15	0.84
Antibiotic prophylaxis	0.96	1.03	0.41	2.59
Oxygen supplementation	0.30	1.79	0.59	5.42
Mechanical ventilation	0.67	1.34	0.35	5.14
Any oxygen supplement	0.22	3.00	0.48	18.65
Aetiology of cirrhosis	0.32 [2]			

Biserial rank-correlation

**Variable**	***p***	**Corr. Coeff.**	**DF**
ACLF grade	0.75	0.11	43
West Haven	0.00	0.25	192
MELD	0.64	0.20	48
MELD sodium	0.10	0.42	185
CLIF-C ACLF	0.09	0.46	185
CLIF-C AD	0.04	0.44	191
CLIF-C OF	0.03	0.62	191
Child-Pugh score	0.06	0.29	192
Child-Pugh category	0.21	0.20	192

Shapiro-Wilk test

**Variables**	***p***	**W**
Age	0.24	0.98
Sodium	0.05	0.97
Creatinine	0.00	0.78
Bilirubin	0.00	0.73
Albumin	0.58	0.99
International normalized ratio	0.00	0.78
Leukocytes	0.00	0.88
C-reactive protein	0.00	0.76
Aspartate aminotransferase	0.00	0.60
Alanine aminotransferase	0.00	0.65
Alkaline phosphatase	0.00	0.65
Gamma-glutamyltransferase	0.00	0.28
Platelets	0.00	0.85

T-test

**Variables**	***p***	**DF**	**T**	**Cohen's d**
Age	0.15	45.95	-1.21	-0.22
Albumin	0.35	188	-0.38	-0.07

Mann-Whitney-U test

**Variables**	***p***	**U**	**Z**	**r**
Sodium	0.36	2533.5	-0.92	0.07
Creatinine	0.43	2569.5	-0.79	0.06
Bilirubin	0.05	2220.0	-1.96	0.14
International normalized ratio	0.91	2774.0	-0.11	0.01
Leukocytes	0.00	1941.0	-2.84	0.21
C-reactive protein	0.08	1883.0	-1.77	0.13
Aspartate aminotransferase	0.03	1193.0	-2.18	0.18
Alanine aminotransferase	0.80	2436.5	-0.26	0.02
Alkaline phosphatase	0.01	1207.0	-2.50	0.20
Gamma-glutamyltransferase	0.03	2041.0	-2.14	0.16

Platelets	0.49	1973.5	-0.70	0.05

Table S9. Validation cohort sensitivity analyses for immunosuppression. Analyses separated by statistical method. Abbreviations: MELD = Model for End-Stage Liver Disease score, CLIF-C = Chronic Liver Failure Consortium, AD = Acute decompensation, OF = Organ failure, [1]calculation of Odds ratio not possible due to small patient groups; [2]calculation of significance not possible as one variable is a constant; [3]calculation of Odds ratio due to table >2x2 not possible

**Tests vs. CMV DNAemia**				
**Pearson-Chi-Square test**				
**Variable**	***p***	**Odds ratio**	**Lower 95% CI**	**Higher 95% CI**
ACLF	0.00	40.63	12.04	137.10
pre-ACLF	0.06	3.61	0.90	14.45
Sex	0.14	0.52	0.22	1.25
28-day mortality	0.72	0.83	0.28	2.40
90-day mortality	0.84	0.92	0.39	2.17
Liver failure	0.01	10.50	1.06	104.42
Renal failure	0.00	24.32	9.26	63.87
Cerebral failure	0.16	[1]		
Coagulation failure	[2]			
Circulation failure	[2]			
Respiratory failure	[2]			
Precipitant	0.35			
ACLF precipitant	0.04	[3]		
**Biserial rank-correlation**				
**Variable**	***p***	**Corr. Coeff.**	**DF**	
ACLF grade	0.62	0.25	38	
West Haven	0.00	0.63	148	
MELD	0.86	0.15	148	
CLIF-C ACLF	0.00	0.68	148	
CLIF-C AD	0.11	0.74	38	
CLIF-C OF	0.46	0.56	110	
Child-Pugh category	0.00	0.64	111	
**Shapiro-Wilk test**				
**Variables**	***p***	**W**		
Age	0.45	0.99		
Sodium	0.00	0.94		
Creatinine	0.00	0.74		
Bilirubin	0.00	0.39		
Albumin	0.25	0.99		
International normalized ratio	0.00	0.86		
Leukocytes	0.00	0.89		
C-reactive protein	0.00	0.74		
Aspartate aminotransferase	0.00	0.45		
Alanine aminotransferase	0.00	0.59		
Platelets	0.00	0.89		
**T-test**				
**Variables**	***p***	**DF**	**T**	**Cohen's d**
Age	0.42	146	-0.21	-0.04
Albumin	0.29	143	-0.57	-0.11
**Mann-Whitney-U test**				
**Variables**	***p***	**U**	**Z**	**r**
Sodium	0.19	1684.5	-1.33	0.11
Creatinine	0.00	647.5	-6.00	0.49
Bilirubin	0.33	1763.5	-0.97	0.08
International normalized ratio	0.02	1479.5	-2.27	0.19
Leukocytes	0.75	1907.5	-0.32	0.03

C-reactive protein	0.08	1594	-1.73	0.14
Aspartate aminotransferase	0.06	1236	-1.86	0.16
Alanine aminotransferase	0.48	1735.5	-0.71	0.06
Platelets	0.22	1703.5	-1.24	0.10
**Tests vs. EBV DNAemia**				
**Pearson-Chi-Square test**				
**Variable**	***p***	**Odds ratio**	**Lower 95% CI**	**Higher 95% CI**
ACLF	0.00	46.88	9.50	231.32
pre-ACLF	0.09	4.33	0.68	27.35
Sex	0.02	0.20	0.04	0.88
28-day mortality	0.84	0.87	0.23	3.28
90-day mortality	0.81	1.13	0.40	3.21
Liver failure	0.67	[1]		
Renal failure	0.00	25.25	7.79	81.83
Cerebral failure	0.29	[1]		
Coagulation failure	[2]			
Circulation failure	[2]			
Respiratory failure	[2]			
Aetiology of cirrhosis	0.29	[3]		
ACLF precipitant	0.20	[3]		
**Biserial rank-correlation**				
**Variable**	***p***	**Corr. Coeff.**	**DF**	
ACLF grade	0.11	0.32	27	
West Haven	0.00	0.61	133	
MELD	0.77	0.18	133	
CLIF-C ACLF	0.00	0.70	133	
CLIF-C AD	0.01	0.92	27	
CLIF-C OF	0.63	0.47	106	
Child-Pugh category	0.00	0.63	96	
**Shapiro-Wilk test**				
**Variables**	***p***	**W**		
Age	0.71	0.99		
Sodium	0.00	0.93		
Creatinine	0.00	0.74		
Bilirubin	0.00	0.50		
Albumin	0.42	0.99		
International normalized ratio	0.00	0.87		
Leukocytes	0.00	0.89		
C-reactive protein	0.00	0.72		
Aspartate aminotransferase	0.00	0.44		
Alanine aminotransferase	0.00	0.57		
Platelets	0.00	0.89		
**T-test**				
**Variables**	***p***	**DF**	**T**	**Cohen's d**
Age	0.16	131	-1.02	-0.25
Albumin	0.49	128	0.02	0.01
**Mann-Whitney-U test**				
**Variables**	***p***	**U**	**Z**	**r**
Sodium	0.40	996	-0.85	0.07
Creatinine	0.00	316.5	-5.12	0.44
Bilirubin	0.81	1091.5	-0.24	0.02
International normalized ratio	0.55	1037	-0.59	0.05
Leukocytes	0.89	1107.5	-0.14	0.01
C-reactive protein	0.13	887	-1.53	0.13
Aspartate aminotransferase	0.03	555	-2.24	0.21
Alanine aminotransferase	0.28	942	-1.08	0.09
Platelets	0.20	927	-1.28	0.11
**Tests vs. CMV and/or EBV DNAemia**				
**Pearson-Chi-Square test**				

Variable	*p*	Odds ratio	Lower 95% CI	Higher 95% CI
ACLF	0.00	28.13	9.61	82.34
pre-ACLF	0.08	2.89	0.86	9.74
Sex	0.07	0.45	0.19	1.08
28-day mortality	0.84	0.90	0.33	2.45
90-day mortality	0.83	0.91	0.40	2.09
Liver failure	0.02	9.33	0.94	92.55
Renal failure	0.00	18.94	7.66	46.85
Cerebral failure	0.14	[1]		
Coagulation failure	[2]			
Circulation failure	[2]			
Respiratory failure	[2]			
Aetiology of cirrhosis	0.34	[3]		
ACLF precipitant	0.05	[3]		
**Biserial rank-correlation**				
**Variable**	***p***	**Corr. Coeff.**	**DF**	
ACLF grade	0.59	0.25	39	
West Haven	0.00	0.60	152	
MELD	0.88	0.17	152	
CLIF-C ACLF	0.00	0.65	152	
CLIF-C AD	0.08	0.74	39	
CLIF-C OF	0.78	0.52	113	
Child-Pugh category	0.00	0.62	114	
**Shapiro-Wilk test**				
**Variables**	***p***	**W**		
Age	0.51	0.99		
Sodium	0.00	0.94		
Creatinine	0.00	0.74		
Bilirubin	0.00	0.39		
Albumin	0.28	0.99		
International normalized ratio	0.00	0.86		
Leukocytes	0.00	0.89		
C-reactive protein	0.00	0.74		
Aspartate aminotransferase	0.00	0.46		
Alanine aminotransferase	0.00	0.60		
Platelets	0.00	0.89		
**T-test**				
**Variables**	***p***	**DF**	**T**	**Cohen's d**
Age	0.42	150	-0.194	-0.036
Albumin	0.25	147	-0.666	-0.125
**Mann-Whitney-U test**				
**Variables**	***p***	**U**	**Z**	**r**
Sodium	0.19	1891.5	-1.32	0.11
Creatinine	0.00	824	-5.82	0.47
Bilirubin	0.43	2017	-0.79	0.06
International normalized ratio	0.05	1741	-1.97	0.16
Leukocytes	0.93	2182	-0.09	0.01
C-reactive protein	0.06	1764	-1.85	0.15
Aspartate aminotransferase	0.03	1270.5	-2.13	0.18
Alanine aminotransferase	0.28	1863	-1.07	0.09
Platelets	0.27	1942.5	-1.10	0.09

Table S10. Missing data for ACLF-I and Validation cohort. Number of missing data points and percentage of the respective cohort.

**ACLF-I**	
ACLF	0
Pre-ACLF	0
Sex	0

28-day mortality	0
90-day mortality	0
Liver failure	0
Renal failure	0
Cerebral failure	0
Coagulation failure	0
Circulation failure	1 (0.5)
Respiratory failure	0
Diabetes	0
COPD	0
Heart failure	0
Hypertension	0
Coronary artery disease	0
Chronic kidney disease	0
Ascites	0
Hepatic encephalopathy	0
Gastrointestinal bleeding	0
Bacterial infections	0
Viral infections	4 (1.9)
Vasopressors	0
Dialysis	0
TIPS	0
Immunosuppression	13 (6.2)
Beta blockers	12 (5.7)
Antibiotic prophylaxis	12 (5.7)
Oxygen supplementation	10 (4.7)
Mechanical ventilation	0
Any oxygen supplement	0
Aetiology of cirrhosis	0
ACLF grade	0
West Haven	0
MELD	8 (3.8)
MELD sodium	8 (3.8)
CLIF-C ACLF	1 (0.5)
CLIF-C AD	1 (0.5)
CLIF-C OF	0
Child-Pugh score	0
Child-Pugh category	0
Age	0
Albumin	2 (0.9)
Sodium	0
Creatinine	0
Bilirubin	0
International normalized ratio	0
Leukocytes	1 (0.5)
C-reactive protein	15 (7.1)
Aspartate aminotransferase	54 (25.6)
Alanine aminotransferase	8 (3.8)
Alkaline phosphatase	38 (18.0)
Gamma-glutamyltransferase	7 (3.3)
Platelets	0

**Validation cohort**	
ACLF	0
pre-ACLF	0
Sex	0
28-day mortality	0
90-day mortality	0
Liver failure	0
Renal failure	0
Cerebral failure	0
Coagulation failure	0
Circulation failure	0
Respiratory failure	0
Precipitant	0
ACLF precipitant	6 (8.5)
ACLF grade	0
West Haven	0
MELD	0
CLIF-C ACLF	0
CLIF-C AD	0
CLIF-C OF	0
Child-Pugh category	0
Age	0
Sodium	0
Creatinine	0
Bilirubin	0
Albumin	3 (2.0)
International normalized ratio	0
Leukocytes	0
C-reactive protein	0
Aspartate aminotransferase	19 (12.4)
Alanine aminotransferase	3 (2.0)
Platelets	0

Supplementary methods

Information for in-house CMV and EBV qPCR experiments

Sample collection, storage and general handling

211 patients admitted to the Department of Internal Medicine I, University Hospital Frankfurt, Germany, from January 2021 to June 2023 were enrolled in the ACLF-I cohort. Serum samples of respective patients were collected at the time of inclusion. Likewise, 78 patients admitted to the Department of Internal Medicine III, University Hospital Aachen and Department of Internal Medicine IV, Jena University Hospital, from September 2010 to July 2019 and 75 patients admitted to the Medical Clinic and Polyclinic I, University Hospital Bonn, from June 2019 to April 2023 were collected as part of one external validation cohort. Blood sera were collected in all three centers according to standardized clinical practice. All collected samples were aliquoted and stored at -80°C immediately. During transport samples were maintained frozen. All samples underwent a minimal number of freeze-thaw cycles due to previous aliquoting of samples. Moreover, samples were thawed on ice to minimize the impact of freeze-thawing. Since viral DNA is stable for a largely extended time-period without major loss at applied storage conditions, as previously shown [1], we determined viral DNA and not the amount of infectious particles. DNA extraction efficiency, amplification efficiency, DNA contaminations and inter-assay variations were monitored and validated as described in the following sections.

Nucleic Acid Extraction

Total viral nucleic acids within patient sera were extracted using the High Pure Viral Nucleic Acid Kit (Roche Diagnostics). The manufacturer´s instructions was modified as follows. 200 µl of respective patient serum was mixed with 200 µl Binding buffer supplemented with 4 µl poly A, 50 µl Proteinase K and 10 µl of Phocine herpesvirus

(PhHV) as extraction control. PhHV provided with the Lightmix Modular PhHV spiked Extraction Control (660) kit (Roche Diagnostics) was prepared according to manufacturer's instructions. After addition to sera, the reaction mixture was vortexed briefly and incubated for 30 min at 72°C. Subsequent extraction was performed according to instructions of the kit´s manufacturer. For elution, elution buffer was preheated to 70°C and viral nucleic acids were eluted in 50 µl of elution buffer. Extracted viral nucleic acids were stored immediately at -20°C and processed within two weeks. Due to limited sample size extracted viral nucleic acids were not quantified but totally processed in subsequent qPCR experiments. DNA or RNA contamination of extracted samples were assed by qPCR (see qPCR validation and data analysis).

qPCR oligonucleotides and target information

For all qPCR experiments different hydrolysis probes targeting either viral DNA of human Cytomegalovirus (CMV) or Epstein-Barr virus (EBV) and the PhHV extraction control were used in a multiplex assay. All hydrolysis probe kits were obtained by Roche diagnostics. Respective hydrolysis probes, the targeting regions and the length of the amplicon are depicted in table S11. Primer sequences and exact targeting regions are not available.

Table S11: Hydrolysis probes and corresponding amplicon details.

**Kit name**	**Cat no.**	**Amplicon location**	**Amplicon length**
Lightmix® Modular PhHV spiked Extraction Control (660)	07093802001	N/A	85 bp
Lightmix® Modular Epstein-Barr virus (610)	10097710001	BNRF1 gene	78 bp

Lightmix® Modular human<br>Cytomegalovirus (500)	08997837001	US17 gene<br>F fragment gene	151 bp<br>61 bp

qPCR protocol

All qPCR experiments were performed manually as multiplex-assay. The qPCR reaction for mixture was prepared as follows: 4 µl of Roche Lightcycler Multiplex RNA Virus/ DNA master (Roche Diagnostics) was mixed with 0.5 µl of respective hydrolysis probes (Table S2) and 2.5 µl of PCR-grade water (Roche Diagnostics) to a final volume of 8 µl per reaction. Then, 8 µl of the master mix was added per well of a white lightcycler 480 Multiwell plate (Roche Diagnostics). Finally, 12 µl of previously eluted viral nucleic acid extracts were added per well. All samples were measured in duplicate. In each qPCR run a positive control for CMV and EBV provided with the hydrolysis probe kits was run alongside to asses inter-assay variations. The multiplex qPCR was carried out in a Lightcycler 480 instrument II (Roche Diagnostics). The qPCR program consisted of an initial denaturation step at 95°C for 5 min and subsequent 45 cycles with denaturation at 95°C for 5 s, annealing at 60°C for 15 s and elongation at 72°C for 15 s with a ramp rate of 4.4, 2.2 and 4.4 respectively. After the final cycle the system was cooled down to 4°C. The CMV specific hydrolysis probe was detected using a 440-488 nm filter, a quant factor of 10 and a maximal integration time of 1 sec. The EBV specific hydrolysis probes was detected using a 533-610 nm filter with a quant factor of 10 and a maximum integration time of 2 sec. The PhHV specific hydrolysis probes were detected using a 618-660 nm filter, a quant factor of 10 and a maximum integration time of 3 sec.

qPCR validation and data analysis

Since the qPCR experiments were performed as multiplex-assays, preliminary a color compensation file was generated using the positive controls provided with the respective hydrolysis probe kits. The color compensation was applied to all subsequent qPCR experiments prior to analysis. Ct values were determined by applying the Second Derivative maximum algorithm using the Lightcycler® 480 software version 1.5.1 (Roche Diagnostics). To determine the lower limit of detection (LLOD) a dilution series ranging from 2 to 10^6 of CMV and EBV target molecules provided with the hydrolysis probe kits was performed. The CV values for each dilution series was assessed. 7 total copies per well resulted in successful detection with more than 95% confidence and a CV of 0.92% and 1.26% for the Ct values of CMV and EBV respectively. Thus, 7 copies per well were determined as the LLOD for CMV and EBV detection. The linear range with high coefficient of determination (R^2) for the linear regression models was determined as 10 to 10^6 target molecules per well for CMV and EBV. The coefficient of determination R^2 values of the linear regression models were 0.9998 and 0.9958 for CMV and EBV respectively. Thus, 10 target molecules per well were set as the lower limit of quantification (LLOQ) and 10^6 as the upper limit of quantification (ULQ) for CMV and EBV detection. Further, for CMV the qPCR efficiency was 99.33% and the amplification factor was 1.99. Accordingly, for EBV qPCR efficiency was 99.13% and the amplification factor was 1.99. The linear regression models with respective linear functions including slope and Y-intercept are depicted in Figure S1.

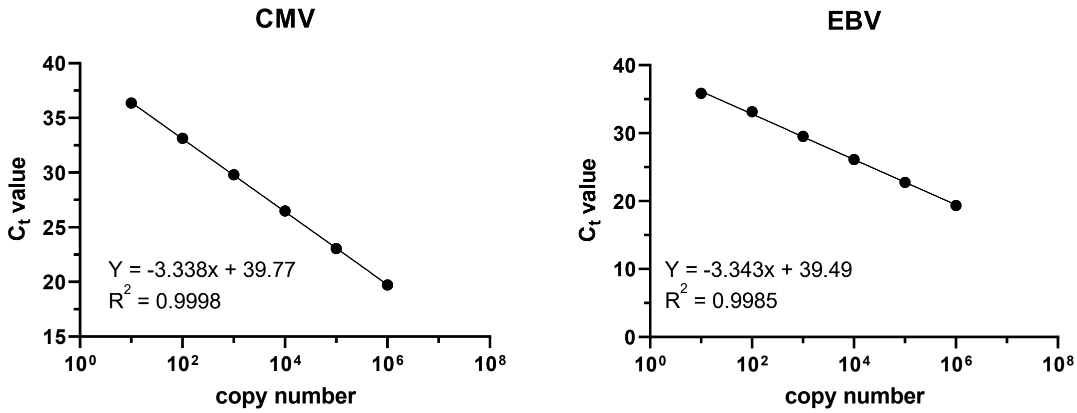

Fig. S1: Linear regression models of CMV and EBV standard curve for absolute quantification.

Ct values were converted into total copy number per well by extrapolation using the linear regression models. Viral loads were calculated by dividing the calculated copy numbers with the qPCR sample volume (12 µl) and by dividing with the concentration factor during viral nucleic acid extraction (for-fold concentration). Accordingly, 7 copies per well corresponding to the LLOD were converted into 150 copies/ml. Thus, 150 copies/ ml were set as the cut-off value for CMV and EBV detection. In this regards, all samples below the cut-off value were considered negative and conclusively non DNAemic. To assess nucleic acid contamination, the elution buffer which was used during viral nucleic acid extraction was measured with each qPCR run. All qPCR experiments in which the elution buffer was below the LLOD in multiple replicates were considered valid. To validate viral nucleic acid extraction quality, the Ct value of the PhHV extraction controls were determined. All samples with a Ct-value ranging between 27 and 30 for the PhHV extraction control were considered acceptable and thus included in subsequent analysis. The coefficient of variation (CV) of the Ct values for the PhHV extraction control for all samples within the ACLF-I cohort was 4.7%. The CV of the Ct values from the Aachen/Jena validation cohort was 4.9% and from the second validation cohort 3.1% for the extraction control. To asses inter-assay

variations for CMV and EBV viral nucleic acid detection, the CV of the Ct-value for the CMV and EBV positive controls run alongside each qPCR run was determined. Within the ACLF-I cohort the CV values were 1.69% for CMV and 0.94% for EBV positive controls. Within the Aachen/ Jena validation cohort the CV values were 1.3% for CMV and 0.44% for EBV positive controls. Within the Bonn validation cohort the CV values were 0.5% for CMV and 1.0% for EBV positive controls.

Experimental design

CMV and EBV DNAemia within all patient samples of the ACLF-I and the validation cohort were determined by the above mentioned qPCR method. Upon applying the quality control criteria the calculated DNAemia of all included patients were correlated to their respective clinical data. Presence of DNAemia (CMV and EBV combined or solely) within each subgroup (table S1) was determined as a proportion of the total number of each subgroup (Fig 1C and Fig 4A). Likewise, to asses DNAemia associated organ failure and inflammation the DNAemia detected proportion within the total collective was calculated (Fig 1.D, Fig 2 & Fig. 3) and correlated to respective parameters describing either organ failure or inflammation. The impact of DNAemia on the ACLF grade was analyzed by calculating the proportion of DNAemic patients within each ACLF grade subgroup. The total number of each subgroup and the proportion of DNAemic patients are indicated in table 1. All statistical methods to analyze significance are described in the materials and method section of the manuscript.

Cytokine analysis – Data analysis and validation

For cytokine profiling of respective serum samples a custom multiplex magnetic bead panel using the Luminex Discovery Assay Human Premixed Multi-Analyte Kit (Luminex Corporation) was performed. The multiplex assay was carried out as described in the materials and methods section. Each sample was measured in duplicates. A three fold

dilution series with six spiked standards of positive controls for respective cytokines and corresponding negative controls were measured alongside with each run to asses intra- and inter-assay variations. Mean CV values for an intermediate positive control within the linear range of each target cytokine are depicted in table S2. Runs with a CV below 15% for each positive control were considered valid. Accordingly, samples with a CV above 15% were excluded to maintain assay accuracy. Limit of detection was defined by a minimum of 50 magnetic beads bound to the target analyte. Displayed final Log_2 Fold changes were determined by calculating the binary logarithm of background subtracted fluorescence intensities normalized to respective negative controls corresponding to each run.

Table S12: Averaged CV and recovery rates of target analytes.

Target analyte	n	average CV (%)	average recovery (%)
CCL2/MCP-1	3	1.00	98.32
CCL3/MIP-1a	3	2.73	100.76
CCL4/MIP-1b	3	0.34	100.15
CXCL10/IP-10	3	2.13	100.19
GM-CSF	3	2.10	100.30
IFN-gamma	3	2.16	99.19
IL-10	3	3.99	101.86
IL-18	3	4.55	102.12
IL-1a	3	1.49	101.40
IL-1b	3	2.34	99.05
IL-1ra	3	2.70	98.92
IL-2	3	0.84	99.50
MIF	3	1.33	100.05
TNF-a	3	1.33	100.56

Supplementary references

1. Bajaj, J.S., et al., *Survival in infection-related acute-on-chronic liver failure is defined by extrahepatic organ failures.* Hepatology, 2014. **60**(1): p. 250-6.
2. Giamarellos-Bourboulis, E.J., et al., *Interferon-gamma driven elevation of CXCL9: a new sepsis endotype independently associated with mortality.* EBioMedicine, 2024. **109**: p. 105414.
3. Berres, M.L., et al., *CXCL9 is a prognostic marker in patients with liver cirrhosis receiving transjugular intrahepatic portosystemic shunt.* Journal of Hepatology, 2015. **62**(2): p. 332-339.
4. Noor, M.T. and P. Manoria, *Immune Dysfunction in Cirrhosis.* J Clin Transl Hepatol, 2017. **5**(1): p. 50-58.
5. Jalan, R., et al., *Development and validation of a prognostic score to predict mortality in patients with acute-on-chronic liver failure.* J Hepatol, 2014. **61**(5): p. 1038-47.
